# CRISPR/Cas9-induced double-strand breaks in the huntingtin locus lead to CAG repeat contraction through DNA end resection and homology-mediated repair

**DOI:** 10.1186/s12915-024-02079-6

**Published:** 2024-12-03

**Authors:** Pawel Sledzinski, Mateusz Nowaczyk, Marianna Iga Smielowska, Marta Olejniczak

**Affiliations:** Department of Genome Engineering, Institute of Bioorganic Chemistry, Polish Academy of Sciences, 61-704 Poznan, Poland

**Keywords:** DNA repair, Genome editing, Short tandem repeats, TMEJ

## Abstract

**Background:**

The expansion of CAG/CTG repeats in functionally unrelated genes is a causative factor in many inherited neurodegenerative disorders, including Huntington’s disease (HD), spinocerebellar ataxias (SCAs), and myotonic dystrophy type 1 (DM1). Despite many years of research, the mechanism responsible for repeat instability is unknown, and recent findings indicate the key role of DNA repair in this process. The repair of DSBs induced by genome editing tools results in the shortening of long CAG/CTG repeats in yeast models. Understanding this mechanism is the first step in developing a therapeutic strategy based on the controlled shortening of repeats. The aim of this study was to characterize Cas9-induced DSB repair products at the endogenous *HTT* locus in human cells and to identify factors affecting the formation of specific types of sequences.

**Results:**

The location of the cleavage site and the surrounding sequence influence the outcome of DNA repair. DSBs within CAG repeats result in shortening of the repeats in frame in ~ 90% of products. The mechanism of this contraction involves MRE11-CTIP and RAD51 activity and DNA end resection. We demonstrated that a DSB located upstream of CAG repeats induces polymerase theta-mediated end joining, resulting in deletion of the entire CAG tract. Furthermore, using proteomic analysis, we identified novel factors that may be involved in CAG sequence repair.

**Conclusions:**

Our study provides new insights into the complex mechanisms of CRISPR/Cas9-induced shortening of CAG repeats in human cells.

**Supplementary Information:**

The online version contains supplementary material available at 10.1186/s12915-024-02079-6.

## Background

Repetitive sequences are abundant in eukaryotic genomes, constituting more than half of the total nuclear DNA content in most species [1]. They are inherently susceptible to length polymorphisms that, in addition to their proposed roles in genome and protein evolution [2], may also lead to disease [3]. Expanded CNG trinucleotide repeats have been found to contribute to neurodegenerative disorders, e.g., Huntington’s disease (HD), spinocerebellar ataxias (SCAs), and myotonic dystrophy type 1 (DM1) [3]. In the case of HD, the disease manifests when the length of the CAG repeats in the first exon of the *HTT* gene reaches 40 or more [4]. It was suggested 20 years ago that double-strand break (DSB) repair is involved in trinucleotide repeat contractions and expansions [5]. Many subsequent studies corroborated this observation [6–11]. Long CAG repeats are often sites of chromosome breakage in yeast when DNA replication is slowed [12]. DSBs can also arise from unligated nicks or collapsed forks because structure-forming strands containing repeats promote DSBs during replication or transcription (reviewed in [3]). Additionally, DSBs may occur in mature neurons not only as a consequence of oxidative stress and transcription errors but also as a natural response to neuronal stimulation (reviewed in [13]).

DSBs are repaired by classical nonhomologous end joining (NHEJ) or homology-mediated repair, which includes homologous recombination (HR) (reviewed in [14]), single-strand annealing (SSA) (reviewed in [15]), microhomology-mediated end joining (MMEJ), and break-induced replication (BIR) (reviewed in [16, 17]). All the pathways listed above require a DNA end resection process to uncover the regions of homology; however, the degree of resection and homology size differ depending on the repair pathway [18, 19].

With the invention of genetic engineering techniques, especially the recent CRISPR-Cas revolution, there have been attempts to use these new tools to explore the molecular mechanism responsible for repeat instability as well as to shorten mutated repeats. Even a small reduction in tract length or in the probability of expansion may have significant implications for the prevention and treatment of repeat expansion diseases. Early studies on this topic revealed many interesting phenomena closely related to the properties of the repeated sequences, e.g., induction of DSBs within CAG or CTG tracts by SpCas9 or ZF nucleases led to both expansions and contractions of the repeats [11, 20], whereas single-strand cuts generated by Cas9 nickase resulted in contractions of CAG tracts [6]. In the latter study, the authors suggested that ATR kinase plays a role in preventing tract instability and that ATM kinase is involved in promoting contractions. They also observed that the instability induced by ATR inhibition is dependent on MSH2- and XPA-related activity [6]. In a yeast model, a break introduced in a CTG tract by TALEN or SpCas9 endonuclease leads to contraction of repeats below the pathological length [7, 8]. In search of the mechanism responsible for the observed phenomenon, the authors found that bidirectional resections occur and that Rad50 is essential for the process, whereas Sae2 (the yeast homolog of CTIP) is required to resect the DNA end containing most of the repetitive tract. Knockout of *RAD51*, *POL32*, or *DNL4* did not impact the repair process. The authors hypothesized that both the NHEJ and SSA pathways are involved in the process of repeat shortening, and that the removal of secondary structures formed at DSBs requires the activity of Sae2 and the Mre11-Rad50 complex [7]. Unexpectedly, the repair of DSBs generated large chromosomal deletions around the cutting site [8]. Later, the authors demonstrated the use of TALEN nuclease to induce a DSB in the CTG repeat tract to contract it below pathological length in the *DMPK* gene in human DM1 cells [21].

Interestingly, even a single DSB in a flanking region of the expanded CGG repeats in the *FMR1* gene and expanded CTG repeats in the DM1 locus were found to be sufficient to induce uncontrollable deletion of the entire tract in patient cells [9]. Several in vivo studies have demonstrated successful CRISPR-Cas9-mediated excision of CAG/CTG repeats (along with flanking sequences), using a dual-cutting strategy [22–26]. In the case of the HD model, this resulted in selective or nonselective knockout of the mutant *HTT* gene. The idea of contracting the expanded repeats has also been tested in animal models, but thus far to a limited extent [27, 28]. Still little is known about the mechanisms leading to repeat instability as a result of DSB repair. This knowledge is necessary to develop more effective and specific genome editing tools and to predict and control expansions and contractions of repeated sequences.

Here, we try to identify the key mechanisms responsible for the shortening of the CAG tract by studying the phenomena that occur after SpCas9-mediated induction of DSBs in the CAG microsatellite region of the *HTT* gene. In contrast to the approach taken in previous work in this field, we used an endogenous model of the human *HTT* gene without altering its genomic context. In addition, we applied techniques allowing an unbiased study of the proteins involved in the regulation of CAG tract instability.

We showed that the endonuclease cleavage site and the DSB flanking sequence play a key role in the selection of the DNA repair mechanism, resulting in different editing outcomes. DNA end resection is a common step in the repair of the CAG repeat region. Our results suggest that DSBs located upstream of CAG repeats induce polymerase theta (POLθ)-mediated end joining (TMEJ), resulting in deletion of the entire CAG tract. In contrast, DSBs within CAG repeats result in shortening of the repeats in frame through a mechanism dependent on MRE11-CTIP and RAD51 activity. These findings shed new light on the process of DSB repair and microsatellite instability in human cells.

## Results

### DSB location affects CAG repeat region editing results

**Fig. 1:**
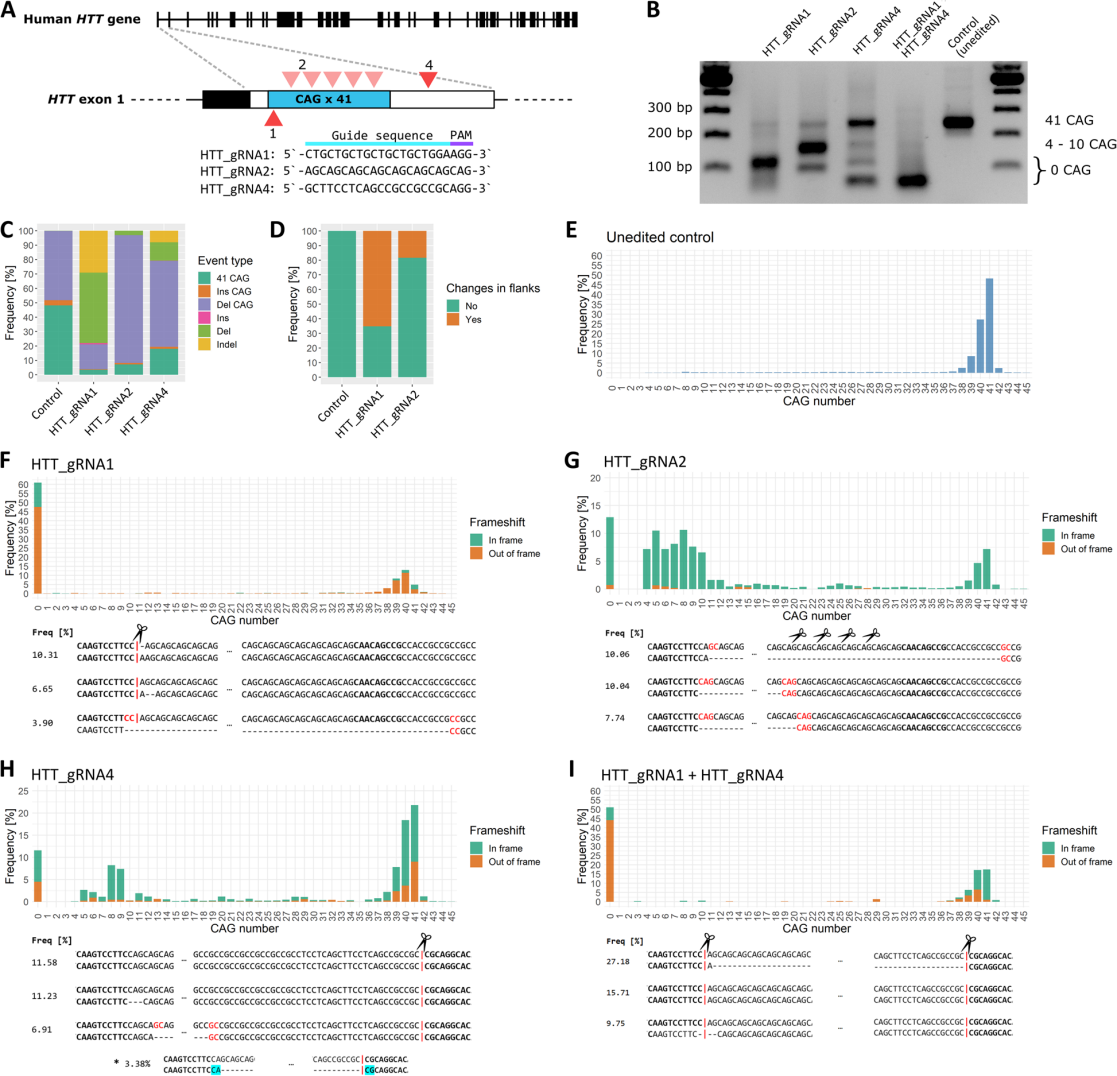
Deep sequencing analysis of CAG repetitive tract shortening induced by CRISPR-Cas9 cleavage. **A** Schematic depiction of the CAG tract in the *HTT* gene, with the editing sites marked in red. **B** Gel electrophoresis of DNA after editing. Different patterns of shortening are generated by each gRNA used. The DNA ladder used was a Perfect 100 bp DNA Ladder (EURX). **C** CRISPR-Cas9 editing outcomes divided into six main categories and presented as a bar plot comparing their proportions: 41 CAG—unedited original sequence, Ins CAG—increase in the number of CAG triplets (no frameshift), Del CAG—decrease in the number of CAG triplets (no frameshift), Ins—insertions leading to frameshift, Del—deletions leading to frameshift, Indel—composite changes leading to frameshift. **D** CRISPR-Cas9 editing outcomes presented as proportions of edits involving changes in the regions flanking the CAG tract and changes in the tract only. We did not include the results of HTT_gRNA4 and HTT_gRNA1 + HTT_gRNA4 editing, as these variants target the flank itself. **E**–**I** CAG tract length distribution among reads presented as the number of triplets and the presence of frameshifts. The three most frequent results are presented below the respective graphs. The microhomologous regions that play a role in the tract contraction are marked in red. “*” in **H** denotes an excision of the entire tract, from “G” of the first CAG triplet to the HTT_gRNA4 cutting site

Since both the length of the CAG repeat sequence and its location in the genome can influence DNA repair mechanisms, in our study, we used an endogenous *HTT* locus with a repeat sequence length characteristic of HD patients. As a model, we used a previously generated HEK293T cell line with 41 CAG repeats in both alleles of the *HTT* gene (HEK293T-41CAG) [29]. Although HD patients are heterozygous, a homozygous model is more useful for studying changes in CAG repeat length. Notably, HEK293T cells do not express the MLH1 and PMS2 proteins, which are important for mismatch repair (MMR) [30]. Since these proteins are known drivers of repeat expansion, this may contribute to the lack of expansion in our model. DSBs were induced by SpCas9 and gRNAs described previously [31, 32], which utilize PAM (NGG) sequences located in regions flanking CAG repeats (HTT_gRNA1 and HTT_gRNA4) or directly targeting CAG repeats (HTT_gRNA2) (Fig. 1A). HTT_gRNA2 was designed to use a noncanonical PAM sequence (NAG). Notably, the DSB generated by gRNA1 is located just upstream of the CAG sequence, whereas gRNA2 can cleave at multiple locations in the CAG sequence. gRNA4 cleaves 58 nt downstream of the CAG repeats, in the GC-rich region of the gene. HEK293T-41CAG cells were transfected with RNP complexes containing both the SpCas9 protein and HTT_gRNA. Genomic DNA was isolated 24 h post-transfection. The region of interest was amplified by PCR and subjected to deep sequencing. We observed a shortening of the CAG tract both in agarose gel (Fig. 1B) and in the NGS results (Fig. 1C–I). The maximum read length of ~ 250 bp limits the possibility of assessing the occurrence of long expansions. The same is true for gel electrophoresis, which, although it did not show any longer products after editing, is not a reliable test for detecting expansions (Fig. 1B). The short, exact (CAG)_n_ indels observed in unedited samples are most likely stutter products generated during repetitive sequence amplification [33] (Fig. 1E). This phenomenon was included in the outcome analysis.

We divided the NGS results into six main categories: “41 CAG,” denoting an unedited original sequence; “Ins CAG” and “Del CAG,” indicating an increase and decrease in the number of CAG triplets, respectively (these changes were “clean,” no frameshift occurred); “Ins” and “Del,” denoting insertions and deletions leading to frameshift; and “Indel,” a category encompassing all composite changes leading to frameshift.

After the introduction of DSBs using HTT_gRNA1, tract shortening was efficient, as the full-length 41 CAG tract was present in only 4.87% of the reads (Fig. 1C). Seventy-nine percent of edits resulted in a frameshift. We observed a predominance of deletions (66%), including out-of-frame Del (49%) and “clean,” in-frame Del CAG (17%). The Indel group composed of combined outcomes constituted 28% (Fig. 1C). The percentage of edits leading to changes in the flanks was 65% (Fig. 1D). The single DSB at the beginning of the tract introduced by HTT_gRNA1 was sufficient to induce robust excision of the entire CAG tract, as indicated by 61% of the reads (Fig. 1F). It was, however, not a “clean” excision, as in most cases, the deletion additionally spanned several nucleotides of the flanking regions on both sides of the CAG repeats. Interestingly, the single most abundant editing result was the insertion of a single A nucleotide, which occurred in 10.31% of the reads (Fig. 1F). This outcome can be interpreted as a result of Cas9 cleavage in a “staggered” fashion [34]. This occurs when the RuvC domain cuts the nontarget strand after the 4th nucleotide upstream of the PAM, which may subsequently lead to a duplication of the 4th nucleotide. A substantial group of reads comprises sequences resulting from the deletion of the CAG repeat region between short microhomologous sequences, most frequently CC:GG (see Additional file 1). Additionally, a portion of the products contained duplicated sequences from the upstream flank of the CAG repeats, potentially indicating polymerase theta activity [35, 36].

In the case of HTT_gRNA2, CAG repeat shortening was also efficient since the full-length tract constituted 7% of the reads (Fig. 1C). The majority of the edit outcomes were “clean” in frame Del CAG (89%). The out-of-frame Del was identified in only 3% of the reads. Accordingly, the proportion of outcomes containing changes in the flanks was low (18%, Fig. 1D). Considerable CAG tract shortening was observed, but contrary to HTT_gRNA1, the results were more variable in terms of tract length distribution (Fig. 1G). Deletions of the entire CAG region comprised 13% of the sequencing reads, and this group included the most frequent variant (~ 10%). In contrast to editing using HTT_gRNA1, excisions of only parts of the repetitive tract were frequent. Within this group, sequences with 4–10 CAG repeats predominated. Notably, editing with HTT_gRNA2 resulted in a substantially lower incidence of frameshifts (3%) than editing with HTT_gRNA1 (Fig. 1G). The “staggered cut” signature was not observed.

Editing with HTT_gRNA4 led to an interesting phenomenon, as despite targeting the region 58 base pairs downstream of the CAG tract, it generated a significant amount of “clean” in-frame Del CAG (60%, Fig. 1C). At the same time, it was the least efficient gRNA, as 13% of the reads contained full-length CAG tracts, and the most frequent variant was the unedited 41 CAG sequence (11.58%, Fig. 1H). A relatively abundant fraction of edits comprised short deletions, e.g., one CAG triplet deletion was the second most frequently detected variant (11.23%). The entire tract excision constituted 12% of all reads. Due to the nature of the edit, out-of-frame Del and complex Indels (18% and 4%, respectively) were more frequent than in the case of HTT_gRNA2 but less frequent than in the case of HTT_gRNA1 (Fig. 1C). An interesting outcome that we observed was the excision of the entire tract, from “G” of the first CAG triplet to the HTT_gRNA4 cutting site, which appeared with a frequency of 3.38% (Fig. 1H). The same editing outcome was observed in our previous work with the use of paired nickases and a combination of HTT_sgRNA1 and HTT_sgRNA4 [31]. Frameshift was considerably less frequent than in the case of HTT_gRNA1 (26%) (Fig. 1H).

The last analyzed variant was a result of simultaneous editing with HTT_gRNA1 and HTT_gRNA4 (Fig. 1I). As expected, the excision of the entire CAG tract was efficient (51%). The most frequent variant was a Del variant spanning the entire region between cleavage sites (27.18% of all reads). The frameshift frequency was 60% (Fig. 1I).

We decided to use only HTT_gRNA1 and HTT_gRNA2 in subsequent experiments, as they were more efficient than HTT_gRNA4 in the induction of CAG tract shortening and led to more mechanistically interesting phenomena than the simple fragment excision observed when HTT_gRNA1 and HTT_gRNA4 were used simultaneously.

### DNA end resection is involved in the process of DSB repair within CAG repeats

**Fig. 2:**
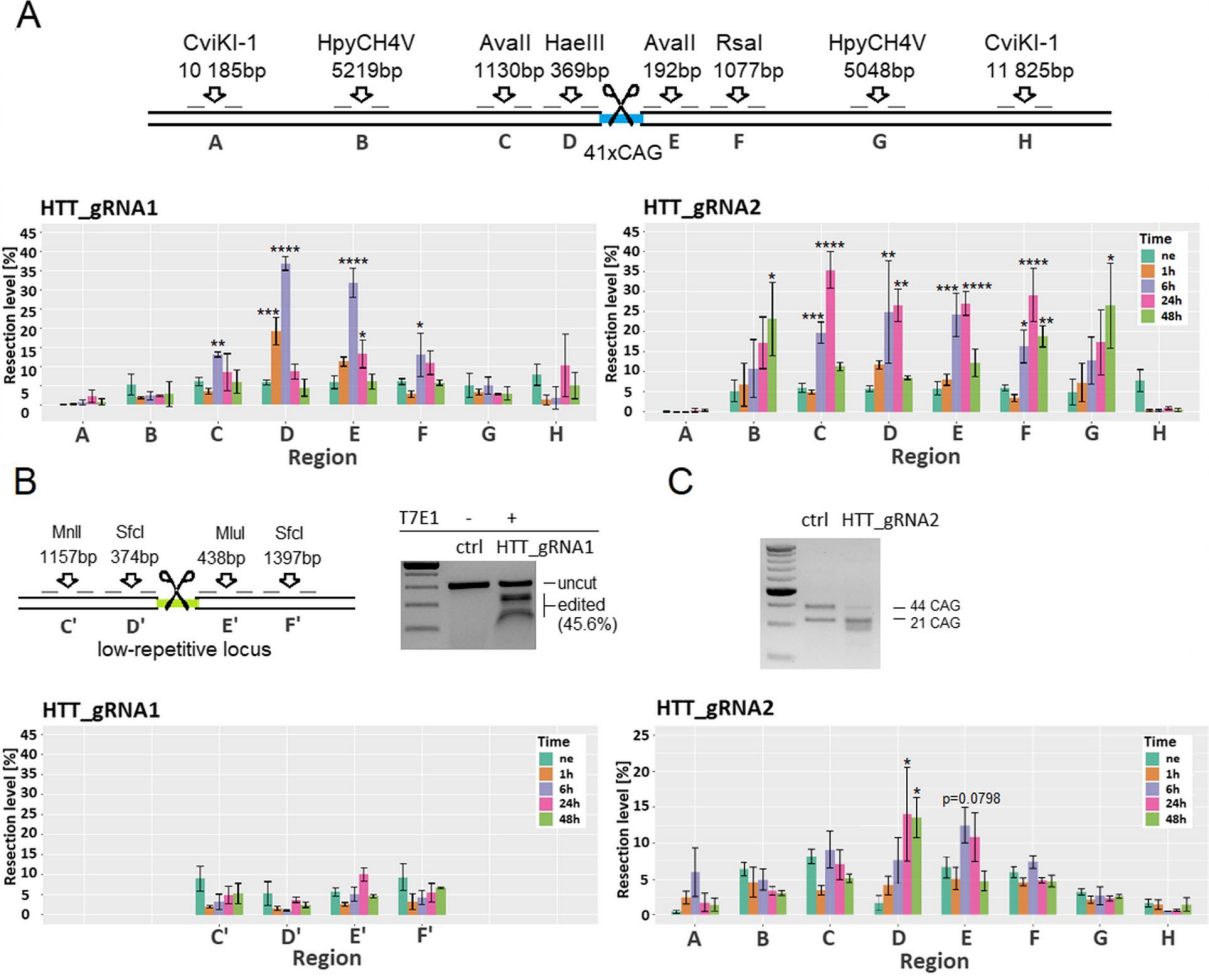
The level and range of DNA end resection after DSB induction. **A** Enzymes and restriction sites, with their distances from the CAG repeats in the *HTT* locus. The extent and level of resection at given time points after induction of DSB using Cas9-HTT_gRNA1 and Cas9-HTT_gRNA2 RNPs in HEK293T cells. **B** Restriction sites with their distances from the second target for HTT_gRNA1 in HEK293T cells (off-target locus containing 6 CAG repeats). The T7E1 assay confirmed the successful editing of the off-target locus after using HTT_gRNA1. **C** The extent and level of resection at the *HTT* locus after induction of DSBs using HTT_gRNA2 in GM04208 fibroblasts. The same regions and restriction enzymes were used as in HEK293T cells, with the exception of the D region, where the HpyAV enzyme was used due to the low efficiency of HaeIII. Each time point was compared to that of unedited cells (ne) using a one-way ANOVA test. The data shown represent the mean ± SD (*n* = 3), for details see Additional file 3. Asterisks indicate the level of statistical significance: *p ≤ 0.05, **p ≤ 0.01, ***p ≤ 0.001, ****p ≤ 0.0001

The large deletions of CAG repeats we observed suggest the involvement of mechanisms dependent on the resection of DNA ends. To investigate the extent of these resections and the effect of DSB localization, we performed qPCR on genomic DNA isolated from cells edited by RNP (Cas9-HTT_gRNA1 or Cas9-HTT_gRNA2) and digested by a set of restriction enzymes (Additional file 2, Table S1) [37]. Sites that underwent resection were not digested by endonucleases due to their requirement for double-stranded DNA and consequently could be efficiently amplified by qPCR with primers flanking the restriction site. Therefore, we were able to assess the resection level by comparing the Cq values obtained in the digested and nondigested fractions. Eight restriction sites were used: four located upstream (A, B, C, D) and four located downstream (E, F, G, H) of the CAG repeats in the *HTT* locus. The distances of these sites from the CAG tract were approximately 10,000 bp, 5000 bp, 1000 bp, and 200 bp (Fig. 2A). We observed that cleavage with HTT_gRNA1 led to efficient resection at sites proximal to the cutting site (D and E, approximately 35%) and less efficiently at sites 1000 bp from the DSB (C and F, > 15%) (Fig. 2A). No resection was detected in the distal regions (A, B, G, H). The maximum level of resection was observed 6 h post-transfection in the proximal regions (D and E), and the edited site was completely repaired 48 h post-transfection, as the level in each region was below the level of the nonedited control. In contrast, the maximum level of resection for HTT_gRNA2 was detected at 24 h post-transfection (approximately 35%) and in more distant regions compared to HTT_gRNA1 (C and F) (Fig. 2A). High resection levels were also observed in regions B and G (5000 bp from the CAG tract), but in the outermost regions (A and H), no resection was detected. After 48 h, the edited site was still being repaired, with the highest level detected in distal regions (B and G). The longer resection time observed for HTT_gRNA2 than for HTT_gRNA1 may be due to recutting of the edited template. These results are consistent with the outcomes from capillary electrophoresis of PCR products generated from DNA isolated from cells at several time points after transfection with RNP (Additional file 2, Fig. S1). No shortening of the sequence was detected in the 4 h after transfection with Cas9-HTT_gRNA1, as during this time, the resections were in progress. Interestingly, the shortening of the sequence 4 h after transfection with Cas9-HTT_gRNA2 was visible, although the resections were ongoing. To rule out the possibility that HEK293T cells are generally characterized by extensive resections, we performed a similar analysis for the second target *locus* (off-target) of HTT_gRNA1 (gRNA1_OT; chr15:47,717,511–47,717,530; hg38). Similarly, we selected four regions located approximately 1000 bp and 300 bp upstream and downstream of the edited site, respectively (Fig. 2B). We showed that there was no resection in this region, although it was efficiently edited (Fig. 2B). In addition, we performed DNA resection analysis in human HD fibroblasts containing 21/44 CAG repeats at the *HTT* locus. After using Cas9-HTT_gRNA2, the level of resection and extent of resection were much lower than those in the HEK293T-41CAG model (Fig. 2C). Statistically significant resections were observed only for the most proximal regions (D and E). This may be explained by the presence of a second allele with a shorter CAG repeat tract that is not efficiently edited (Fig. 2C) and/or cell type-specific effects.

### MRE11 and FEN1 enrichment in regions flanking CAG repeats

**Fig. 3:**
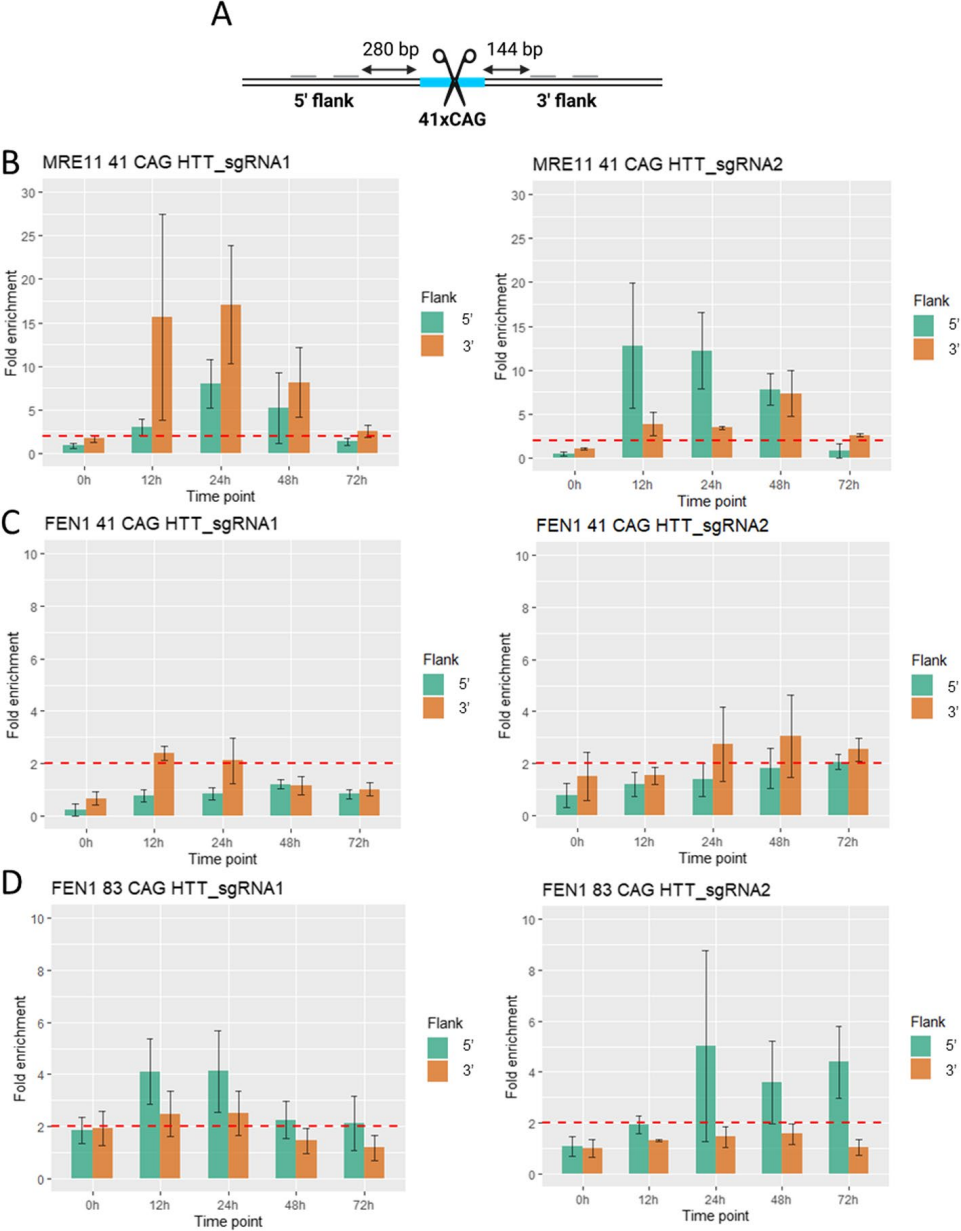
ChIP-qPCR analysis of the temporal distribution of MRE11 and FEN1 enrichment at the *HTT* locus after DSB induction. **A** The arrangement of qPCR primers relative to the CAG repeat tract. **B** The enrichment of the MRE11 protein at given time points determined by ChIP-qPCR after the induction of DSBs in the *HTT* locus with 41 CAG repeats using the HTT_sgRNA1 and HTT_sgRNA2 expressed from the PX458 plasmid. **C** The enrichment of FEN1 protein at given time points determined by ChIP-qPCR after the induction of DSBs in the *HTT* locus with 41 CAG or 83 CAG repeats (**D**) using HTT_sgRNA1 and HTT_sgRNA2 expressed from the PX458 plasmid. The data shown represent the mean ± SE (*n* = 3). A twofold increase in value compared to that in the control region was considered significant (marked with a red line). No significant differences were detected between protein enrichment at the 5′ and 3′ flanks or between time points according to the Kruskal–Wallis test and Wilcoxon rank sum test. The individual data values are provided in Additional file 3

MRE11, a component of the MRN complex, initiates the processing of DSBs during DNA repair. Its 3′ to 5′ exo- and endonuclease activities are responsible for inducing resection at the cleaved site. It has been demonstrated in a *Saccharomyces cerevisiae* model that the Mre11-Rad50 complex together with Sae2 is involved in DNA end processing at the TALEN-induced DSB site, leading to Rad52-mediated repeat contractions [7]. To determine whether MRE11 is involved in the initial resection of DNA ends leading to CAG repeat shortening, we assessed its enrichment in regions flanking the CAG repeats using ChIP-qPCR (Fig. 3A). We observed that cleavage with both guide RNAs led to high enrichment of the MRE11 protein (Fig. 3B), with a tendency toward asymmetry (not statistically significant). After cleavage with HTT_sgRNA1, MRE11 enrichment was very pronounced, especially at the 3′ flanking region (15-, 17-, and eightfold increases at 12, 24, and 48 h, respectively). In contrast, after cleavage with HTT_sgRNA2, amplification of the 5′ flanking region resulted in high and consistent enrichment (15-, 12-, and eightfold increases at 12, 24, and 48 h, respectively). FEN1 is an endonuclease responsible for flap structure removal during DNA repair and replication. In addition, flaps generated within repetitive sequences can fold into secondary structures that can be processed by FEN1 [38, 39]. Therefore, we analyzed FEN1 enrichment at regions flanking CAG repeats using ChIP-qPCR to determine whether FEN1 is involved in CAG repeat shortening. Additionally, we tested FEN1 enrichment in cells with different CAG repeat tract lengths (41 CAG and 83 CAG), as we hypothesized that the formation of structures removed by FEN1 would increase during DSB repair in longer repeat tracts. In cells with 41 CAG repeats, the FEN1 protein was slightly enriched in the 3′ flanking region after DSBs were induced with either guide RNAs (Fig. 3C). However, the distribution of enrichment varied over time depending on the location of the DNA break. In the case of HTT_sgRNA1, we observed a twofold increase at 12 and 24 h, while in the case of HTT_sgRNA2, an approximately threefold enrichment occurred at 24 and 48 h. Enrichment of the FEN1 protein after inducing DSBs within the 83 CAG repeat tract was greater and more pronounced for the 5′ flank (Fig. 3D). We observed a fourfold increase at 12 and 24 h for HTT_sgRNA1 and a fivefold, threefold, and fourfold increase at 24, 48, and 72 h, respectively, for HTT_sgRNA2. Interestingly, the temporal distribution of FEN1 enrichment after DSB induction remained the same for cells with 41 and 83 CAG repeats, implying that the kinetics of this process are independent of the CAG tract length.

### Searching for pathways involved in CAG repeat shortening by inhibiting key proteins involved in DSB repair

**Fig. 4:**
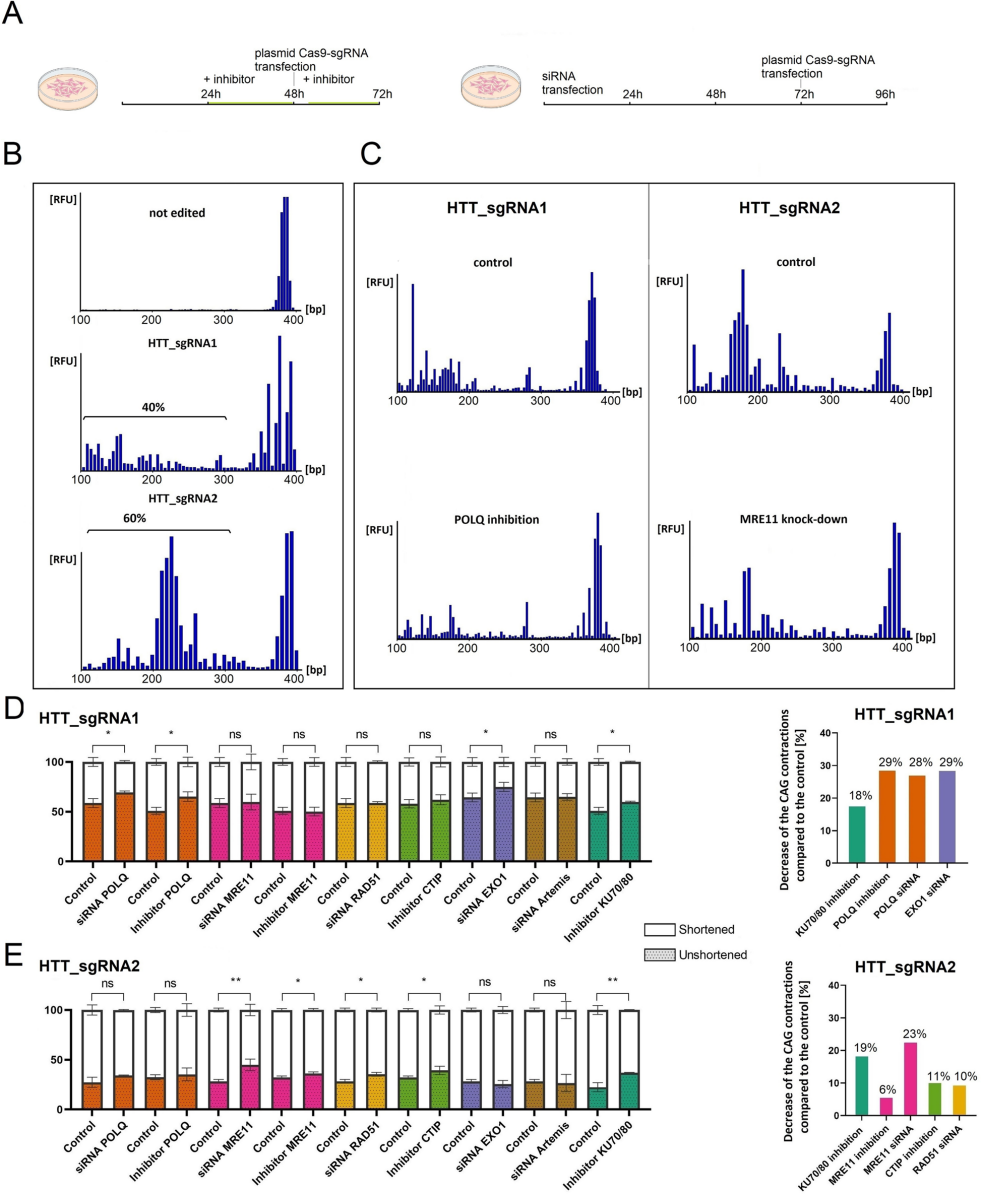
Effects of inhibiting selected DNA repair pathways with chemical inhibitors and siRNAs on CAG repeat shortening. **A** Workflow of the experiment. **B** Capillary electrophoresis of the PCR products after *HTT* locus editing using plasmid expressing HTT_sgRNA1 and HTT_sgRNA2. The percentage of CAG repeat contractions is shown. **C** Comparison of peak heights corresponding to edited (contracted) and unedited products in the control and treated samples. Note that these results should not be compared with the results from Fig. 1, since in this case each bar represents the amount of products of a given length (regardless of their sequence). **D**–**E** Percentages of shortened and full-length sequences obtained after the inhibition of individual proteins with chemical inhibitors or siRNA and DSB induction with HTT_sgRNA1 and HTT_sgRNA2, respectively. On the right, the percentage of reduction in the number of shortened products due to silencing of individual DNA repair proteins calculated from statistically significant results from the left. The data shown represent the mean ± SD (*n* = 3), for details see Additional file 3. The statistical analysis was performed using an unpaired *t*-test, where (*) indicates *p* < 0.05 and (**) indicates *p* < 0.01

To gain deeper insight into the mechanisms underlying CAG repeat shortening, we inhibited proteins involved in different DNA repair pathways with chemical inhibitors or siRNAs (Fig. 4A). We measured the ratio of signals from capillary electrophoresis (CE) peaks corresponding to full-length vs. edited (shortened) products. After the induction of DSBs with HTT_sgRNA1 and HTT_sgRNA2, peaks corresponding to shortened PCR products constituted approximately 40% and 60% of all signals, respectively (Fig. 4B). We used chemical inhibitors of 4 proteins (MRE11, CTIP, POLθ, KU70/80) and siRNAs to downregulate 5 genes (*MRE11*, *POLQ*, *EXO1*, *RAD51*, and *Artemis/DCLRE1C*) (Additional file 2, Table S2). Then, we compared the ratio of shortened vs. unchanged products in treated and control samples (Fig. 4C). Efficient inhibition of gene expression by siRNA was confirmed by western blotting and RT-qPCR (Additional file 2, Fig. S2). Inhibition and knockdown of the *POLQ* gene encoding POLθ resulted in an ~ 30% decrease in shortened products compared to those of the controls after DSB induction with HTT_sgRNA1 (Fig. 4D). Similarly, KU70/80 inhibition and *EXO1* knockdown decreased the levels of the truncated products by 18% and 29%, respectively. MRE11, CTIP, and RAD51 inhibition did not induce any changes in the ratio of shortened vs. full-length products compared to the control. Different proteins (except KU70/80) were determined to be significantly important in the CAG repeat contraction process after DSB induction with HTT_sgRNA2. The inhibition and gene knockdown of *MRE11* resulted in 6% and 23%, respectively, decreases in shortened products (Fig. 4E). KU70/80, CTIP, and RAD51 inhibition or gene knockdown resulted in decreases of 19%, 11%, and 10%, respectively, in the shortened products. Knockdown of *Artemis/DCLRE1C* had no effect on the proportion of the shortened products. It is worth noting, that in some cases, we observed differences between the patterns of peaks in CE for treated samples and controls, which was not reflected in the ratio of shortened to full-length products. For example, although MRE11 inhibition and silencing did not affect the percentage of truncated products, it changed the peak pattern (data not shown). These results indicate that the repair of DSBs at the *HTT* locus is complex and may involve different proteins depending on the location of the DSB and the sequence context.

### Detection of proteins interacting with the CAG tract during DNA break repair

To identify proteins involved in the repair of DNA spanning the CAG tract, we carried out engineered DNA-binding molecule-mediated chromatin immunoprecipitation (enChIP) followed by mass spectrometry (MS) (enChIP-MS) [40]. We transduced HEK293T-41CAG cells with a lentiviral vector coexpressing 3xFLAG-tagged Cas9 devoid of nuclease activity (dCas9-FLAG) and sgRNA targeting the flanking region upstream of the CAG tract in the *HTT* gene. After confirming the constitutive expression of dCas9-FLAG and the efficiency of the pull-down (Additional file 2, Fig. S3), DSBs were introduced using HTT_sgRNA1- or HTT_sgRNA2-expressing plasmids, and then complexes containing FLAG-dCas9 were immunoprecipitated and subjected to mass spectrometry for protein identification. The unedited cells were used as a control. Five replicates of every experimental variant were used.

A total of 402 and 404 proteins were identified as being differentially abundant (*q* value < 0.05, |fold change|≥ 1.5) in comparison to the noncleaved controls for HTT_sgRNA1 and HTT_sgRNA2, respectively (Additional file 2, Figs. S4 and S5). A STRING [41] network revealed that the identified proteins clustered into two relatively tight groups (translation and mRNA processing) and a looser cluster of proteins involved in chromatin organization and DNA repair. The levels of a total of 278 proteins were increased, and the levels of 124 proteins were decreased in the case of HTT_sgRNA1. The ratio was 275 to 129 for HTT_sgRNA2. Among the proteins that increased in level, the translation and DNA repair category was enriched, whereas the chromatin organization category was enriched among the proteins that decreased in level. Proteins related to mRNA processing were present in both the increased and decreased groups.

**Fig. 5:**
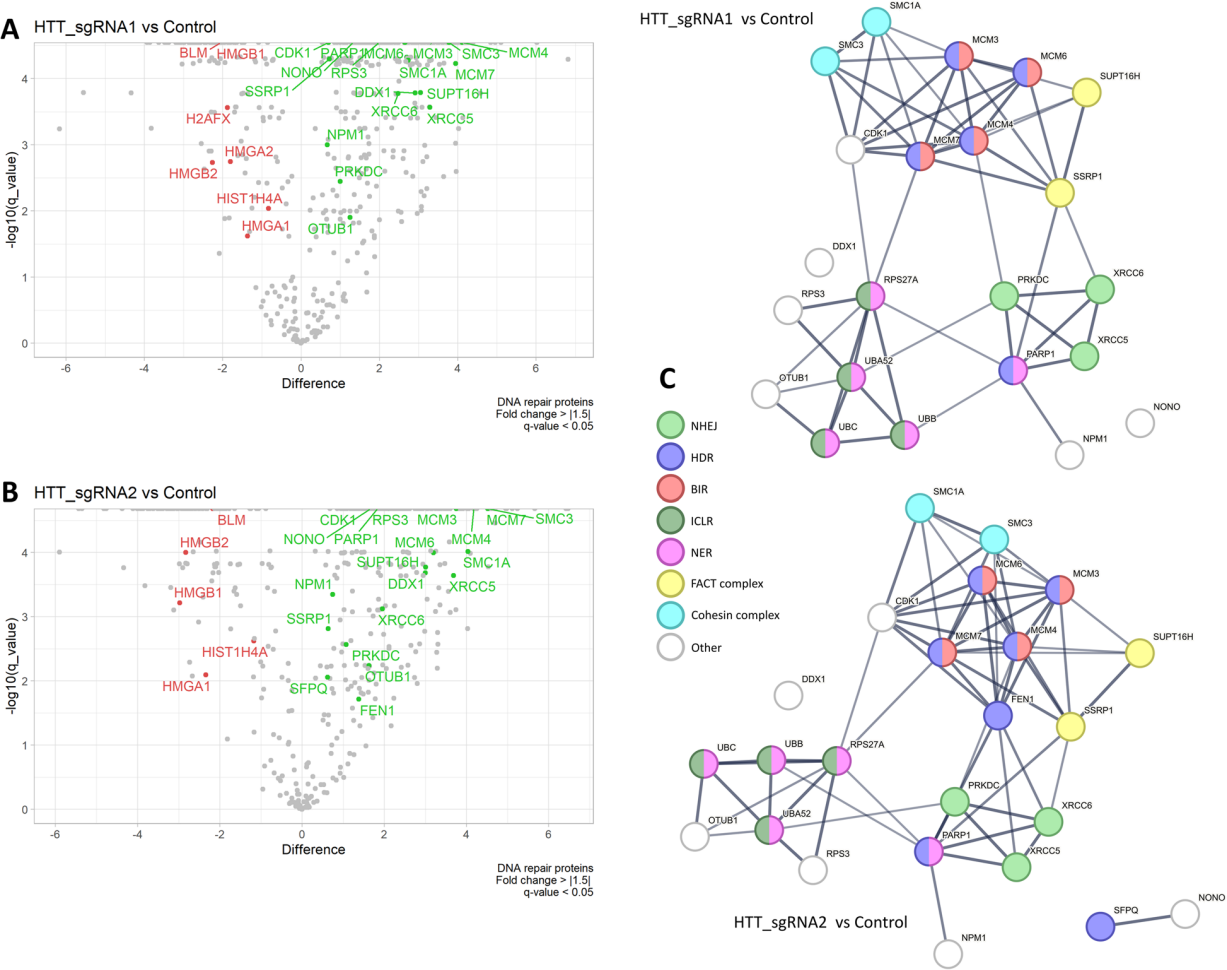
Analysis of proteins involved in DNA break repair within the CAG repeat region using enChIP-MS. **A** Volcano plot showing the distribution of the proteins identified by enChIP-MS analysis after DSB induction using HTT_sgRNA1. The “difference” parameter is the difference between the mean of log10 (protein spectrum intensity) of the experimental groups. The proteins enriched in the gene ontology category of DNA repair and characterized by a |fold change|≥ 1.5 and *q* value < 0.05 are marked in red (decreased levels) or green (increased levels). **B** Volcano plot showing the data obtained from the enChIP-MS analysis after DSB induction using HTT_sgRNA2. **C** STRING networks of the proteins that were enriched in the gene ontology category of DNA repair divided into subcategories. The networks were generated for data obtained after DSB induction using HTT_sgRNA1 (upper network) and HTT_sgRNA2 (lower network). The experiments were conducted with 5 biological replicates

The proteins that were enriched in the category of DNA repair were further analyzed (22 genes for HTT_sgRNA1 and 24 genes for HTT_sgRNA2, Fig. 5A–B). Figure 5C shows the STRING network of the identified DNA repair-related proteins further divided into categories of DNA repair pathways. We detected proteins involved in the regulation and execution of DNA repair related to DSB repair as well as to SSB repair. We detected the presence of the KU complex: XRCC5 (fold changes of 9.8 and 12.9 for HTT_sgRNA1 and HTT_sgRNA2, respectively), XRCC6 (fold changes of 5.6 and 3.9 for HTT_sgRNA1 and HTT_sgRNA2, respectively), and the PRKDC protein (a fold change of 2), which are known to be involved in NHEJ repair. Additionally, the PARP1 protein, which is considered a key player in the initiation of DNA repair, was detected (2.9-fold and 3.6-fold increase in the case of HTT_sgRNA1 and HTT_sgRNA2, respectively). We observed enrichment of the components of the MCM2-7 complex (MCM3, MCM4, MCM6, MCM7) involved in BIR and HDR. Ubiquitins related to the NER and ICLR pathways were also enriched (UBB, UBA52, UBC, and RPS27A: fold changes of the entire group: 6.3 and 4.2 for HTT_sgRNA1 and HTT_sgRNA2, respectively). We also identified both components of the histone chaperone FACT (SUPT16H, SSRP1, fold changes ~ 8), a central component of the condensin complex (SMC2, fold changes of 5.5 and 10.5, respectively) and two cohesins (SMC1A, SMC3, fold changes of 6.7 and 16.4 for HTT_sgRNA1 and HTT_sgRNA2, respectively). Interestingly, in the case of HTT_sgRNA2, we also detected the nuclease FEN1 (2.6-fold increase). The HTT_sgRNA2 variant was also enriched with the SFPQ protein (1.5-fold increase), which may be involved in HDR. The set of proteins significantly depleted in the DNA repair group included BLM, HMGA1, HMGA2, HMGB1, HMGB2, and HIST1H4A. Outside of the DNA repair category, we found other proteins of potential interest exhibiting level changes, such as increased levels of exportins (CSE1L and XPO1), components of the nuclear pore (NUP205 and NUP93), or decreased levels of DNA topoisomerase (TOP1).

EnChIP analysis revealed enrichment of a significant number of helicases involved in the process of DNA:RNA hybrid unwinding (e.g., DDX18, DDX1, DHX9, DDX5, and DDX21). DNA:RNA hybrids, particularly R-loops, occur naturally as a result of transcription, DNA replication, and DNA repair [42]. To verify whether DNA:RNA hybrids are created within the *HTT* locus during DNA repair, we performed DRIP-qPCR analysis after inducing DSBs with HTT_sgRNA1 and HTT_sgRNA2 (Additional file 2, Fig. S6A). The results showed that DNA:RNA hybrids were 2 times more abundant at the flanking regions of the CAG repeat tract after cleavage with HTT_sgRNA2 than at the negative control locus (ZNF554). After inducing DSBs with HTT_sgRNA1, no significant enrichment of DNA:RNA hybrids was detected at the *HTT* locus. However, it should be noted that in comparison to the previously published DNA:RNA hybrid-rich loci for HEK293T cells (LOC440704 and ING3 [43]), the enrichment in the *HTT* locus is noticeably lower. This may be due to the limitations caused by the efficiency of cell editing or the reduced stability of the hybrids at the studied locus. To further verify the presence of DNA:RNA hybrids during DSB repair within the *HTT* locus after inducing breaks with HTT_gRNA2, we performed immunofluorescence analysis using anti-DNA:RNA hybrids and anti-γH2AX antibodies (Additional file 2, Fig. S6B). The colocalization of the DNA:RNA hybrids and γH2AX histone was not significant. However, signals from the DNA:RNA hybrids in relation to γH2AX were higher in the HEK293T-41CAG cells than in the control cells after the induction of DSBs with HTT_gRNA2 (Additional file 2, Fig. S6C).

## Discussion

Previous studies that were conducted mainly on yeast models and in vitro systems have identified potential proteins and pathways associated with the shortening of CAG/CTG sequences following DSB induction. However, it remains unclear whether these processes are conserved in human cells. In this study, we demonstrated the phenomenon of tract shortening in an endogenous model of the human *HTT* gene, revealing that the process is highly complex and dependent on the site of the DSB. The analysis of the sequences of the final products of repair suggested that different mechanisms may act simultaneously on the substrate to produce various products. As expected, repair is initiated by the recruitment of NHEJ factors, but then the resection of DNA ends directs the process toward pathways involving the use of microhomology. We observed extensive DNA end resection in HEK293T-41CAG cells, especially when DSBs occurred within the repetitive tract. To date, the resection process has only been studied in yeast. Mosbach et al. observed resections reaching ~ 1900 bp upstream and ~ 2900 bp downstream from the CTG repeats [7]. In contrast, we did not observe asymmetry of the resection after HTT_gRNA1 or HTT_gRNA2 DSB induction. Importantly, our results suggest that MRE11 plays a role in initiating the resection process, as evidenced by the involvement of this protein regardless of the gRNA used. Interestingly, in the ChIP experiments, we observed an asymmetry in MRE11 enrichment (3′ flank for HTT_sgRNA1 and 5′ flank for HTT_sgRNA2), which could be explained by the mechanism of action of the CRISPR-Cas9 system. Following cleavage, Cas9 remains tightly bound to both DNA ends and releases the 3′-nontarget strand first [44]. The location of the PAM site determines which strand will be released first. In the case of HTT_sgRNA1, it is the CTG strand (downstream of the cleavage site), while in the case of HTT_sgRNA2, it is the CAG strand (upstream of the cleavage site). Therefore, initial processing by MRE11 may be asymmetric and dependent on the PAM location. Additionally, the sequence surrounding the Cas9 cleavage site is important because it may serve as an anchor for MH-dependent repair.

**Fig. 6:**
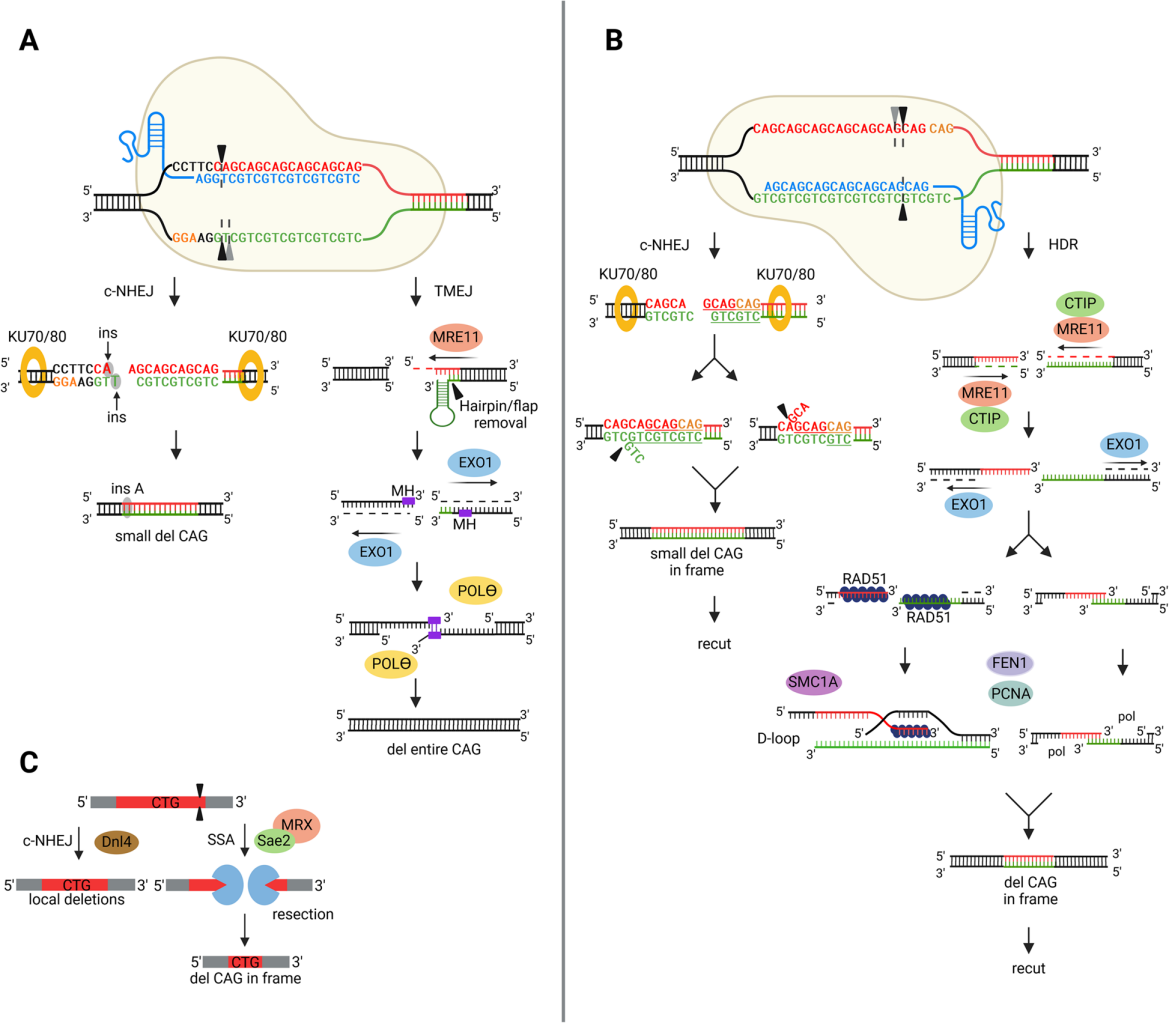
Model showing the complex repair process of double-strand breaks (DSBs) within the CAG trinucleotide repeat tract. **A** After inducing DSBs with CRISPR-Cas9 at the beginning of the CAG repeat tract (HTT_gRNA1), repair is possible by either canonical NHEJ (c-NHEJ) or theta-mediated end joining (TMEJ). Staggered cut processing through c-NHEJ leads to repair products with small CAG repeat deletions and an insertion of a single adenine 4 nucleotides from the cleavage site. TMEJ leads to a complete deletion of the CAG repeat tract. Resections performed by MRE11 (and EXO1) expose microhomologous regions (MH), which are utilized by polymerase theta to tether two single-stranded DNA ends, followed by gap filling. **B** After inducing DSBs within the CAG repeats (HTT_gRNA2), two repair pathways can be activated: c-NHEJ and HDR. Rapid repair of staggered cuts by c-NHEJ results in small in-frame CAG repeat deletions. In this way, the target site for CRISPR-Cas9-HTT_gRNA2 was reconstructed, and recutting was possible. HDR requires extensive resections performed by MRE11-CTIP and EXO1. The resulting single-stranded DNA fragments with homologous sequences anneal. This process may be dependent on the RAD51 protein and D-loop formation and may be additionally facilitated by the cohesin SMC1A. Alternatively, after strand assembly, the resulting gaps are filled in by a polymerase in cooperation with the PCNA protein. FEN1 removes flap structures during the repair process. HDR also causes CAG repeat deletion in-frame, allowing for recut. **C** A scheme for the repair of DSBs at the repeat site in the yeast system based on the mechanism proposed by the Richard’s group [7, 8]. DSBs can be repaired by NHEJ, which leads to small contractions of the CAG tract, or by SSA, which results in large contractions of the CAG tract

In light of our experimental findings, we propose a model explaining the mechanism underlying CAG/CTG tract shortening following Cas9-mediated DSB induction in HEK293T-41CAG cells. First, upon cleavage by Cas9-gRNA1, a staggered cut is generated, accompanied by the presence of NHEJ factors at the cleavage site. Intriguingly, we observed an A insertion at the site of cleavage in accordance with the − 4 rule [34, 45, 46], which supports the involvement of NHEJ in the process of DSB repair (Fig. 6A). Products containing insA mutations typically exhibit small deletions in the CAG repeats, which can arise from DNA slippage during replication or repair [3]. Unrepaired DNA ends, which are not resolved through classical NHEJ, can enter an alternative repair pathway involving MRE11-dependent short-range resection. The single-stranded CTG strand can adopt a flap or hairpin structure [3], which can be processed by endo- or exonucleases such as MRE11 and FEN1 [47]. This observation aligns with previous findings from the Richard’s group in studies conducted on yeast that demonstrated the involvement of the Mre11-Rad50-Sae2 complex on the side of the break containing the repetitive tract after asymmetrical cleavage [7, 8]. Then, MRE11 recruits the EXO1 protein, thereby facilitating long-range 5′-3′ resection. Aside from the observed resection events, the involvement of EXO1 is supported by the findings of the siRNA experiments. Finally, our data suggest the potential involvement of POLθ-mediated end joining, resulting in deletion of the entire CAG repeat tract. Short microhomologous sequences located within the flanks are used for anchoring DNA strands, and POLθ fills the gaps. DNA end resection and the presence of microhomologies imply that TMEJ may be the underlying mechanism. This notion is supported by the results of experiments involving POLθ chemical inhibitor and siRNA. Notably, even a 50% reduction in *POLQ* expression resulted in a significant decrease in products with delCAG. Moreover, we observed characteristic sequence mutational signatures of TMEJ, including templated insertions (Additional file 1) [48]. This finding is particularly intriguing considering that POLθ is not expressed in *Saccharomyces cerevisiae* [49], highlighting the importance of investigating these mechanisms in human cells.

In the case of HTT_gRNA2-guided cleavage, the mechanism underlying tract shortening differs considerably, which is particularly evident in the sequence of edited products. Remarkably, the majority of these products (~ 90%) exhibit in-frame deletions of CAG repeats. Furthermore, products with 4–10 repeats are predominant, which aligns with the findings reported in a previous study in a yeast model [7]. If, as with the use of gRNA1, Cas9-gRNA2 generated staggered cuts, the simplest way to repair the DSB would be to align the strands using short regions of microhomology with subsequent removal of small flaps by an endo- or exonuclease (Fig. 6B). This repair, probably NHEJ, results in small deletions of CAG repeats. The fact that HTT_gRNA2 binds entirely inside the repetitive tract makes subsequent recutting events possible, raising the possibility of an iterative nature of the repair process, a phenomenon suggested previously by Mosbach et al. [7]. A prolonged repair process may induce DNA resection, resulting in the release of DNA strands composed of CAG and CTG repeats of varying lengths. Realignment of these strands and subsequent filling of the gaps by polymerase may result in shortening of the CAG repeat tract. Our data indicate that FEN1, a structure-specific endonuclease, may participate in repair and replication processes by removing flap structures. According to previous reports, the activity of the FEN1 protein is important for the instability of the CAG repeat tract in the *HTT* gene during long-patch-BER [50–53] and MMEJ [54]. Long DNA resections detected after DSB induction with Cas9-gRNA2 are characteristic of HDR and SSA. In our experiments where *RAD51* (a key HDR protein) was effectively knocked down, the number of CAG deletions decreased, suggesting the involvement of this protein in CAG contractions. In cooperation with other factors, RAD51 forms filaments on the 3′ ssDNA tail and invades donor dsDNA [55–57], creating a D-loop, followed by DNA synthesis performed by replicative DNA polymerases [55]. enChIP experiments revealed enrichment of proliferating cell nuclear antigen (PCNA), the enhancer of the processivity of replicative DNA polymerases [56]. FEN1 is known to interact with PCNA, together these proteins participate in the processes of DNA replication and repair, especially during Okazaki fragment maturation and gap-filling synthesis [58]. The enrichment of PCNA in the HTT_gRNA2 samples was 60% greater than that in the HTT_gRNA1 samples. Similarly, enrichment of SMC1A (part of the recombination protein complex (RC-1) involved in DNA repair via recombination) [57] was almost 2.5-fold greater in the HTT_gRNA2 samples than in the gRNA1 samples. This evidence supports the involvement of the recombination-dependent pathway in creating in-frame CAG deletions after DSB induction with HTT_gRNA2. The same product may also be formed by the SSA pathway, as suggested in many previous studies.

The outcomes from unbiased proteomics conducted using enChIP support some previous findings. For instance, the association of the elements of the nuclear pore complex with the repetitive tract aligns with the model proposing that the relocalization of CAG trinucleotide repeats to the nuclear pore complex occurs during DNA repair [59]. We also identified the FACT complex which participates in Cas9 displacement [60]. Furthermore, proteomics analysis has yielded novel insights revealing the participation of helicases in the repair machinery at the site of CAG repeats. Admittedly, our study faced certain limitations in terms of identifying proteins that could support the proposed hypothesis. One of the limitations is the lack of cross-linking during the enChIP assay, which may result in the loss of certain proteins during the experimental process. Additionally, proteins such as POLθ are known to have low expression levels in cells, further complicating their detection. Moreover, the repair process itself is dynamic, and the timing of the experimental assays is crucial for accurately capturing specific events. Technical constraints also require the use of plasmids alongside RNPs in ChIP experiments, which can influence repair kinetics. Considering these limitations, further investigations with improved techniques and methodologies are necessary to gain a more comprehensive understanding of the proteins involved and the precise dynamics of the repair process. Importantly, our results demonstrate that the mechanism of Cas9 action itself seems to influence the repair process. For example, after using HTT_gRNA1 we observed insA events, in accordance with the − 4 rule.

In perspective, there are several promising directions for further exploration. In the next steps, investigating the effects of heterozygosity could provide valuable insights into the possible role of HDR. The lower resection efficiency in HD fibroblasts than in HEK293T-41CAG cells may suggest the involvement of different DNA repair mechanisms in these two models. While these cell type-specific effects are not unexpected, they require further investigation. Additionally, utilizing nondividing neuronal cells can offer a relevant cellular context for studying CAG tract shortening, as neurodegenerative disorders such as HD primarily affect neurons. Furthermore, efforts should be made to gain better control over the tract shortening process. Exploring strategies to modulate and regulate repair outcomes, such as controlling the cell cycle, or using inhibitors of selected DNA repair pathways, could enhance our ability to precisely control and manipulate CAG tract shortening.

## Conclusions

Here, we identified key mechanisms responsible for CAG repeat shortening following DSB induction with Cas9 endonuclease in human cells at the endogenous *HTT* locus. We showed that the endonuclease cleavage site and the DSB flanking sequence play a key role in the selection of the DNA repair mechanism, resulting in different editing outcomes. The shortening of CAG repeats results from both NHEJ pathway activity and homology-mediated repair, which involves DNA end resection. Although our findings generally confirm the results from the yeast model, they also demonstrated a previously unknown role of TMEJ in the repair of CAG repeats. This study expands our knowledge of genome editing and DNA repair, and is important for the development of CRISPR-based therapeutic approaches for incurable repeat expansion diseases.

## Methods

### The CRISPR-Cas9 system

The guide RNAs targeting exon 1 of the *HTT* gene (HTT_sg1, HTT_sg2, HTT_sg3, and HTT_sg4) have previously been described and validated [31, 32]. Depending on the experiment, we used a plasmid-based CRISPR-Cas9 system (PX458) or RNP complexes. The RNP complex was prepared according to the IDT Alt-R CRISPR-Cas9 System instructions. Briefly, the crRNA and tracrRNA oligos were mixed in equimolar concentrations to a final duplex concentration of 44 μM (HTT_gRNA1, HTT_gRNA2). The mixture was heated at 95 °C for 5 min and incubated at room temperature for 20 min. Then, the guide complex was mixed with the SpCas9 enzyme (VBCF Protein Technologies facility http://www.vbcf.ac.at) at a ratio of 22 pmol to 18 pmol. The mixture was incubated at room temperature for 20 min. The sequences of specific oligodeoxynucleotides are listed in Additional file 2, Table S3.

### Cell culture and transfection

We used previously generated cell lines: HEK293T-41CAG cells (41/41 CAG in the *HTT* gene) and HEK293T-83CAG cells (83/83 CAG in the *HTT* gene) [29]. The cells were grown in Dulbecco’s modified Eagle’s medium (Lonza; Basel, Switzerland) supplemented with 10% fetal bovine serum (Sigma-Aldrich), antibiotics (Sigma-Aldrich), and L-glutamine (Sigma-Aldrich). Fibroblasts from HD patient (GM04208) were obtained from Coriell Cell Repositories (Camden, NJ, USA) and grown in minimal essential medium (MEM) (Sigma-Aldrich) supplemented with 10% fetal bovine serum and antibiotics. The cell lines were routinely checked for mycoplasma and tested negative using a PCR-based method.

Transfections were performed with the NeonTM Transfection System (Invitrogen, Carlsbad, CA, USA). We used 10 μl tips and the following parameters: 1150 V, 20 ms, 2 pulses for HEK293T cells and 100 μl tips and 1350 V, 30 ms, 2 pulses for fibroblasts. As an alternative, cells were transfected with plasmids (described previously [31]) using the NeonTM Transfection System (Invitrogen). A total of 5 × 10^6^ cells in 10-cm tissue culture plates were transfected with 25 µg of PX458 plasmid containing cloned sgRNA using the electroporation method (1100 V, 20 ms, 2 pulses). Alternatively, the polyethylenimine (PEI) method was used. A total of 4 × 10^5^ cells were transfected with 2.5 µg of a plasmid complexed with 7.5 µl of PEI (1 mg/ml). The method was scaled up if necessary.

### DNA amplification and targeted deep sequencing

Genomic DNA was extracted using a Genomic DNA Isolation Kit (Norgen, Biotek Corp.) according to the manufacturer’s instructions and quantified using a spectrophotometer/fluorometer (DeNovix). The DNA obtained from cell cultures transfected with the RNP complex was amplified by nested PCR using Herculase II Fusion DNA Polymerase (Agilent). The first amplification was performed with primers IG36 and IG37 as follows: initial denaturation at 98 °C for 3 min; 35 cycles at 98 °C for 20 s, 61.1 °C for 20 s, and 72 °C for 20 s; and a final elongation at 72 °C for 3 min. PCR products were purified using the GeneJET PCR Purification Kit (Thermo Fisher), diluted 10 × and used as a template for the second amplification performed with primers IG30 and IG31 as follows: initial denaturation at 98 °C for 3 min; 30 cycles at 98 °C for 20 s, 56 °C for 20 s, and 72 °C for 12 s; and a final elongation at 72 °C. Sequences of specific primers are listed in Additional file 2, Table S1. The length of the uncleaved product was 242 bp. The PCR products were used for library generation and next-generation sequencing, which were performed by Novogene (UK). Sequencing libraries were generated using the NEBNext Ultra II DNA Library Prep Kit for Illumina (New England Biolabs, UK) following the manufactures’ protocol. Libraries were sequenced using the Illumina NovaSeq 6000 platform with the following settings: 250 bp paired-end reads and 1 million reads per sample. The obtained data were deposited in the Sequence Read Archive (SRA, accession number: PRJNA1006315) [61].

### Bioinformatic analysis

Analysis was performed by ideas4biology Ltd. Briefly, the overall quality of the data was tested with FastQC version 0.11.5. Adapters were removed using a standard adapter sequence set from the bbduk2 package. Reads with a quality below 5 were discarded. The reads were merged using the bbmerge script from the BBMap package. After merging, the reads were mapped against the *HTT* gene sequence using the BBMap global aligner. Mapping quality was assessed using qualimap v.2.2.2-dev. Aligned reads were filtered using the -F260 and -q 30 flags. Sequences were clustered using the cd-hit application [62]. Sequences with the same length and at least 95% sequence similarity were merged into clusters..bam files containing unique sets of reads were converted to pairwise alignments using the sam2pairwise program. To locate and display tandem repeats in DNA sequences, we used Tandem Repeats Finder v409. The recommended parameters were used except for the minimum alignment score, which was set to 10 to allow for shorter hits. The results were filtered for repeats containing only combinations of the letters C, A, and G. Reads for each “CAG” repeat length were counted and the ratio of the number of reads containing a given “CAG” length to all reads was calculated.

### Chromatin immunoprecipitation

A total of 5 × 10^6^ cells were transfected with 25 µg of PX458 plasmid containing HTT_sgRNA1 or HTT_sgRNA2 using the electroporation method described above. The cells were then collected at 5 time points (0 h, 12 h, 24 h, 48 h, and 72 h) in the following way. Formaldehyde (Thermo Scientific) at a final concentration of 1% was used to cross-link the cell chromatin. The reaction was stopped by the addition of glycine (BioShop) at a final concentration of 1.11 M. The cells were then collected by scraping in cold 1 × PBS supplemented with 1 mM PMSF. After centrifugation, the cells were lysed in RIPA buffer (50 mM Tris–HCl pH 8, 150 mM NaCl, 2 mM EDTA pH 8, 1% Triton X-100, 0.5% sodium deoxycholate, 0.2% SDS) supplemented with a protease inhibitor cocktail (PIC, BioShop) on ice. The chromatin was sonicated using a BioRuptor Pico sonicator (Diagenode; 5 cycles, 30 s ON, 30 s OFF, the average length of fragments was 400 bp). Finally, the lysates were centrifuged (12,500 × g, 4 °C, 5 min) and the supernatants were transferred to new tubes. Lysates were suspended in 710 µl of RIPA buffer with 7.5 µl of PIC and incubated with gentle mixing overnight at 4 °C with 20 µl of DynabeadsTM Protein G (Invitrogen) coated with an antibody against the analyzed protein (listed in Additional file 2, Table S2). Next, the beads were separated on a magnetic separator and washed 6 times with RIPA buffer and 1 time with 1 × TBS (Pierce). Elution was performed by incubating of the beads in elution buffer (50 mM Tris–HCl pH 8, 10 mM EDTA pH 8, 1% SDS) at 65 °C for 5 h. The eluate was transferred to a new tube containing 100 μl of TE buffer and then incubated with proteinase K (Diagenode) at 55 °C for 1 h. The DNA was purified with a GeneJET PCR Purification Kit (Thermo Scientific). For normalization of the chromatin concentration between samples, the input was prepared as described above, but incubation with magnetic beads was omitted.

### ChIP-qPCR

For input and eluate samples collected at 5 time points, qPCR was performed using a CFX Connect Real-Time PCR Detection System (Bio-Rad, Hercules, CA) and SsoAdvanced Universal SYBR Green Supermix (Bio-Rad). The 5′ and 3′ flanking regions of the CAG tract in the *HTT* gene were amplified under the following thermal cycling conditions: denaturation at 98 °C for 3 min followed by 50 cycles of denaturation at 95 °C for 15 s, annealing at 66.2 °C for 30 s and elongation at 72 °C for 30 s. As a reference gene, *β-actin* was amplified under the following thermal cycling conditions: denaturation at 95 °C for 30 s followed by 40 cycles of denaturation at 95 °C for 15 s and annealing at 60 °C for 30 s. Primer sequences are listed in Additional file 2, Table S3. The value of input recovery was determined on the basis of the average quantification cycle (Cq) for reactions carried out for the input and eluate. The ratio between the input recovery for the regions flanking the CAG tract in the *HTT* gene and that for the reference gene (*β-actin*) was used to calculate the fold enrichment.

### DNA:RNA immunoprecipitation

A total of 5 × 10^6^ cells were transfected with 25 µg of PX458 plasmid containing cloned HTT_sgRNA1 or HTT_sgRNA2 using electroporation. The cells were then collected after 12 h, and lysates were prepared as described above for ChIP. Lysates were digested with proteinase K (Norgen Biotek Corp.) and decrosslinked at 65 °C overnight. Later, DNA containing DNA:RNA hybrids was isolated by phenol:chloroform extraction and precipitated with isopropanol (1:1 v/v) with 3 M sodium acetate (1:10 v/v). Six micrograms of isolated DNA was left as an input and 6 µg of DNA was used for DRIP. The DNA was incubated in IP buffer (50 mM HEPES/KOH at pH 7.5; 0.14 M NaCl; 5 mM EDTA; 1% Triton X-100; 0.1% Na-deoxycholate) with 20 µl of DynabeadsTM Protein A (Invitrogen) coated with 10 µl of S9.6 antibody (Sigma Aldrich, listed in Additional file 2, Table S2) at 4 °C with gentle mixing overnight. Next, the beads were separated on a magnetic separator and washed once with IP buffer for 1 min at 4 °C, high salt buffer (50 mM HEPES/KOH pH 7.5, 0.5 M NaCl, 5 mM EDTA pH 8, 1% Triton X-100, 0.1% Na-deoxycholate) for 1 min at 4 °C, wash buffer (10 mM Tris–HCl pH 8, 0.25 M LiCl, 0.5% NP-40, 0.5% Na-deoxycholate, 1 mM EDTA pH 8) for 1 min at 4 °C and washed twice with TE buffer (50 mM Tris–HCl pH 8, 10 mM EDTA, 1% SDS) for 1 min at 4 °C. The beads were eluted by incubation in elution buffer (50 mM Tris–HCl pH 8, 10 mM EDTA pH 8, 1% SDS) at 65 °C for 45 min. The remaining antibodies were digested with proteinase K for 1 h at 55 °C. The DNA was then purified by phenol:chloroform extraction and precipitated with isopropanol with sodium acetate as described above.

### DRIP-qPCR

For eluate and 100-fold diluted input, qPCR was performed using a CFX Connect Real-Time PCR Detection System (Bio-Rad, Hercules, CA) and SsoAdvanced Universal SYBR Green Supermix (Bio-Rad). The 5′ and 3′ flanking regions of the CAG tract in the *HTT* gene as well as the positive control locus (LOC440704) and negative control locus (ZNF554) were amplified by denaturation at 98 °C for 3 min, followed by 40 cycles of denaturation at 95 °C for 15 s, annealing at 66.2 °C for 30 s, and elongation at 72 °C for 30 s. An additional positive locus (ING3) was amplified under the following thermal cycling conditions: denaturation at 95 °C for 30 s, followed by 40 cycles of denaturation at 95 °C for 15 s and annealing at 60 °C for 30 s. Primer sequences are listed in Additional file 2, Table S3. The value of input recovery was determined on the basis of the average Cq for reactions carried out for the input and eluate. The ratio between the input recovery for the regions flanking the CAG tract in the *HTT* gene or positive control locus and the negative control locus (*ZNF554*) was used to calculate the fold enrichment.

### Immunofluorescence

HEK293T, HEK293T-41CAG, and HEK293T-83CAG cells were seeded on 12-well plates with glass coverslips coated with Geltrex (Thermo Fisher) 3 h prior to transfection. Cells were transfected with RNP complexes containing HTT_gRNA1 or HTT_gRNA2 using Lipofectamine RNAiMAX (Invitrogen). After 3 h, the cells were fixed with 4% paraformaldehyde for 30 min at RT. The fixed samples were washed once with 1 × PBS. The cells were permeabilized with 0.5% Triton X-100 in 1 × PBS for 10 min and blocked in 1% BSA with 0.2% Triton X-100 in 1 × PBS for 1 h at RT. The samples were washed with 1 × PBS 3 times. Coverslips were incubated overnight at 4 °C with the primary antibodies anti-DNA:RNA hybrids and anti-γH2AX (listed in Additional file 2, Table S4) diluted in 1% BSA with 0.2% Triton X-100 in 1 × PBS. The next day, the coverslips were washed three times with 1 × PBS and incubated with anti-mouse and anti-rabbit antibodies labeled with Alexa488 and Alexa647, respectively (1:500, listed in Additional file 2, Table S2), for 1 h at RT. After rinsing, the coverslips were mounted onto glass with SlowFade Diamond Antifade Mountant with DAPI (Life Technologies). Images were captured with a Leica SP5 confocal microscope at 63 × magnification. Leica Application Suite X (3.3.3) was used to analyze colocalization. The most representative cell nuclei were selected for analysis. Fluorescence intensity was measured over the entire nucleus (at least 16 nuclei from cells of each cell type) and calculated as a ratio between signals from DNA:RNA hybrids and γH2AX histones. Colocalization analysis was determined based on Pearson’s coefficient values for the most intense spots in each of the examined cells, the color of which indicated the presence of signals for both DNA:RNA and γH2AX hybrids in the same locus (at least 70 spots total, from at least 15 cells of each cell type). The number of analyzed spots per nuclei varied between individual nuclei.

### Resection analysis

Cells were electroporated with RNP complexes containing HTT_gRNA1 or HTT_gRNA2. After electroporation, the cells were cultivated in complete DMEM supplemented with 20% FBS and harvested for DNA isolation at 1 h, 6 h, 24 h, and 48 h post-transfection. DNA was isolated using a Genomic DNA Isolation Kit (NORGEN Biotek) according to the manufacturer’s protocol. Each sample was divided into two parts: one was incubated overnight with restriction enzymes, and the other was incubated without restriction enzymes (see Additional file 2, Table S1). The efficiency of all restriction enzymes in a particular site was confirmed before conducting the experiment. In not edited samples, the resection level for each enzyme was below 10%. The resection level detected in not edited samples should be recognized as the cut-off level for a particular site.

After incubation, the DNA was subjected to qPCR with primers flanking the restriction sites in separate reactions for each pair of primers (see Additional file 2, Table S3). Each PCR was performed in three technical replicates, and Cq results were used to obtain the Cq mean and to calculate the level of resection for each time point and site from A to H using the formula presented in [37]. Each experiment was conducted with three biological replicates.

### Inhibition of DNA repair proteins

HEK293T-41CAG cells (3 × 10^5^ cells per well) were cultivated in 6-well plates in complete DMEM. One day after passage, the medium was changed to complete DMEM containing a particular inhibitor (see Additional file 2, Table S4). After 24 h of incubation, the cells were transfected with PEI-plasmid complexes (2.5 µg of PX458 plasmid and 7.5 µl of PEI per well). After 4 h, the medium was changed to DMEM containing a specific inhibitor at the same concentration as previously described. Twenty-four hours post-transfection, the cells were harvested and sorted on a FACSAria Fusion cell sorter (Becton Dickinson). DNA from GFP-positive cells was isolated using QuickExtract DNA Extraction Solution (Lucigen) according to the manufacturer’s protocol. The isolated DNA was subjected to PCR with the primers IG36 and IG37 primers (the forward primer labeled with fluorescein at the 5′ end). Q5 polymerase (NEB) was used for the amplification of the edited *HTT* locus as follows: initial denaturation at 98 °C for 3 min; 35 cycles at 98 °C for 10 s, 68 °C for 30 s, and 72 °C for 5 s; and a final elongation at 72 °C for 2 min. The PCR products were subsequently purified using the Gene JET PCR Purification Kit (Thermo Fisher) according to the manufacturer’s protocol.

### siRNA-mediated knockdown of DNA repair proteins

A total of 2 × 10^5^ HEK293T-41CAG cells per well were subjected to reverse transfection using a particular siRNA (see Additional file 2, Table S4). siRNA reverse transfection was performed as follows: 5 µl of Lipofectamine 2000 (Thermo Fisher) was incubated at room temperature with siRNA for 20 min in 500 µl of OptiMEM (Gibco) in each well of a 6-well plate. A total of 2 × 10^5^ cells in 2 ml of complete DMEM were added to the Lipofectamine-siRNA complexes. The final concentration of the siRNA ranged from 50 to 100 nM (Additional file 2, Table S2). Seventy-two hours post-transfection, the cells were transfected with PEI-plasmid complexes (2.5 µg of PX458 plasmid and 7.5 µl of PEI per well). Twenty-four hours post-transfection, the cells were harvested, sorted, and treated according to the methods described in the “Inhibition of DNA repair proteins” section. The experiment was conducted with three biological replicates.

### Analysis of the PCR products by capillary electrophoresis

Capillary electrophoresis was performed on an ABI Prism 3130xl (Applied Biosystems) with POP-7 polymer using a G5 filter. One microliter of sample was diluted in 9 µl Hi-Di™ formamide (Applied Biosystems) containing 0.25 µl of GeneScan™ 600 LIZ™ Size Standard. Before resolution, the samples were denatured at 95 °C for 5 min and cooled to 10 °C. The results were analyzed with Peak Scanner Software V1.0 (Applied Biosystems). Each peak was described by size, height, and area in BP, which indicates the peak area based on base pairs. To cut off the background for each sample, every peak in which the height parameter was less than 5% of the value of this parameter related to the highest peak in a particular sample was excluded from the calculation. Then, the areas in BP for each peak were added, as well as the areas in BP for peaks corresponding only to PCR products containing 41 CAG repeats, to calculate the percentages of shortened and full-length products. The mean percentage for treated and control samples was calculated using values from three biological replicates. Statistical significance was determined using an unpaired *t*-test.

### enChIP

#### Generation of HEK293T-dCas9-3xFLAG cells

gRNA targeting a site 208 bp upstream from the CAG tract was inserted into the 3xFLAG-dCas9-HA-2xNLS (Addgene) plasmid by digesting the plasmid with BsmBI (Esp3I) and ligating it to annealed gRNA oligos (see Additional file 2, Table S3). For lentivirus production, the plasmid was cotransfected with the packaging plasmids pPACKH1-GAG, pPACKH1-REV, and pVSVG (System Biosciences) into HEK293TN cells. The medium was collected on days 2 and 3, and the viral supernatants were passed through 0.45-μm filters and concentrated using PEGit Virus Precipitation Solution (System Biosciences). The lentiviral vectors were resuspended in Opti-MEM (GIBCO, Invitrogen, Carlsbad, CA). To generate the HEK293T-dCas9-3xFLAG transgenic cell line, cells were transduced with lentivirus in the presence of 8 μg/ml polybrene and selected using 1.5 μg/ml puromycin. Monoclonal transgenic lines were generated by transduction at a low cell density using a low multiplicity of infection (MOI < 1) and allowing cells that survived selection to form colonies before individual clones were isolated.

#### Protein extraction and immunoblotting

Total protein extracts were generated by lysing cells in RIPA buffer. A total of 30 μg of protein was diluted in sample buffer containing 2-mercaptoethanol and boiled for 5 min. The samples were separated on a 12% Tris–acetate SDS–polyacrylamide gel and transferred to a nitrocellulose membrane (Sigma-Aldrich). The membranes were blocked in 1 × TBS containing 0.1% Tween-20% and 5% nonfat dry milk for 2 h at room temperature. Primary and secondary antibodies were used in 1 × TBS/0.1% Tween 20 buffer containing 5% nonfat milk. Immunoreactions were detected using Western Bright Quantum HRP Substrate (Advansta, Menlo Park, CA).

#### enChIP-qPCR

The procedure was adapted with modifications from Fujita and Fujii [40]. Briefly, 5 × 10^6^ HEK293T-dCas9-3xFLAG cells in 10-cm tissue culture plates were transfected with 25 µg of PX458 plasmid using the PEI transfection method. PEI-treated cells were used as a control. After 24 h, the cells were washed 4 times with cold 1 × PBS and then collected by scraping in cold 1 × PBS containing 1 mM PMSF. After centrifugation, the cells were lysed in RIPA buffer with a protease inhibitor cocktail (BioShop) on ice, and the chromatin was sonicated using a BioRuptor Pico sonicator (Diagenode; 5 cycles, 30 s ON, 30 s OFF, the average length of fragments was 400 bp). Then, the cells were centrifuged (12,500 × g, 4 °C) and the supernatants were transferred to new tubes and incubated overnight at 4 °C with 15 µl of Anti-FLAG® M2 Magnetic Beads (Merck) with gentle mixing. Next, the beads were separated on a magnetic separator and washed 6 times with RIPA buffer and 6 times with 1 × TBS (Pierce). Elution was performed by incubating of the beads with 3xFLAG peptide (Sigma Aldrich) at 37 °C for 30 min. Then, the eluate was incubated with RNase at 37 °C for 1 h, and the DNA was purified with a GeneJET PCR Purification Kit (Thermo Scientific). Quantitative PCR was performed in a CFX Connect Real-Time PCR Detection System (Bio-Rad, Hercules, CA) using SsoAdvanced Universal SYBR Green Supermix (Bio-Rad) with *β-actin* as the reference gene under the following thermal cycling conditions: denaturation at 95 °C for 30 s followed by 40 cycles of denaturation at 95 °C for 15 s and annealing at 60 °C for 30 s. Primer sequences are listed in Additional file 2, Table S3.

#### enChIP-MS

The procedure was performed as described above until the final wash (six times with RIPA buffer and six times with 1 × TBS). Then, mass spectrometry was performed by the Mass Spectrometry Laboratory, IBB, PAS. The analysis was conducted in 5 replicates. First, the cysteines were reduced by a 1 h incubation with 20 mM tris (2-carboxyethyl)phosphine (TCEP) at 37 °C, followed by a 10 min incubation at room temperature with 50 mM methyl methanethiosulfonate (MMTS). Digestion was performed at 37 °C overnight with 1 µg of trypsin (Promega). After digestion, the peptides were eluted from the beads by using a magnet. The pulled aliquots were dried in a SpeedVac and resuspended in extraction buffer (0.1% TFA 2% acetonitrile) with sonication. The next step involved single-pot solid-phase-enhanced sample preparation (SP3). The magnetic bead mixes were prepared by combining equal amounts of Sera-Mag carboxyl hydrophilic and hydrophobic particles (09–981-121 and 09–981-123, GE Healthcare). The bead mixture was washed three times with MS-grade water and resuspended at a working concentration of 10 µg/µl. The bead mixture was then added to the samples and suspended in 100% acetonitrile, and this step was repeated 3 times. Pure peptides were eluted from the beads by using 2% acetonitrile in MS-grade water. Using a magnet, the peptide solution was separated from the beads. The peptide mixture was dried in a SpeedVac and resuspended in 80 µl of extraction buffer (0.1% TFA and 2% acetonitrile) via sonication. Twenty microliters of each sample were measured on an Orbitrap Exploris 480 mass spectrometer (Thermo Scientific) coupled to an Evosep One chromatograph (Evosep Biognosys). The samples were loaded onto disposable Evotips C18 trap columns (Evosep Biosystems) according to the manufacturer’s protocol with minor modifications as described previously [63]. Chromatography was carried out at a flow rate of 250 nL/min using an 88 min (15 samples per day) gradient on an EV1106 analytical column (Dr Maisch C18 AQ, 1.9 µm beads, 150 µm ID, 15 cm long, Evosep Biosystems). The resulting raw data were processed with MaxQuant software (version 2.1.1) to obtain the LFQ intensities and protein identifications using the *Homo sapiens* reference proteome from UniProt (one protein per gene) supplemented with the Cas9 protein sequence (20,595 sequences) with contaminants included. Further analysis was performed in the Perseus suite (version 1.6.15). Proteins with fewer than 3 valid values in both groups were not included in the statistical analysis. The data were log transformed, the missing values were imputed from a normal distribution (width 0.3, downshift 1.8), and a two-sample *t*-test was performed to estimate the statistical significance. The obtained data were deposited in the ProteomeXchange Consortium via the PRIDE [64] partner repository with the dataset identifiers PXD044960 and 10.6019/PXD044960 [65]. Additional file 2 Table S5 contains the numbers of peptides detected by MS for proteins enriched in the gene ontology category of DNA repair.

### Statistical analyses

Statistical analyses were performed using GraphPad Prism v.9.5 or RStudio v.4.0.5. The results were tested for normal distribution (Shapiro test) and homogeneity of variance (Bartlett test). An unpaired *t*-test was used for comparing two groups. For comparison of more than two groups, the ANOVA test was used, with Tukey’s HSD test employed for post hoc analysis. For data not meeting the criteria of parametric tests, the Kruskal–Wallis test and Wilcoxon rank sum test were used. *p* < 0.05 was considered significant. Correlations were assessed using Pearson’s *r* coefficient. The illustrations were created using BioRender or Inkscape software. The statistical details of the experiments can be found in the figure legends. Asterisks indicate the level of statistical significance: *p ≤ 0.05, **p ≤ 0.01, ***p ≤ 0.001.

## Supplementary Information

Additional file 1. Sequences of products resulting from HTT gene editing using HTT_sgRNA1.

Additional file 2: Table S1 Restriction enzymes used in resection analysis. Table S2 Inhibitors and siRNAs used in the study. Table S3 Oligonucleotides used in the study. Table S4 Antibodies used in the study. Table S5 Number of peptides detected by MS for proteins enriched or depleted in gene ontology category of DNA repair. Fig. S1 Separation of PCR products amplifying the CAG repeat sequence in the HTT gene by capillary electrophoresis. Fig. S2 siRNA-mediated knockdown efficiency confirmed by western blotting or RT-qPCR. Fig. S3 EnChIP validation. Fig. S4 Volcano plot and STRING network of all proteins identified in enChIP-MS analysis after introduction of DSBs by using HTT_sgRNA1. Fig. S5 Volcano plot and STRING network of all proteins identified in enChIP-MS analysis after introduction of DSBs by using HTT_sgRNA2. Fig. S6 Presence of DNA:RNA hybrids in the process of DSB repair at the HTT locus.

Additional file 3. Individual data values from the experiments.

Additional file 4. Uncropped images of gels and western blots.

## Data Availability

All data generated or analyzed during this study are included in this published article, its supplementary information files, and publicly available repositories. Individual data values are provided in Additional file 3. Uncropped images are provided in Additional file 4. The targeted sequencing data were deposited in the Sequence Read Archive under the accession number: PRJNA1006315 [61]. The mass spectrometry proteomics data were deposited in the ProteomeXchange Consortium via the PRIDE [64] partner repository with the dataset identifiers PXD044960 and 10.6019/PXD044960 [65].

## Abbreviations

HD: Huntington’s disease
HTT: Huntingtin
DSB: Double-strand break
NHEJ: Nonhomologous end joining
TMEJ: Theta-mediated end joining
MMEJ: Microhomology-mediated end joining
SSA: Single-strand annealing
HDR: Homology-directed repair
BIR: Break-induced replication
HR: Homologous recombination
RNP: Ribonucleoprotein
enChIP: Engineered DNA-binding molecule-mediated chromatin immunoprecipitation
PAM: Protospacer adjacent motif
SpCas9: *Streptococcus pyogenes* Cas9

## References

[1] Biscotti MA, Olmo E, Heslop-Harrison Pat JS. Repetitive DNA in eukaryotic genomes. Chromosome Res. 2015;23(3):415–20. 26514350 10.1007/s10577-015-9499-z

[2] Gemayel R, Vinces MD, Legendre M, Verstrepen KJ. Variable tandem repeats accelerate evolution of coding and regulatory sequences. Annu Rev Genet. 2010;44(1):445–77. 20809801 10.1146/annurev-genet-072610-155046

[3] Khristich AN, Mirkin SM. On the wrong DNA track: molecular mechanisms of repeat-mediated genome instability. J Biol Chem. 2020;295(13):4134–70. 32060097 10.1074/jbc.REV119.007678 PMC7105313

[4] Tabrizi SJ, Schobel S, Gantman EC, Mansbach A, Borowsky B, Konstantinova P, et al. A biological classification of Huntington’s disease: the Integrated Staging System. Lancet Neurol. 2022;21(7):632–44. 35716693 10.1016/S1474-4422(22)00120-X

[5] Richard GF, Dujon B, Haber JE. Double-strand break repair can lead to high frequencies of deletions within short CAG/CTG trinucleotide repeats. Mol Gen Genet. 1999;261(4):871–82. 10394925 10.1007/s004380050031

[6] Cinesi C, Aeschbach L, Yang B, Dion V. Contracting CAG/CTG repeats using the CRISPR-Cas9 nickase. Nat Commun. 2016;9(7):13272. 10.1038/ncomms13272 PMC5105158 27827362

[7] Mosbach V, Poggi L, Viterbo D, Charpentier M, Richard GF. TALEN-induced double-strand break repair of CTG trinucleotide repeats. Cell Rep. 2018;22(8):2146–59. 29466740 10.1016/j.celrep.2018.01.083

[8] Mosbach V, Viterbo D, Descorps-Declère S, Poggi L, Vaysse-Zinkhöfer W, Richard GF. Resection and repair of a Cas9 double-strand break at CTG trinucleotide repeats induces local and extensive chromosomal deletions. PLoS Genet. 2020;16(7): e1008924. 32673314 10.1371/journal.pgen.1008924 PMC7413560

[9] Park CY, Halevy T, Lee DR, Sung JJ, Lee JS, Yanuka O, et al. Reversion of FMR1 methylation and silencing by editing the triplet repeats in fragile X iPSC-derived neurons. Cell Rep. 2015;13(2):234–41. 26440889 10.1016/j.celrep.2015.08.084

[10] Xie N, Gong H, Suhl JA, Chopra P, Wang T, Warren ST. Reactivation of FMR1 by CRISPR/Cas9-mediated deletion of the expanded CGG-repeat of the fragile X chromosome. PLoS ONE. 2016;11(10):e0165499. 27768763 10.1371/journal.pone.0165499 PMC5074572

[11] van Agtmaal EL, André LM, Willemse M, Cumming SA, van Kessel IDG, van den Broek WJAA, et al. CRISPR/Cas9-induced (CTG⋅CAG)n repeat instability in the myotonic dystrophy type 1 locus: implications for therapeutic genome editing. Mol Ther. 2017;25(1):24–43. 28129118 10.1016/j.ymthe.2016.10.014 PMC5363205

[12] Freudenreich CH, Kantrow SM, Zakian VA. Expansion and length-dependent fragility of CTG repeats in yeast. Science. 1998;279(5352):853–6. 9452383 10.1126/science.279.5352.853

[13] Alt FW, Schwer B. DNA double-strand breaks as drivers of neural genomic change, function, and disease. DNA Repair (Amst). 2018;71:158–63. 30195640 10.1016/j.dnarep.2018.08.019 PMC6340756

[14] Wright WD, Shah SS, Heyer WD. Homologous recombination and the repair of DNA double-strand breaks. J Biol Chem. 2018;293(27):10524–35. 29599286 10.1074/jbc.TM118.000372 PMC6036207

[15] Bhargava R, Onyango DO, Stark JM. Regulation of single strand annealing and its role in genome maintenance. Trends Genet. 2016;32(9):566–75. 27450436 10.1016/j.tig.2016.06.007 PMC4992407

[16] Kockler ZW, Osia B, Lee R, Musmaker K, Malkova A. Repair of DNA breaks by break-induced replication. Annu Rev Biochem. 2021;20(90):165–91. 10.1146/annurev-biochem-081420-095551 PMC9629446 33792375

[17] Seol JH, Shim EY, Lee SE. Microhomology-mediated end joining: good, bad and ugly. Mutation Research/Fundamental and Molecular Mechanisms of Mutagenesis. 2018;809:81–7. 28754468 10.1016/j.mrfmmm.2017.07.002 PMC6477918

[18] Scully R, Panday A, Elango R, Willis NA. DNA double-strand break repair-pathway choice in somatic mammalian cells. Nat Rev Mol Cell Biol. 2019;20(11):698–714. 31263220 10.1038/s41580-019-0152-0 PMC7315405

[19] Sharma S, Javadekar SM, Pandey M, Srivastava M, Kumari R, Raghavan SC. Homology and enzymatic requirements of microhomology-dependent alternative end joining. Cell Death Dis. 2015;19(6): e1697. 10.1038/cddis.2015.58 PMC4385936 25789972

[20] Mittelman D, Moye C, Morton J, Sykoudis K, Lin Y, Carroll D, et al. Zinc-finger directed double-strand breaks within CAG repeat tracts promote repeat instability in human cells. PNAS. 2009;106(24):9607–12. 19482946 10.1073/pnas.0902420106 PMC2701052

[21] Bétemps L, Descorps-Declère S, Frenoy O, Poggi L, Mosbach V, Tomé S, et al. TALEN-induced contraction of CTG trinucleotide repeats in myotonic dystrophy type 1 cells. bioRxiv; 2023;2023.10.14.562330. Available from: https://www.biorxiv.org/content/10.1101/2023.10.14.562330v1.

[22] Lo Scrudato M, Poulard K, Sourd C, Tomé S, Klein AF, Corre G, et al. Genome editing of expanded CTG repeats within the human *DMPK* gene reduces nuclear RNA foci in the muscle of DM1 mice. Mol Ther. 2019;27(8):1372–88. 31253581 10.1016/j.ymthe.2019.05.021 PMC6697452

[23] Cardinali B, Provenzano C, Izzo M, Voellenkle C, Battistini J, Strimpakos G, et al. Time-controlled and muscle-specific CRISPR/Cas9-mediated deletion of CTG-repeat expansion in the DMPK gene. Mol Ther Nucleic Acids. 2022;8(27):184–99. 10.1016/j.omtn.2021.11.024 PMC8693309 34976437

[24] Monteys AM, Ebanks SA, Keiser MS, Davidson BL. CRISPR/Cas9 editing of the mutant huntingtin allele in vitro and in vivo. Mol Ther. 2017;25(1):12–23. 28129107 10.1016/j.ymthe.2016.11.010 PMC5363210

[25] Duarte F, Vachey G, Caron NS, Sipion M, Rey M, Perrier AL, et al. Limitations of dual-single guide RNA CRISPR strategies for the treatment of central nervous system genetic disorders. Hum Gene Ther. 2023;34(17–18):958–74. 37658843 10.1089/hum.2023.109

[26] Yang S, Chang R, Yang H, Zhao T, Hong Y, Kong HE, et al. CRISPR/Cas9-mediated gene editing ameliorates neurotoxicity in mouse model of Huntington’s disease. J Clin Invest. 2017;127(7):2719–24. 28628038 10.1172/JCI92087 PMC5490741

[27] Oura S, Noda T, Morimura N, Hitoshi S, Nishimasu H, Nagai Y, et al. Precise CAG repeat contraction in a Huntington’s disease mouse model is enabled by gene editing with SpCas9-NG. Commun Biol. 2021;4(1):1–13. 34163001 10.1038/s42003-021-02304-w PMC8222283

[28] Murillo A, Alpaugh M, Larin M, Randall EL, Heraty L, Durairaj RR, et al. Cas9 nickase-mediated contraction of CAG/CTG repeats at multiple disease loci. bioRxiv; 2024;2024.02.19.580669. Available from: https://www.biorxiv.org/content/10.1101/2024.02.19.580669v1.

[29] Dabrowska M, Ciolak A, Kozlowska E, Fiszer A, Olejniczak M. Generation of new isogenic models of Huntington’s disease using CRISPR-Cas9 technology. Int J Mol Sci. 2020;21(5):1854. 32182692 10.3390/ijms21051854 PMC7084361

[30] Panigrahi GB, Slean MM, Simard JP, Pearson CE. Human mismatch repair protein hMutLα is required to repair short slipped-DNAs of trinucleotide repeats. J Biol Chem. 2012;287(50):41844–50. 23086927 10.1074/jbc.M112.420398 PMC3516732

[31] Dabrowska M, Juzwa W, Krzyzosiak WJ, Olejniczak M. Precise excision of the CAG tract from the huntingtin gene by Cas9 nickases. Front Neurosci. 2018;12:75. Available from: https://www.frontiersin.org/articles/10.3389/fnins.2018.00075/full. 10.3389/fnins.2018.00075 PMC5834764 29535594

[32] Dabrowska M, Czubak K, Juzwa W, Krzyzosiak WJ, Olejniczak M, Kozlowski P. qEva-CRISPR: a method for quantitative evaluation of CRISPR/Cas-mediated genome editing in target and off-target sites. Nucleic Acids Res. 2018;46(17):e101–e101. 29878242 10.1093/nar/gky505 PMC6158505

[33] Olejniczak M, Krzyzosiak WJ. Genotyping of simple sequence repeat factors implicated in shadow band generation revisited. Electrophoresis. 2006;27(19):3724–34. 16960838 10.1002/elps.200600136

[34] Chakrabarti AM, Henser-Brownhill T, Monserrat J, Poetsch AR, Luscombe NM, Scaffidi P. Target-specific precision of CRISPR-mediated genome editing. Mol Cell. 2019;73(4):699–713.e6. 30554945 10.1016/j.molcel.2018.11.031 PMC6395888

[35] Stroik S, Carvajal-Garcia J, Gupta D, Edwards A, Luthman A, Wyatt DW, et al. Stepwise requirements for polymerases δ and θ in theta-mediated end joining. Nature. 2023;15:1–6. 10.1038/s41586-023-06729-7 PMC10959172 37968395

[36] Schimmel J, van Schendel R, den Dunnen JT, Tijsterman M. Templated insertions: a smoking gun for polymerase theta-mediated end joining. Trends Genet. 2019;35(9):632–44. 31296341 10.1016/j.tig.2019.06.001

[37] Zierhut C, Diffley JFX. Break dosage, cell cycle stage and DNA replication influence DNA double strand break response. EMBO J. 2008;27(13):1875–85. 18511906 10.1038/emboj.2008.111 PMC2413190

[38] Singh P, Zheng L, Chavez V, Qiu J, Shen B. Concerted action of exonuclease and Gap-dependent endonuclease activities of FEN-1 contributes to the resolution of triplet repeat sequences (CTG)n- and (GAA)n-derived secondary structures formed during maturation of Okazaki fragments. J Biol Chem. 2007;282(6):3465–77. 17138563 10.1074/jbc.M606582200

[39] Spiro C, Pelletier R, Rolfsmeier ML, Dixon MJ, Lahue RS, Gupta G, et al. Inhibition of FEN-1 processing by DNA secondary structure at trinucleotide repeats. Mol Cell. 1999;4(6):1079–85. 10635332 10.1016/s1097-2765(00)80236-1

[40] Fujita T, Fujii H. Efficient isolation of specific genomic regions and identification of associated proteins by engineered DNA-binding molecule-mediated chromatin immunoprecipitation (enChIP) using CRISPR. Biochem Biophys Res Commun. 2013;439(1):132–6. 23942116 10.1016/j.bbrc.2013.08.013

[41] Szklarczyk D, Gable AL, Lyon D, Junge A, Wyder S, Huerta-Cepas J, et al. STRING v11: protein–protein association networks with increased coverage, supporting functional discovery in genome-wide experimental datasets. Nucleic Acids Res. 2019;47(D1):D607–13. 30476243 10.1093/nar/gky1131 PMC6323986

[42] Santos-Pereira JM, Aguilera A. R loops: new modulators of genome dynamics and function. Nat Rev Genet. 2015;16(10):583–97. 26370899 10.1038/nrg3961

[43] Halász L, Karányi Z, Boros-Oláh B, Kuik-Rózsa T, Sipos É, Nagy É, et al. RNA-DNA hybrid (R-loop) immunoprecipitation mapping: an analytical workflow to evaluate inherent biases. Genome Res. 2017;27(6):1063–73. 28341774 10.1101/gr.219394.116 PMC5453320

[44] Clarke R, Heler R, MacDougall MS, Yeo NC, Chavez A, Regan M, et al. Enhanced bacterial immunity and mammalian genome editing via RNA-polymerase-mediated dislodging of Cas9 from double-strand DNA breaks. Mol Cell. 2018;71(1):42–55.e8. 29979968 10.1016/j.molcel.2018.06.005 PMC6063522

[45] van Overbeek M, Capurso D, Carter MM, Thompson MS, Frias E, Russ C, et al. DNA repair profiling reveals nonrandom outcomes at Cas9-mediated breaks. Mol Cell. 2016;63(4):633–46. 27499295 10.1016/j.molcel.2016.06.037

[46] Guo T, Feng YL, Xiao JJ, Liu Q, Sun XN, Xiang JF, et al. Harnessing accurate non-homologous end joining for efficient precise deletion in CRISPR/Cas9-mediated genome editing. Genome Biol. 2018;19(1):170. 30340517 10.1186/s13059-018-1518-x PMC6195759

[47] Dehé PM, Gaillard PHL. Control of structure-specific endonucleases to maintain genome stability. Nat Rev Mol Cell Biol. 2017;18(5):315–30. 28327556 10.1038/nrm.2016.177

[48] Schrempf A, Slyskova J, Loizou JI. Targeting the DNA repair enzyme polymerase θ in cancer therapy. Trends in Cancer. 2021;7(2):98–111. 33109489 10.1016/j.trecan.2020.09.007

[49] Cerqueira PG, Meyer D, Zhang L, Mallory B, Liu J, Hua Fu BX, et al. Saccharomyces cerevisiae DNA polymerase IV overcomes Rad51 inhibition of DNA polymerase δ in Rad52-mediated direct-repeat recombination. Nucleic Acids Res. 2023;51(11):5547–64. 37070185 10.1093/nar/gkad281 PMC10287921

[50] Liu Y, Prasad R, Beard WA, Hou EW, Horton JK, McMurray CT, et al. Coordination between polymerase β and FEN1 can modulate CAG repeat expansion. J Biol Chem. 2009;284(41):28352–66. 19674974 10.1074/jbc.M109.050286 PMC2788885

[51] Goula AV, Berquist BR, Wilson DM, Wheeler VC, Trottier Y, Merienne K. Stoichiometry of base excision repair proteins correlates with increased somatic CAG instability in striatum over cerebellum in Huntington’s disease transgenic mice. PLoS Genet. 2009;5(12):e1000749. 19997493 10.1371/journal.pgen.1000749 PMC2778875

[52] Goula AV, Pearson CE, Della Maria J, Trottier Y, Tomkinson AE, Wilson DM, et al. The nucleotide sequence, DNA damage location, and protein stoichiometry influence the base excision repair outcome at CAG/CTG repeats. Biochemistry. 2012;51(18):3919–32. 22497302 10.1021/bi300410d PMC3357312

[53] Xu M, Lai Y, Torner J, Zhang Y, Zhang Z, Liu Y. Base excision repair of oxidative DNA damage coupled with removal of a CAG repeat hairpin attenuates trinucleotide repeat expansion. Nucleic Acids Res. 2014;42(6):3675–91. 24423876 10.1093/nar/gkt1372 PMC3973345

[54] Crespan E, Hübscher U, Maga G. Expansion of CAG triplet repeats by human DNA polymerases λ and β in vitro, is regulated by flap endonuclease 1 and DNA ligase 1. DNA Repair (Amst). 2015;29:101–11. 25687118 10.1016/j.dnarep.2015.01.005

[55] Sebesta M, Burkovics P, Juhasz S, Zhang S, Szabo JE, Lee MYWT, et al. Role of PCNA and TLS polymerases in D-loop extension during homologous recombination in humans. DNA Repair (Amst). 2013;12(9):691–8. 23731732 10.1016/j.dnarep.2013.05.001 PMC3744802

[56] Madru C, Henneke G, Raia P, Hugonneau-Beaufet I, Pehau-Arnaudet G, England P, et al. Structural basis for the increased processivity of D-family DNA polymerases in complex with PCNA. Nat Commun. 2020;11(1):1591. 32221299 10.1038/s41467-020-15392-9 PMC7101311

[57] Musio A. The multiple facets of the SMC1A gene. Gene. 2020;15(743): 144612. 10.1016/j.gene.2020.144612 PMC8011328 32222533

[58] Chapados BR, Hosfield DJ, Han S, Qiu J, Yelent B, Shen B, et al. Structural basis for FEN-1 substrate specificity and PCNA-mediated activation in DNA replication and repair. Cell. 2004;116(1):39–50. 14718165 10.1016/s0092-8674(03)01036-5

[59] Johnson M, Whalen J, Freudenreich C. The role of the DNA damage checkpoint in the relocalization of CAG trinucleotide repeats to the nuclear pore complex during S-phase. FASEB J. 2020;34(S1):1–1.

[60] Wang AS, Chen LC, Wu RA, Hao Y, McSwiggen DT, Heckert AB, et al. The histone chaperone FACT induces Cas9 multi-turnover behavior and modifies genome manipulation in human cells. Mol Cell. 2020;79(2):221–233.e5. 32603710 10.1016/j.molcel.2020.06.014 PMC7398558

[61] Investigation of DNA double-strand break repair mechanisms in microsatellite regions using the CRISPR/Cas9 system. Sequence Read Archive (SRA) https://www.ncbi.nlm.nih.gov/bioproject/?term=PRJNA1006315 (2023).

[62] Li W, Godzik A. Cd-hit: a fast program for clustering and comparing large sets of protein or nucleotide sequences. Bioinformatics. 2006;22(13):1658–9. 16731699 10.1093/bioinformatics/btl158

[63] Jancewicz I, Szarkowska J, Konopinski R, Stachowiak M, Swiatek M, Blachnio K, et al. PD-L1 overexpression, SWI/SNF complex deregulation, and profound transcriptomic changes characterize cancer-dependent exhaustion of persistently activated CD4+ T cells. Cancers. 2021;13(16):4148. 34439305 10.3390/cancers13164148 PMC8391521

[64] Perez-Riverol Y, Bai J, Bandla C, García-Seisdedos D, Hewapathirana S, Kamatchinathan S, et al. The PRIDE database resources in 2022: a hub for mass spectrometry-based proteomics evidences. Nucleic Acids Res. 2022;50(D1):D543–52. 34723319 10.1093/nar/gkab1038 PMC8728295

[65] Investigation of DNA double-strand break repair mechanisms in microsatellite regions using the CRISPR/Cas9 system. 2023. ProteomeXchange Consortium. 10.6019/PXD044960.

