# Supplementary material for: CRISPR/Cas9-induced double-strand breaks in the huntingtin locus lead to CAG repeat contraction through DNA end resection and homology-mediated repair
Source: BMC Biol. 2024 Dec 3;22:282. doi: 10.1186/s12915-024-02079-6 (PMC11616332; doi:10.1186/s12915-024-02079-6)
Supplement: Supplementary file 1 — Additional file 1. Sequences of products resulting from HTT gene editing using HTT_sgRNA1. [file 12915_2024_2079_MOESM1_ESM.pdf]

Legend:
Microhomologous regions in red
Templated insertions pattern characteristic for TMEJ in blue

T#ID	CAG	WT Sequence	RGEN Treated Sequence	Length	Count	Type
109	40.0	GCTGATGAAGGCCTTCGAGTCCCT**CAAGTCCTTCC**-AGCAGCAGCAGCAGCAGCAGCAGCAGCAGCAGCAGCAGCAGCAGCAGCAGCAGCAGCAGCAGCAGCAGCAGCAGCAGCAGCAGCAGCAGCAGCAGCAGCAGCAGCAGCAGCAGCAGCAGCAGCAGCAG**CAACAGCCG**CCACCGCCGCCGCCGCCGCCGCCGCCTCCTCAGCTTCCTCAGCCGCCGCCGCAGGCACAGCCGCTGCTGCCTCAGC	GCTGATGAAGGCCTTCGAGTCCCT**CAAGTCCTTCC**AAGCAGCAGCAGCAGCAGCAGCAGCAGCAGCAGCAGCAGCAGCAGCAGCAGCAGCAGCAGCAGCAGCAGCAGCAGCAGCAGCAGCAGCAGCAGCAGCAGCAGCAGCAGCAGCAGCAGCAGCAGCAGCAG**CAACAGCCG**CCACCGCCGCCGCCGCCGCCGCCGCCTCCTCAGCTTCCTCAGCCGCCGCCGCAGGCACAGCCGCTGCTGCCTCAGC	242	23396	INS
125	39.0	GCTGATGAAGGCCTTCGAGTCCCT**CAAGTCCTTCC**AGCAGCAGCAGCAGCAGCAGCAGCAGCAGCAGCAGCAGCAGCAGCAGCAGCAGCAGCAGCAGCAGCAGCAGCAGCAGCAGCAGCAGCAGCAGCAGCAGCAGCAGCAGCAGCAGCAGCAGCAGCAGCAG**CAACAGCCG**CCACCGCCGCCGCCGCCGCCGCCGCCTCCTCAGCTTCCTCAGCCGCCGCCGCAGGCACAGCCGCTGCTGCCTCAGC	GCTGATGAAGGCCTTCGAGTCCCT**CAAGTCCTTCC**A--AGCAGCAGCAGCAGCAGCAGCAGCAGCAGCAGCAGCAGCAGCAGCAGCAGCAGCAGCAGCAGCAGCAGCAGCAGCAGCAGCAGCAGCAGCAGCAGCAGCAGCAGCAGCAGCAGCAGCAGCAGCAG**CAACAGCCG**CCACCGCCGCCGCCGCCGCCGCCGCCTCCTCAGCTTCCTCAGCCGCCGCCGCAGGCACAGCCGCTGCTGCCTCAGC	242	15100	DEL
1300	0.0	GCTGATGAAGGCCTTCGAGTCCCT**CAAGTCCTTCC**AGCAGCAGCAGCAGCAGCAGCAGCAGCAGCAGCAGCAGCAGCAGCAGCAGCAGCAGCAGCAGCAGCAGCAGCAGCAGCAGCAGCAGCAGCAGCAGCAGCAGCAGCAGCAGCAGCAGCAGCAGCAGCAG**CAACAGCCG**CCACCGCCGCCGCCGCCGCCGCCGCCTCCTCAGCTTCCTCAGCCGCCGCCGCAGGCACAGCCGCTGCTGCCTCAGC	GCTGATGAAGGCCTTCGAGTCCCTCAAGTCCTT--CCGCCGCCGCCGCCGCCTCCTCAGCTTCCTCAGCCGCCGCCGCAGGCACAGCCGCTGCTGCCTCAGC	242	8849	DEL
1335	0.0	GCTGATGAAGGCCTTCGAGTCCCT**CAAGTCCTTCC**AGCAGCAGCAGCAGCAGCAGCAGCAGCAGCAGCAGCAGCAGCAGCAGCAGCAGCAGCAGCAGCAGCAGCAGCAGCAGCAGCAGCAGCAGCAGCAGCAGCAGCAGCAGCAGCAGCAGCAGCAGCAGCAG**CAACAGCCG**CCACCGCCGCCGCCGCCGCCGCCGCCTCCTCAGCTTCCTCAGCCGCCGCCGCAGGCACAGCCGCTGCTGCCTCAGC	GCTGATGAAGGCCTTCGAGTCCCTCAAGT--CCGCCGCCGCCGCCGCCTCCTCAGCTTCCTCAGCCGCCGCCGCAGGCACAGCCGCTGCTGCCTCAGC	242	7905	DEL
1351	0.0	GCTGATGAAGGCCTTCGAGTCCCT**CAAGTCCTTCC**AGCAGCAGCAGCAGCAGCAGCAGCAGCAGCAGCAGCAGCAGCAGCAGCAGCAGCAGCAGCAGCAGCAGCAGCAGCAGCAGCAGCAGCAGCAGCAGCAGCAGCAGCAGCAGCAGCAGCAGCAGCAGCAG**CAACAGCCG**CCACCGCCGCCGCCGCCGCCGCCGCCTCCTCAGCTTCCTCAGCCGCCGCCGCAGGCACAGCCGCTGCTGCCTCAGC	GCTGATGAAGGCCTTCGAGTCCCTCAAGTCCTT--CCGGCGCCGCCTCCTCAGCTTCCTCAGCCGCCGCCGCAGGCACAGCCGCTGCTGCCTCAGC	242	7867	DEL
1492	0.0	GCTGATGAAGGCCTTCGAGTCCCT**CAAGTCCTTCC**AGCAGCAGCAGCAGCAGCAGCAGCAGCAGCAGCAGCAGCAGCAGCAGCAGCAGCAGCAGCAGCAGCAGCAGCAGCAGCAGCAGCAGCAGCAGCAGCAGCAGCAGCAGCAGCAGCAGCAGCAGCAGCAG**CAACAGCCG**CCACCGCCGCCGCCGCCGCCGCCGCCTCCTCAGCTTCCTCAGCCGCCGCCGCAGGCACAGCCGCTGCTGCCTCAGC	GCTGATGAAGGCCTTCGAGTC--CCTCAGCTTCCTCAGCCGCCGCCGCAGGCACAGCCGCTGCTGCCTCAGC	242	7625	DEL
1243	0.0	GCTGATGAAGGCCTTCGAGTCCCT**CAAGTCCTTCC**AGCAGCAGCAGCAGCAGCAGCAGCAGCAGCAGCAGCAGCAGCAGCAGCAGCAGCAGCAGCAGCAGCAGCAGCAGCAGCAGCAGCAGCAGCAGCAGCAGCAGCAGCAGCAGCAGCAGCAGCAGCAGCAG**CAACAGCCG**CCACCGCCGCCGCCGCCGCCGCCGCCTCCTCAGCTTCCTCAGCCGCCGCCGCAGGCACAGCCGCTGCTGCCTCAGC	GCTGATGAAGGCCTTCGAGTCCCTCAAGTCCTT--CCGCCGCCGCCGCCGCCGCCGCCTCCTCAGCTTCCTCAGCCGCCGCCGCAGGCACAGCCGCTGCTGCCTCAGC	242	6340	DEL
1214	0.0	GCTGATGAAGGCCTTCGAGTCCCT**CAAGTCCTTCC**AGCAGCAGCAGCAGCAGCAGCAGCAGCAGCAGCAGCAGCAGCAGCAGCAGCAGCAGCAGCAGCAGCAGCAGCAGCAGCAGCAGCAGCAGCAGCAGCAGCAGCAGCAGCAGCAGCAGCAGCAGCAGCAG**CAACAGCCG**CCACCGCCGCCGCCGCCGCCGCCGCCTCCTCAGCTTCCTCAGCCGCCGCCGCAGGCACAGCCGCTGCTGCCTCAGC	GCTGATGAAGGCCTTCGAGTCCCTCAAGTCCTT---CCACCGCCGCCGCCGCCGCCGCCGCCTCCTCAGCTTCCTCAGCCGCCGCCGCAGGCACAGCCGCTGCTGCCTCAGC	242	6280	DEL
1256	0.0	GCTGATGAAGGCCTTCGAGTCCCT**CAAGTCCTTCC**AGCAGCAGCAGCAGCAGCAGCAGCAGCAGCAGCAGCAGCAGCAGCAGCAGCAGCAGCAGCAGCAGCAGCAGCAGCAGCAGCAGCAGCAGCAGCAGCAGCAGCAGCAGCAGCAGCAGCAGCAGCAGCAG**CAACAGCCG**CCACCGCCGCCGCCGCCGCCGCCGCCTCCTCAGCTTCCTCAGCCGCCGCCGCAGGCACAGCCGCTGCTGCCTCAGC	GCTGATGAAGGCCTTCGAGTCCCTCAAGT---CCACCGCCGCCGCCGCCGCCGCCGCCTCCTCAGCTTCCTCAGCCGCCGCCGCAGGCACAGCCGCTGCTGCCTCAGC	242	6097	DEL
168	38.0	GCTGATGAAGGCCTTCGAGTCCCT**CAAGTCCTTCC**AGCAGCAGCAGCAGCAGCAGCAGCAGCAGCAGCAGCAGCAGCAGCAGCAGCAGCAGCAGCAGCAGCAGCAGCAGCAGCAGCAGCAGCAGCAGCAGCAGCAGCAGCAGCAGCAGCAGCAGCAGCAGCAG**CAACAGCCG**CCACCGCCGCCGCCGCCGCCGCCGCCTCCTCAGCTTCCTCAGCCGCCGCCGCAGGCACAGCCGCTGCTGCCTCAGC	GCTGATGAAGGCCTTCGAGTCCCT**CAAGTCCTTCC**A-----AGCAGCAGCAGCAGCAGCAGCAGCAGCAGCAGCAGCAGCAGCAGCAGCAGCAGCAGCAGCAGCAGCAGCAGCAGCAGCAGCAGCAGCAGCAGCAGCAGCAGCAGCAGCAGCAGCAGCAG**CAACAGCCG**CCACCGCCGCCGCCGCCGCCGCCGCCTCCTCAGCTTCCTCAGCCGCCGCCGCAGGCACAGCCGCTGCTGCCTCAGC	242	5817	DEL
116	41.0	GCTGATGAAGGCCTTCGAGTCCCT**CAAGTCCTTCC**AGCAGCAGCAGCAGCAGCAGCAGCAGCAGCAGCAGCAGCAGCAGCAGCAGCAGCAGCAGCAGCAGCAGCAGCAGCAGCAGCAGCAGCAGCAGCAGCAGCAGCAGCAGCAGCAGCAGCAGCAGCAGCAG**CAACAGCCG**CCACCGCCGCCGCCGCCGCCGCCGCCTCCTCAGCTTCCTCAGCCGCCGCCGCAGGCACAGCCGCTGCTGCCTCAGC	GCTGATGAAGGCCTTCGAGTCCCT**CAAGTCCTTCC**AGCAGCAGCAGCAGCAGCAGCAGCAGCAGCAGCAGCAGCAGCAGCAGCAGCAGCAGCAGCAGCAGCAGCAGCAGCAGCAGCAGCAGCAGCAGCAGCAGCAGCAGCAGCAGCAGCAGCAGCAGCAGCAG**CAACAGCCG**CCACCGCCGCCGCCGCCGCCGCCGCCTCCTCAGCTTCCTCAGCCGCCGCCGCAGGCACAGCCGCTGCTGCCTCAGC	242	5776	WT/SUB
1276	0.0	GCTGATGAAGGCCTTCGAGTCCCT**CAAGTCCTTCC**AGCAGCAGCAGCAGCAGCAGCAGCAGCAGCAGCAGCAGCAGCAGCAGCAGCAGCAGCAGCAGCAGCAGCAGCAGCAGCAGCAGCAGCAGCAGCAGCAGCAGCAGCAGCAGCAGCAGCAGCAGCAGCAG**CAACAGCCG**CCACCGCCGCCGCCGCCGCCGCCGCCTCCTCAGCTTCCTCAGCCGCCGCCGCAGGCACAGCCGCTGCTGCCTCAGC	GCTGATGAAGGCCTTCGAGTCCCTCAAGTCCTT---CCGCCGCCGCCGCCGCCGCCTCCTCAGCTTCCTCAGCCGCCGCCGCAGGCACAGCCGCTGCTGCCTCAGC	242	5326	DEL
1325	0.0	GCTGATGAAGGCCTTCGAGTCCCT**CAAGTCCTTCC**AGCAGCAGCAGCAGCAGCAGCAGCAGCAGCAGCAGCAGCAGCAGCAGCAGCAGCAGCAGCAGCAGCAGCAGCAGCAGCAGCAGCAGCAGCAGCAGCAGCAGCAGCAGCAGCAGCAGCAGCAGCAGCAG**CAACAGCCG**CCACCGCCGCCGCCGCCGCCGCCGCCTCCTCAGCTTCCTCAGCCGCCGCCGCAGGCACAGCCGCTGCTGCCTCAGC	GCTGATGAAGGCCTTCGAGTCCCTCAAGTCCTT---CCGCCGCCGCCGCCTCCTCAGCTTCCTCAGCCGCCGCCGCAGGCACAGCCGCTGCTGCCTCAGC	242	5079	DEL
1307	0.0	GCTGATGAAGGCCTTCGAGTCCCT**CAAGTCCTTCC**AGCAGCAGCAGCAGCAGCAGCAGCAGCAGCAGCAGCAGCAGCAGCAGCAGCAGCAGCAGCAGCAGCAGCAGCAGCAGCAGCAGCAGCAGCAGCAGCAGCAGCAGCAGCAGCAGCAGCAGCAGCAGCAG**CAACAGCCG**CCACCGCCGCCGCCGCCGCCGCCGCCTCCTCAGCTTCCTCAGCCGCCGCCGCAGGCACAGCCGCTGCTGCCTCAGC	GCTGATGAAGGCCTTCGAGTCCCTCAAGTC---TCAAGCCGCCGCCGCCGCCTCCTCAGCTTCCTCAGCCGCCGCCGCAGGCACAGCCGCTGCTGCCTCAGC	242	4892	DEL
1455	0.0	GCTGATGAAGGCCTTCGAGTCCCT**CAAGTCCTTCC**AGCAGCAGCAGCAGCAGCAGCAGCAGCAGCAGCAGCAGCAGCAGCAGCAGCAGCAGCAGCAGCAGCAGCAGCAGCAGCAGCAGCAGCAGCAGCAGCAGCAGCAGCAGCAGCAGCAGCAGCAGCAGCAG**CAACAGCCG**CCACCGCCGCCGCCGCCGCCGCCGCCTCCTCAGCTTCCTCAGCCGCCGCCGCAGGCACAGCCGCTGCTGCCTCAGC	GCTGATGAAGGCCTTCGAGTCCCTCAAG--TCCTCAGCTTCCTCAGCCGCCGCCGCAGGCACAGCCGCTGCTGCCTCAGC	242	4424	DEL
1285	0.0	GCTGATGAAGGCCTTCGAGTCCCT**CAAGTCCTTCC**AGCAGCAGCAGCAGCAGCAGCAGCAGCAGCAGCAGCAGCAGCAGCAGCAGCAGCAGCAGCAGCAGCAGCAGCAGCAGCAGCAGCAGCAGCAGCAGCAGCAGCAGCAGCAGCAGCAGCAGCAGCAGCAG**CAACAGCCG**CCACCGCCGCCGCCGCCGCCGCCGCCTCCTCAGCTTCCTCAGCCGCCGCCGCAGGCACAGCCGCTGCTGCCTCAGC	GCTGATGAAGGCCTTCGAGTCCCTCAAGT--CCGCCGCCGCCGCCGCCGCCGCCTCCTCAGCTTCCTCAGCCGCCGCCGCAGGCACAGCCGCTGCTGCCTCAGC	242	4176	DEL
1501	0.0	GCTGATGAAGGCCTTCGAGTCCCT**CAAGTCCTTCC**AGCAGCAGCAGCAGCAGCAGCAGCAGCAGCAGCAGCAGCAGCAGCAGCAGCAGCAGCAGCAGCAGCAGCAGCAGCAGCAGCAGCAGCAGCAGCAGCAGCAGCAGCAGCAGCAGCAGCAGCAGCAGCAG**CAACAGCCG**CCACCGCCGCCGCCGCCGCCGCCGCCTCCTCAGCTTCCTCAGCCGCCGCCGCAGGCACAGCCGCTGCTGCCTCAGC	GCTGATGAAGGCCTTCGAGTCCCTCAAG---TCCTCAGCCGCCGCCGCAGGCACAGCCGCTGCTGCCTCAGC	242	3928	DEL
1220	0.0	GCTGATGAAGGCCTTCGAGTCCCT**CAAGTCCTTCC**AGCAGCAGCAGCAGCAGCAGCAGCAGCAGCAGCAGCAGCAGCAGCAGCAGCAGCAGCAGCAGCAGCAGCAGCAGCAGCAGCAGCAGCAGCAGCAGCAGCAGCAGCAGCAGCAGCAGCAGCAGCAGCAG**CAACAGCCG**CCACCGCCGCCGCCGCCGCCGCCGCCTCCTCAGCTTCCTCAGCCGCCGCCGCAGGCACAGCCGCTGCTGCCTCAGC	GCTGATGAAGGCCTTCGAGTCCCTCAAGT--CCGCCACCGCCGCCGCCGCCGCCGCCGCCTCCTCAGCTTCCTCAGCCGCCGCCGCAGGCACAGCCGCTGCTGCCTCAGC	242	3466	DEL
134	40.0	GCTGATGAAGGCCTTCGAGTCCCT**CAAGTCCTTCC**AGCAGCAGCAGCAGCAGCAGCAGCAGCAGCAGCAGCAGCAGCAGCAGCAGCAGCAGCAGCAGCAGCAGCAGCAGCAGCAGCAGCAGCAGCAGCAGCAGCAGCAGCAGCAGCAGCAGCAGCAGCAGCAG**CAACAGCCG**CCACCGCCGCCGCCGCCGCCGCCGCCTCCTCAGCTTCCTCAGCCGCCGCCGCAGGCACAGCCGCTGCTGCCTCAGC	GCTGATGAAGGCCTTCGAGTCCCTCAAGTCCTTC---CAGCAGCAGCAGCAGCAGCAGCAGCAGCAGCAGCAGCAGCAGCAGCAGCAGCAGCAGCAGCAGCAGCAGCAGCAGCAGCAGCAGCAGCAGCAGCAGCAGCAGCAGCAGCAGCAGCAGCAGCAGCAG**CAACAGCCG**CCACCGCCGCCGCCGCCGCCGCCGCCTCCTCAGCTTCCTCAGCCGCCGCCGCAGGCACAGCCGCTGCTGCCTCAGC	242	3211	DEL
92	41.0	GCTGATGAAGGCCTTCGAGTCCCTCAAGTCCTTC----CAGCAGCAGCAGCAGCAGCAGCAGCAGCAGCAGCAGCAGCAGCAGCAGCAGCAGCAGCAGCAGCAGCAGCAGCAGCAGCAGCAGCAGCAGCAGCAGCAGCAGCAGCAGCAGCAGCAGCAGCAGCAGCAGCAG**CAACAGCCG**CCACCGCCGCCGCCGCCGCCGCCGCCTCCTCAGCTTCCTCAGCCGCCGCCGCAGGCACAGCCGCTGCTGCCTCAGC	GCTGATGAAGGCCTTCGAGTCCCT**CAAGTCCTTCC**AAGCAGCAGCAGCAGCAGCAGCAGCAGCAGCAGCAGCAGCAGCAGCAGCAGCAGCAGCAGCAGCAGCAGCAGCAGCAGCAGCAGCAGCAGCAGCAGCAGCAGCAGCAGCAGCAGCAGCAGCAGCAGCAGCAG**CAACAGCCG**CCACCGCCGCCGCCGCCGCCGCCGCCTCCTCAGCTTCCTCAGCCGCCGCCGCAGGCACAGCCGCTGCTGCCTCAGC	242	3076	INS
1359	0.0	GCTGATGAAGGCCTTCGAGTCCCT**CAAGTCCTTCC**AGCAGCAGCAGCAGCAGCAGCAGCAGCAGCAGCAGCAGCAGCAGCAGCAGCAGCAGCAGCAGCAGCAGCAGCAGCAGCAGCAGCAGCAGCAGCAGCAGCAGCAGCAGCAGCAGCAGCAGCAGCAGCAG**CAACAGCCG**CCACCGCCGCCGCCGCCGCCGCCGCCTCCTCAGCTTCCTCAGCCGCCGCCGCAGGCACAGCCGCTGCTGCCTCAGC	GCTGATGAAGGCCTTCGAGTCCCTCAAGT---CCGCCGCCGCCGCCTCCTCAGCTTCCTCAGCCGCCGCCGCAGGCACAGCCGCTGCTGCCTCAGC	242	3068	DEL
1233	0.0	GCTGATGAAGGCCTTCGAGTCCCT**CAAGTCCTTCC**AGCAGCAGCAGCAGCAGCAGCAGCAGCAGCAGCAGCAGCAGCAGCAGCAGCAGCAGCAGCAGCAGCAGCAGCAGCAGCAGCAGCAGCAGCAGCAGCAGCAGCAGCAGCAGCAGCAGCAGCAGCAGCAG**CAACAGCCG**CCACCGCCGCCGCCGCCGCCGCCGCCTCCTCAGCTTCCTCAGCCGCCGCCGCAGGCACAGCCGCTGCTGCCTCAGC	GCTGATGAAGGCCTTCGAGTCCCTCA---AGCCGCCACCGCCGCCGCCGCCGCCGCCGCCTCCTCAGCTTCCTCAGCCGCCGCCGCAGGCACAGCCGCTGCTGCCTCAGC	242	3048	DEL
1452	0.0	GCTGATGAAGGCCTTCGAGTCCCT**CAAGTCCTTCC**AGCAGCAGCAGCAGCAGCAGCAGCAGCAGCAGCAGCAGCAGCAGCAGCAGCAGCAGCAGCAGCAGCAGCAGCAGCAGCAGCAGCAGCAGCAGCAGCAGCAGCAGCAGCAGCAGCAGCAGCAGCAGCAG**CAACAGCCG**CCACCGCCGCCGCCGCCGCCGCCGCCTCCTCAGCTTCCTCAGCCGCCGCCGCAGGCACAGCCGCTGCTGCCTCAGC	GCTGATGAAGGCCTTCGAGTCCCTCAAGTC---CCTCAGCTTCCTCAGCCGCCGCCGCAGGCACAGCCGCTGCTGCCTCAGC	242	2953	DEL
1366	0.0	GCTGATGAAGGCCTTCGAGTCCCT**CAAGTCCTTCC**AGCAGCAGCAGCAGCAGCAGCAGCAGCAGCAGCAGCAGCAGCAGCAGCAGCAGCAGCAGCAGCAGCAGCAGCAGCAGCAGCAGCAGCAGCAGCAGCAGCAGCAGCAGCAGCAGCAGCAGCAGCAGCAG**CAACAGCCG**CCACCGCCGCCGCCGCCGCCGCCGCCTCCTCAGCTTCCTCAGCCGCCGCCGCAGGCACAGCCGCTGCTGCCTCAGC	GCTGATGAAGGCCTTCGAGTCCCTCAAGT--CGCCGCCGCCGCCTCCTCAGCTTCCTCAGCCGCCGCCGCAGGCACAGCCGCTGCTGCCTCAGC	242	2950	DEL
1443	0.0	GCTGATGAAGGCCTTCGAGTCCCT**CAAGTCCTTCC**AGCAGCAGCAGCAGCAGCAGCAGCAGCAGCAGCAGCAGCAGCAGCAGCAGCAGCAGCAGCAGCAGCAGCAGCAGCAGCAGCAGCAGCAGCAGCAGCAGCAGCAGCAGCAGCAGCAGCAGCAGCAGCAG**CAACAGCCG**CCACCGCCGCCGCCGCCGCCGCCGCCTCCTCAGCTTCCTCAGCCGCCGCCGCAGGCACAGCCGCTGCTGCCTCAGC	GCTGATGAAGGCCTTCGAGTCCCT**CAAGTCCTTCC**---CTGCTTCCTCAGCCGCCGCCGCAGGCACAGCCGCTGCTGCCTCAGC	242	2674	DEL
231	37.0	GCTGATGAAGGCCTTCGAGTCCCT**CAAGTCCTTCC**AGCAGCAGCAGCAGCAGCAGCAGCAGCAGCAGCAGCAGCAGCAGCAGCAGCAGCAGCAGCAGCAGCAGCAGCAGCAGCAGCAGCAGCAGCAGCAGCAGCAGCAGCAGCAGCAGCAGCAGCAGCAGCAG**CAACAGCCG**CCACCGCCGCCGCCGCCGCCGCCGCCTCCTCAGCTTCCTCAGCCGCCGCCGCAGGCACAGCCGCTGCTGCCTCAGC	GCTGATGAAGGCCTTCGAGTCCCT**CAAGTCCTTCC**A--------AGCAGCAGCAGCAGCAGCAGCAGCAGCAGCAGCAGCAGCAGCAGCAGCAGCAGCAGCAGCAGCAGCAGCAGCAGCAGCAGCAGCAGCAGCAGCAGCAGCAGCAGCAGCAGCAG**CAACAGCCG**CCACCGCCGCCGCCGCCGCCGCCGCCTCCTCAGCTTCCTCAGCCGCCGCCGCAGGCACAGCCGCTGCTGCCTCAGC	242	2324	DEL
1266	0.0	GCTGATGAAGGCCTTCGAGTCCCT**CAAGTCCTTCC**AGCAGCAGCAGCAGCAGCAGCAGCAGCAGCAGCAGCAGCAGCAGCAGCAGCAGCAGCAGCAGCAGCAGCAGCAGCAGCAGCAGCAGCAGCAGCAGCAGCAGCAGCAGCAGCAGCAGCAGCAGCAGCAG**CAACAGCCG**CCACCGCCGCCGCCGCCGCCGCCGCCTCCTCAGCTTCCTCAGCCGCCGCCGCAGGCACAGCCGCTGCTGCCTCAGC	GCTGATGAAGGCCTTCGAGTCCCT**CAAGTCCTTCC**--GACTCGCGGCGCCGCCGCCTCCTCAGCTTCCTCAGCCGCCGCCGCAGGCACAGCCGCTGCTGCCTCAGC	242	2254	DEL
1387	0.0	GCTGATGAAGGCCTTCGAGTCCCT**CAAGTCCTTCC**AGCAGCAGCAGCAGCAGCAGCAGCAGCAGCAGCAGCAGCAGCAGCAGCAGCAGCAGCAGCAGCAGCAGCAGCAGCAGCAGCAGCAGCAGCAGCAGCAGCAGCAGCAGCAGCAGCAGCAGCAGCAGCAG**CAACAGCCG**CCACCGCCGCCGCCGCCGCCGCCGCCTCCTCAGCTTCCTCAGCCGCCGCCGCAGGCACAGCCGCTGCTGCCTCAGC	GCTGATGAAGGCCTTCGAGTCCCTCAAGT---CGCCGCCGCCTCCTCAGCTTCCTCAGCCGCCGCCGCAGGCACAGCCGCTGCTGCCTCAGC	242	2134	DEL
1108	0.0	GCTGATGAAGGCCTTCGAGTCCCT**CAAGTCCTTCC**AGCAGCAGCAGCAGCAGCAGCAGCAGCAGCAGCAGCAGCAGCAGCAGCAGCAGCAGCAGCAGCAGCAGCAGCAGCAGCAGCAGCAGCAGCAGCAGCAGCAGCAGCAGCAGCAGCAGCAGCAGCAGCAG**CAACAGCCG**CCACCGCCGCCGCCGCCGCCGCCGCCTCCTCAGCTTCCTCAGCCGCCGCCGCAGGCACAGCCGCTGCTGCCTCAGC	GCTGATGAAGGCCTTCGAGTCCCTCAAGTCCT---CAAG**CAACAGCCG**CCACCGCCGCCGCCGCCGCCGCCGCCTCCTCAGCTTCCTCAGCCGCCGCCGCAGGCACAGCCGCTGCTGCCTCAGC	242	2003	DEL
1409	0.0	GCTGATGAAGGCCTTCGAGTCCCT**CAAGTCCTTCC**AGCAGCAGCAGCAGCAGCAGCAGCAGCAGCAGCAGCAGCAGCAGCAGCAGCAGCAGCAGCAGCAGCAGCAGCAGCAGCAGCAGCAGCAGCAGCAGCAGCAGCAGCAGCAGCAGCAGCAGCAGCAGCAG**CAACAGCCG**CCACCGCCGCCGCCGCCGCCGCCGCCTCCTCAGCTTCCTCAGCCGCCGCCGCAGGCACAGCCGCTGCTGCCTCAGC	GCTGATGAAGGCCTTCGAGTCCCTCAA--GCCGCCGCCTCCTCAGCTTCCTCAGCCGCCGCCGCAGGCACAGCCGCTGCTGCCTCAGC	242	1917	DEL
1317	0.0	GCTGATGAAGGCCTTCGAGTCCCT**CAAGTCCTTCC**AGCAGCAGCAGCAGCAGCAGCAGCAGCAGCAGCAGCAGCAGCAGCAGCAGCAGCAGCAGCAGCAGCAGCAGCAGCAGCAGCAGCAGCAGCAGCAGCAGCAGCAGCAGCAGCAGCAGCAGCAGCAGCAG**CAACAGCCG**CCACCGCCGCCGCCGCCGCCGCCGCCTCCTCAGCTTCCTCAGCCGCCGCCGCAGGCACAGCCGCTGCTGCCTCAGC	GCTGATGAAGGCCTTCGAGTCCCTCAACT--CGAGGCCGCCGCCGCCGCCTCCTCAGCTTCCTCAGCCGCCGCCGCAGGCACAGCCGCTGCTGCCTCAGC	242	1912	DEL
1182	0.0	GCTGATGAAGGCCTTCGAGTCCCT**CAAGTCCTTCC**AGCAGCAGCAGCAGCAGCAGCAGCAGCAGCAGCAGCAGCAGCAGCAGCAGCAGCAGCAGCAGCAGCAGCAGCAGCAGCAGCAGCAGCAGCAGCAGCAGCAGCAGCAGCAGCAGCAGCAGCAGCAGCAG**CAACAGCCG**CCACCGCCGCCGCCGCCGCCGCCGCCTCCTCAGCTTCCTCAGCCGCCGCCGCAGGCACAGCCGCTGCTGCCTCAGC	GCTGATGAAGGCCTTCGAGTCCCTCA---AG**CAACAGCCG**CCACCGCCGCCGCCGCCGCCGCCGCCTCCTCAGCTTCCTCAGCCGCCGCCGCAGGCACAGCCGCTGCTGCCTCAGC	242	1837	DEL
107	41.0	GCTGATGAAGGCCTTCGAGTCCCTCAAGTCCTTC--CAGCAGCAGCAGCAGCAGCAGCAGCAGCAGCAGCAGCAGCAGCAGCAGCAGCAGCAGCAGCAGCAGCAGCAGCAGCAGCAGCAGCAGCAGCAGCAGCAGCAGCAGCAGCAGCAGCAGCAGCAGCAGCAGCAG**CAACAGCCG**CCACCGCCGCCGCCGCCGCCGCCGCCTCCTCAGCTTCCTCAGCCGCCGCCGCAGGCACAGCCGCTGCTGCCTCAGC	GCTGATGAAGGCCTTCGAGTCCCT**CAAGTCCTTCC**ACAGCAGCAGCAGCAGCAGCAGCAGCAGCAGCAGCAGCAGCAGCAGCAGCAGCAGCAGCAGCAGCAGCAGCAGCAGCAGCAGCAGCAGCAGCAGCAGCAGCAGCAGCAGCAGCAGCAGCAGCAGCAGCAG**CAACAGCCG**CCACCGCCGCCGCCGCCGCCGCCGCCTCCTCAGCTTCCTCAGCCGCCGCCGCAGGCACAGCCGCTGCTGCCTCAGC	242	1756	INS
122	40.0					

GCTGATGAAGGCCTTCGAGTCCCT**CAAGTCCTTCC**AGCAGCAGCAGCAGCAGCAGCAGCAGCAGCAGCAGCAGCAGCAGCAGCAGCAGCAGCAGCAGCAGCAGCAGCAGCAGCAGCAGCAGCAGCAGCAGCAGCAGCAGCAGCAGCAGCAGCAGCAGCAGCAG**CAACAGCCG**CCACCGCCGCCGCCGCCGCCGCCGCCTCCTCAGCTTCCTCAGCCGCCGCCGCAGGCACAGCCGCTGCTGCCTCAGC

GCTGATGAAGGCCTTCGAGTCCCT**CAAGTCCTTCC**A-CAGCAGCAGCAGCAGCAGCAGCAGCAGCAGCAGCAGCAGCAGCAGCAGCAGCAGCAGCAGCAGCAGCAGCAGCAGCAGCAGCAGCAGCAGCAGCAGCAGCAGCAGCAGCAGCAGCAGCAGCAG**CAACAGCCG**CCACCGCCGCCGCCGCCGCCGCCGCCTCCTCAGCTTCCTCAGCCGCCGCCGCAGGCACAGCCGCTGCTGCCTCAGC 242 1555 DEL

207 39.0

GCTGATGAAGGCCTTCGAGTCCCT**CAAGTCCTTCC**AGCAGCAGCAGCAGCAGCAGCAGCAGCAGCAGCAGCAGCAGCAGCAGCAGCAGCAGCAGCAGCAGCAGCAGCAGCAGCAGCAGCAGCAGCAGCAGCAGCAGCAGCAGCAGCAGCAGCAGCAGCAG**CAACAGCCG**CCACCGCCGCCGCCGCCGCCGCCGCCTCCTCAGCTTCCTCAGCCGCCGCCGCAGGCACAGCCGCTGCTGCCTCAGC

GCTGATGAAGGCCTTCGAGTCCCTCAAGTCCTTC------CAGCAGCAGCAGCAGCAGCAGCAGCAGCAGCAGCAGCAGCAGCAGCAGCAGCAGCAGCAGCAGCAGCAGCAGCAGCAGCAGCAGCAGCAGCAGCAGCAGCAGCAGCAGCAGCAGCAG**CAACAGCCG**CCACCGCCGCCGCCGCCGCCGCCGCCTCCTCAGCTTCCTCAGCCGCCGCCGCAGGCACAGCCGCTGCTGCCTCAGC 242 1388 DEL

1345 0.0

GCTGATGAAGGCCTTCGAGTCCCT**CAAGTCCTTCC**AGCAGCAGCAGCAGCAGCAGCAGCAGCAGCAGCAGCAGCAGCAGCAGCAGCAGCAGCAGCAGCAGCAGCAGCAGCAGCAGCAGCAGCAGCAGCAGCAGCAGCAGCAGCAGCAGCAGCAGCAGCAG**CAACAGCCG**CCACCGCCGCCGCCGCCGCCGCCGCCTCCTCAGCTTCCTCAGCCGCCGCCGCAGGCACAGCCGCTGCTGCCTCAGC

GCTGATGAAGGCCTTCGAGTCCCTCAAGTCCTTC---CCGCCGCCGCCTCCTCAGCTTCCTCAGCCGCCGCCGCAGGCACAGCCGCTGCTGCCTCAGC 242 1269 DEL

1131 0.0

GCTGATGAAGGCCTTCGAGTCCCT**CAAGTCCTTCC**AGCAGCAGCAGCAGCAGCAGCAGCAGCAGCAGCAGCAGCAGCAGCAGCAGCAGCAGCAGCAGCAGCAGCAGCAGCAGCAGCAGCAGCAGCAGCAGCAGCAGCAGCAGCAGCAGCAGCAGCAGCAG**CAACAGCCG**CCACCGCCGCCGCCGCCGCCGCCGCCTCCTCAGCTTCCTCAGCCGCCGCCGCAGGCACAGCCGCTGCTGCCTCAGC

GCTGATGAAGGCCTTCGAGTCCCTCAAG--GAGAG**CAACAGCCG**CCACCGCCGCCGCCGCCGCCGCCGCCTCCTCAGCTTCCTCAGCCGCCGCCGCAGGCACAGCCGCTGCTGCCTCAGC 242 1259 DEL

1550 0.0

GCTGATGAAGGCCTTCGAGTCCCT**CAAGTCCTTCC**AGCAGCAGCAGCAGCAGCAGCAGCAGCAGCAGCAGCAGCAGCAGCAGCAGCAGCAGCAGCAGCAGCAGCAGCAGCAGCAGCAGCAGCAGCAGCAGCAGCAGCAGCAGCAGCAGCAGCAGCAGCAG**CAACAGCCG**CCACCGCCGCCGCCGCCGCCGCCGCCTCCTCAGCTTCCTCAGCCGCCGCCGCAGGCACAGCCGCTGCTGCCTCAGC

GCTGATGAAGGCCTTCGAGTCCCTCAAGTC--AGCCGCAGGCACAGCCGCTGCTGCCTCAGC 242 1247 DEL

1291 0.0

GCTGATGAAGGCCTTCGAGTCCCT**CAAGTCCTTCC**AGCAGCAGCAGCAGCAGCAGCAGCAGCAGCAGCAGCAGCAGCAGCAGCAGCAGCAGCAGCAGCAGCAGCAGCAGCAGCAGCAGCAGCAGCAGCAGCAGCAGCAGCAGCAGCAGCAGCAGCAGCAG**CAACAGCCG**CCACCGCCGCCGCCGCCGCCGCCGCCTCCTCAGCTTCCTCAGCCGCCGCCGCAGGCACAGCCGCTGCTGCCTCAGC

GCTGATGAAGGCCTTCGAGTCCCT**CAAGTCCTTCC**A---GCCGCCGCCGCCGCCTCCTCAGCTTCCTCAGCCGCCGCCGCAGGCACAGCCGCTGCTGCCTCAGC 242 1200 DEL

1098 0.0

GCTGATGAAGGCCTTCGAGTCCCT**CAAGTCCTTCC**AGCAGCAGCAGCAGCAGCAGCAGCAGCAGCAGCAGCAGCAGCAGCAGCAGCAGCAGCAGCAGCAGCAGCAGCAGCAGCAGCAGCAGCAGCAGCAGCAGCAGCAGCAGCAGCAGCAGCAGCAGCAG**CAACAGCCG**CCACCGCCGCCGCCGCCGCCGCCGCCTCCTCAGCTTCCTCAGCCGCCGCCGCAGGCACAGCCGCTGCTGCCTCAGC

GCTGATGAAGGCCTTCGAGTCCCTCAAGTCACCGCA---AG**CAACAGCCG**CCACCGCCGCCGCCGCCGCCGCCGCCTCCTCAGCTTCCTCAGCCGCCGCCGCAGGCACAGCCGCTGCTGCCTCAGC 242 1197 DEL

1190 0.0

GCTGATGAAGGCCTTCGAGTCCCT**CAAGTCCTTCC**AGCAGCAGCAGCAGCAGCAGCAGCAGCAGCAGCAGCAGCAGCAGCAGCAGCAGCAGCAGCAGCAGCAGCAGCAGCAGCAGCAGCAGCAGCAGCAGCAGCAGCAGCAGCAGCAGCAGCAGCAGCAG**CAACAGCCG**CCACCGCCGCCGCCGCCGCCGCCGCCTCCTCAGCTTCCTCAGCCGCCGCCGCAGGCACAGCCGCTGCTGCCTCAGC

GCTGATGAAGGCCTTCGAGTCCCTCAAGTC--CAGCCGCCACCGCCGCCGCCGCCGCCGCCGCCTCCTCAGCTTCCTCAGCCGCCGCCGCAGGCACAGCCGCTGCTGCCTCAGC 242 1140 DEL

1589 0.0

GCTGATGAAGGCCTTCGAGTCCCT**CAAGTCCTTCC**AGCAGCAGCAGCAGCAGCAGCAGCAGCAGCAGCAGCAGCAGCAGCAGCAGCAGCAGCAGCAGCAGCAGCAGCAGCAGCAGCAGCAGCAGCAGCAGCAGCAGCAGCAGCAGCAGCAGCAGCAGCAG**CAACAGCCG**CCACCGCCGCCGCCGCCGCCGCCGCCTCCTCAGC

GCTGATGAAGGCCTTCGAGTCCCT**CAAGTCCTTCC**A---GCCGCTGCTGCCTCAGC 200 1090 DEL

1502 0.0

GCTGATGAAGGCCTTCGAGTCCCT**CAAGTCCTTCC**AGCAGCAGCAGCAGCAGCAGCAGCAGCAGCAGCAGCAGCAGCAGCAGCAGCAGCAGCAGCAGCAGCAGCAGCAGCAGCAGCAGCAGCAGCAGCAGCAGCAGCAGCAGCAGCAGCAGCAGCAGCAG**CAACAGCCG**CCACCGCCGCCGCCGCCGCCGCCGCCTCCTCAGCTTCCTCAGCCGCCGCCGCAGGCACAGCCGCTGCTGCCTCAGC

GCTGATGAAGGCCTTCGAGTCCCT**CAAGTCCTTCC**---CCGCGGCCGCAGGCACAGCCGCTGCTGCCTCAGC 242 1085 DEL

1404 0.0

GCTGATGAAGGCCTTCGAGTCCCT**CAAGTCCTTCC**AGCAGCAGCAGCAGCAGCAGCAGCAGCAGCAGCAGCAGCAGCAGCAGCAGCAGCAGCAGCAGCAGCAGCAGCAGCAGCAGCAGCAGCAGCAGCAGCAGCAGCAGCAGCAGCAGCAGCAGCAGCAG**CAACAGCCG**CCACCGCCGCCGCCGCCGCCGCCGCCTCCTCAGCTTCCTCAGCCGCCGCCGCAGGCACAGCCGCTGCTGCCTCAGC

GCTGATGAAGGCCTTCGAGTCCCTCAAGTCCTT---CGCCTCCTCAGCTTCCTCAGCCGCCGCCGCAGGCACAGCCGCTGCTGCCTCAGC 242 1004 DEL

273 36.0

GCTGATGAAGGCCTTCGAGTCCCT**CAAGTCCTTCC**AGCAGCAGCAGCAGCAGCAGCAGCAGCAGCAGCAGCAGCAGCAGCAGCAGCAGCAGCAGCAGCAGCAGCAGCAGCAGCAGCAGCAGCAGCAGCAGCAGCAGCAGCAGCAGCAGCAGCAGCAGCAG**CAACAGCCG**CCACCGCCGCCGCCGCCGCCGCCGCCTCCTCAGCTTCCTCAGCCGCCGCCGCAGGCACAGCCGCTGCTGCCTCAGC

GCTGATGAAGGCCTTCGAGTCCCT**CAAGTCCTTCC**A-----------AGCAGCAGCAGCAGCAGCAGCAGCAGCAGCAGCAGCAGCAGCAGCAGCAGCAGCAGCAGCAGCAGCAGCAGCAGCAGCAGCAGCAGCAGCAGCAGCAGCAGCAGCAGCAGCAG**CAACAGCCG**CCACCGCCGCCGCCGCCGCCGCCGCCTCCTCAGCTTCCTCAGCCGCCGCCGCAGGCACAGCCGCTGCTGCCTCAGC 242 984 DEL

155 39.0

GCTGATGAAGGCCTTCGAGTCCCT**CAAGTCCTTCC**AGCAGCAGCAGCAGCAGCAGCAGCAGCAGCAGCAGCAGCAGCAGCAGCAGCAGCAGCAGCAGCAGCAGCAGCAGCAGCAGCAGCAGCAGCAGCAGCAGCAGCAGCAGCAGCAGCAGCAGCAGCAG**CAACAGCCG**CCACCGCCGCCGCCGCCGCCGCCGCCTCCTCAGCTTCCTCAGCCGCCGCCGCAGGCACAGCCGCTGCTGCCTCAGC

GCTGATGAAGGCCTTCGAGTCCCT**CAAGTCCTTCC**A----CAGCAGCAGCAGCAGCAGCAGCAGCAGCAGCAGCAGCAGCAGCAGCAGCAGCAGCAGCAGCAGCAGCAGCAGCAGCAGCAGCAGCAGCAGCAGCAGCAGCAGCAGCAGCAGCAGCAGCAG**CAACAGCCG**CCACCGCCGCCGCCGCCGCCGCCGCCTCCTCAGCTTCCTCAGCCGCCGCCGCAGGCACAGCCGCTGCTGCCTCAGC 242 970 DEL

1171 0.0

GCTGATGAAGGCCTTCGAGTCCCT**CAAGTCCTTCC**AGCAGCAGCAGCAGCAGCAGCAGCAGCAGCAGCAGCAGCAGCAGCAGCAGCAGCAGCAGCAGCAGCAGCAGCAGCAGCAGCAGCAGCAGCAGCAGCAGCAGCAGCAGCAGCAGCAGCAGCAGCAG**CAACAGCCG**CCACCGCCGCCGCCGCCGCCGCCGCCTCCTCAGCTTCCTCAGCCGCCGCCGCAGGCACAGCCGCTGCTGCCTCAGC

GCTGATGAAGGCCTTCGAGTCCCT**CAAGTCCTTCC**A--AGCCACCGCCGCCGCCGCCGCCGCCGCCTCCTCAGCTTCCTCAGCCGCCGCCGCAGGCACAGCCGCTGCTGCCTCAGC 242 948 DEL

1576 0.0

GCTGATGAAGGCCTTCGAGTCCCT**CAAGTCCTTCC**AGCAGCAGCAGCAGCAGCAGCAGCAGCAGCAGCAGCAGCAGCAGCAGCAGCAGCAGCAGCAGCAGCAGCAGCAGCAGCAGCAGCAGCAGCAGCAGCAGCAGCAGCAGCAGCAGCAGCAGCAGCAG**CAACAGCCG**CCACCGCCGCCGCCGCCGCCGCCGCCTCCTCAGC

GCTGATGAAGGCCTTCGAGTCCCTCAAGTCCTT---CCGCCGCCGCCGCCGCCTCAGC 200 945 DEL

74 42.0

GCTGATGAAGGCCTTCGAGTCCCTCAAGTCCTTC-------CAGCAGCAGCAGCAGCAGCAGCAGCAGCAGCAGCAGCAGCAGCAGCAGCAGCAGCAGCAGCAGCAGCAGCAGCAGCAGCAGCAGCAGCAGCAGCAGCAGCAGCAGCAGCAGCAGCAGCAGCAG**CAACAGCCG**CCACCGCCGCCGCCGCCGCCGCCGCCTCCTCAGCTTCCTCAGCCGCCGCCGCAGGCACAGCCGCTGCTGCCTCAGC

GCTGATGAAGGCCTTCGAGTCCCT**CAAGTCCTTCC**AAGCAGCAGCAGCAGCAGCAGCAGCAGCAGCAGCAGCAGCAGCAGCAGCAGCAGCAGCAGCAGCAGCAGCAGCAGCAGCAGCAGCAGCAGCAGCAGCAGCAGCAGCAGCAGCAGCAGCAGCAGCAGCAGCAG**CAACAGCCG**CCACCGCCGCCGCCGCCGCCGCCGCCTCCTCAGCTTCCTCAGCCGCCGCCGCAGGCACAGCCGCTGCTGCCTCAGC 242 940 INS

1318 0.0

GCTGATGAAGGCCTTCGAGTCCCT**CAAGTCCTTCC**AGCAGCAGCAGCAGCAGCAGCAGCAGCAGCAGCAGCAGCAGCAGCAGCAGCAGCAGCAGCAGCAGCAGCAGCAGCAGCAGCAGCAGCAGCAGCAGCAGCAGCAGCAGCAGCAGCAGCAGCAGCAG**CAACAGCCG**CCACCGCCGCCGCCGCCGCCGCCGCCTCCTCAGCTTCCTCAGCCGCCGCCGCAGGCACAGCCGCTGCTGCCTCAGC

GCTGATGAAGGCCTTCGAGTCCCT**CAAGTCCTTCC**A--GCCGCTGCTGCCTCCTCAGCTTCCTCAGCCGCCGCCGCAGGCACAGCCGCTGCTGCCTCAGC 242 930 DEL

1067 0.0

GCTGATGAAGGCCTTCGAGTCCCT**CAAGTCCTTCC**AGCAGCAGCAGCAGCAGCAGCAGCAGCAGCAGCAGCAGCAGCAGCAGCAGCAGCAGCAGCAGCAGCAGCAGCAGCAGCAGCAGCAGCAGCAGCAGCAGCAGCAGCAGCAGCAGCAGCAGCAGCAG**CAACAGCCG**CCACCGCCGCCGCCGCCGCCGCCGCCTCCTCAGCTTCCTCAGCCGCCGCCGCAGGCACAGCCGCTGCTGCCTCAGC

GCTGATGAAGGCCTTCGAGTCCCTCAAGTCCTT--GAGGTGGTGG**CAACAGCCG**CCACCGCCGCCGCCGCCGCCGCCGCCTCCTCAGCTTCCTCAGCCGCCGCCGCAGGCACAGCCGCTGCTGCCTCAGC 242 881 DEL

256 39.0

GCTGATGAAGGCCTTCGAGTCCCT**CAAGTCCTTCC**AGCAGCAGCAGCAGCAGCAGCAGCAGCAGCAGCAGCAGCAGCAGCAGCAGCAGCAGCAGCAGCAGCAGCAGCAGCAGCAGCAGCAGCAGCAGCAGCAGCAGCAGCAGCAGCAGCAGCAGCAGCAG**CAACAGCCG**CCACCGCCGCCGCCGCCGCCGCCGCCTCCTCAGCTTCCTCAGCCGCCGCCGCAGGCACAGCCGCTGCTGCCTCAGC

GCTGATGAAGGCCTTCGAGTCCCTCAAGT---------AGCAGCAGCAGCAGCAGCAGCAGCAGCAGCAGCAGCAGCAGCAGCAGCAGCAGCAGCAGCAGCAGCAGCAGCAGCAGCAGCAGCAGCAGCAGCAGCAGCAGCAGCAGCAGCAGCAGCAGCAG**CAACAGCCG**CCACCGCCGCCGCCGCCGCCGCCGCCTCCTCAGCTTCCTCAGCCGCCGCCGCAGGCACAGCCGCTGCTGCCTCAGC 242 855 DEL

1161 0.0

GCTGATGAAGGCCTTCGAGTCCCT**CAAGTCCTTCC**AGCAGCAGCAGCAGCAGCAGCAGCAGCAGCAGCAGCAGCAGCAGCAGCAGCAGCAGCAGCAGCAGCAGCAGCAGCAGCAGCAGCAGCAGCAGCAGCAGCAGCAGCAGCAGCAGCAGCAGCAGCAG**CAACAGCCG**CCACCGCCGCCGCCGCCGCCGCCGCCTCCTCAGCTTCCTCAGCCGCCGCCGCAGGCACAGCCGCTGCTGCCTCAGC

GCTGATGAAGGCCTTCGAGTCCCT---CAGCAG**CAACAGCCG**CCACCGCCGCCGCCGCCGCCGCCGCCTCCTCAGCTTCCTCAGCCGCCGCCGCAGGCACAGCCGCTGCTGCCTCAGC 242 844 DEL

1088 0.0

GCTGATGAAGGCCTTCGAGTCCCT**CAAGTCCTTCC**AGCAGCAGCAGCAGCAGCAGCAGCAGCAGCAGCAGCAGCAGCAGCAGCAGCAGCAGCAGCAGCAGCAGCAGCAGCAGCAGCAGCAGCAGCAGCAGCAGCAGCAGCAGCAGCAGCAGCAGCAGCAG**CAACAGCCG**CCACCGCCGCCGCCGCCGCCGCCGCCTCCTCAGCTTCCTCAGCCGCCGCCGCAGGCACAGCCGCTGCTGCCTCAGC

GCTGATGAAGGCCTTCGAGTCCCTCAAGTCCTTC---CAGCAG**CAACAGCCG**CCACCGCCGCCGCCGCCGCCGCCGCCTCCTCAGCTTCCTCAGCCGCCGCCGCAGGCACAGCCGCTGCTGCCTCAGC 242 750 DEL

1383 0.0

GCTGATGAAGGCCTTCGAGTCCCT**CAAGTCCTTCC**AGCAGCAGCAGCAGCAGCAGCAGCAGCAGCAGCAGCAGCAGCAGCAGCAGCAGCAGCAGCAGCAGCAGCAGCAGCAGCAGCAGCAGCAGCAGCAGCAGCAGCAGCAGCAGCAGCAGCAGCAGCAG**CAACAGCCG**CCACCGCCGCCGCCGCCGCCGCCGCCTCCTCAGCTTCCTCAGCCGCCGCCGCAGGCACAGCCGCTGCTGCCTCAGC

GCTGATGAAGGCCTTCGAGTCCCTCAAGT--CCGCCGCCGCCTCCTCAGCTTCCTCAGCCGCCGCCGCAGGCACAGCCGCTGCTGCCTCAGC 242 747 DEL

1183 0.0

GCTGATGAAGGCCTTCGAGTCCCT**CAAGTCCTTCC**AGCAGCAGCAGCAGCAGCAGCAGCAGCAGCAGCAGCAGCAGCAGCAGCAGCAGCAGCAGCAGCAGCAGCAGCAGCAGCAGCAGCAGCAGCAGCAGCAGCAGCAGCAGCAGCAGCAGCAGCAGCAG**CAACAGCCG**CCACCGCCGCCGCCGCCGCCGCCGCCTCCTCAGCTTCCTCAGCCGCCGCCGCAGGCACAGCCGCTGCTGCCTCAGC

GCTGATGAAGGCCTTCGAGTCCCT**CAAGTCCTTCC**A---GCCACCGCCGCCGCCGCCGCCGCCGCCTCCTCAGCTTCCTCAGCCGCCGCCGCAGGCACAGCCGCTGCTGCCTCAGC 242 723 DEL

1267 0.0

GCTGATGAAGGCCTTCGAGTCCCT**CAAGTCCTTCC**AGCAGCAGCAGCAGCAGCAGCAGCAGCAGCAGCAGCAGCAGCAGCAGCAGCAGCAGCAGCAGCAGCAGCAGCAGCAGCAGCAGCAGCAGCAGCAGCAGCAGCAGCAGCAGCAGCAGCAGCAGCAG**CAACAGCCG**CCACCGCCGCCGCCGCCGCCGCCGCCTCCTCAGCTTCCTCAGCCGCCGCCGCAGGCACAGCCGCTGCTGCCTCAGC

GCTGATGAAGGCCTTCGAGTCCCTCAAGTCCTTCAA--GCCGCCGCCGCCGCCGCCTCCTCAGCTTCCTCAGCCGCCGCCGCAGGCACAGCCGCTGCTGCCTCAGC 242 687 DEL

1050 0.0

GCTGATGAAGGCCTTCGAGTCCCTCAAGTCCT----TCCAGCAGCAGCAGCAGCAGCAGCAGCAGCAGCAGCAGCAGCAGCAGCAGCAGCAGCAGCAGCAGCAGCAGCAGCAGCAGCAGCAGCAGCAGCAGCAGCAGCAGCAGCAGCAGCAGCAGCAGCAG**CAACAGCCG**CCACCGCCGCCGCCGCCGCCGCCGCCTCCTCAGCTTCCTCAGCCGCCGCCGCAGGCACAGCCGCTGCTGCCTCAGC

GCTGATGAAGGCCTTCGAGTCCCTCAAGTCCTCAAGTCCCTCA--AG**CAACAGCCG**CCACCGCCGCCGCCGCCGCCGCCGCCTCCTCAGCTTCCTCAGCCGCCGCCGCAGGCACAGCCGCTGCTGCCTCAGC 242 669 INS+DEL

1234 0.0

GCTGATGAAGGCCTTCGAGTCCCT**CAAGTCCTTCC**AGCAGCAGCAGCAGCAGCAGCAGCAGCAGCAGCAGCAGCAGCAGCAGCAGCAGCAGCAGCAGCAGCAGCAGCAGCAGCAGCAGCAGCAGCAGCAGCAGCAGCAGCAGCAGCAGCAGCAGCAGCAG**CAACAGCCG**CCACCGCCGCCGCCGCCGCCGCCGCCTCCTCAGCTTCCTCAGCCGCCGCCGCAGGCACAGCCGCTGCTGCCTCAGC

GCTGATGAAGGCCTTCGAGTCCCT**CAAGTCCTTCC**A---GCCGCCGCCGCCGCCGCCGCCTCCTCAGCTTCCTCAGCCGCCGCCGCAGGCACAGCCGCTGCTGCCTCAGC 242 665 DEL

1608 0.0

GCTGATGAAGGCCTTCGAGTCCCT**CAAGTCCTTCC**AGCAGCAGCAGCAGCAGCAGCAGCAGCAGCAGCAGCAGCAGCAGCAGCAGCAGCAGCAGCAGCAGCAGCAGCAGCAGCAGCAGCAGCAGCAGCAGCAGCAGCAGCAGCAGCAGCAGCAGCAGCAG**CAACAGCCG**CCACCGCCGCCGCCGCCGCCGCCGCCTCCTCAGCTTCCTCAGCCGCCGCCGCAGGCACAGCCGCTGCTGCCTCAGC

GCTGATGAAGGCCTTCGAGTC---CCGCAGGCACAGCCGCTGCTGCCTCAGC 242 647 DEL

796 15.0

GCTGATGAAGGCCTTCGAGTCCCT**CAAGTCCTTCC**AGCAGCAGCAGCAGCAGCAGCAGCAGCAGCAGCAGCAGCAGCAGCAGCAGCAGCAGCAGCAGCAGCAGCAGCAGCAGCAGCAGCAGCAGCAGCAGCAGCAGCAGCAGCAGCAGCAGCAGCAGCAG**CAACAGCCG**CCACCGCCGCCGCCGCCGCCGCCGCCTCCTCAGCTTCCTCAGCCGCCGCCGCAGGCACAGCCGCTGCTGCCTCAGC

GCTGATGAAGGCCTTCGAGTCCCT**CAAGTCCTTCC**A--AGCAGCAGCAGCAGCAGCAGCAGCAGCAGCAGCAGCAGCAGCAGCAGCAG**CAACAGCCG**CCACCGCCGCCGCCGCCGCCGCCGCCTCCTCAGCTTCCTCAGCCGCCGCCGCAGGCACAGCCGCTGCTGCCTCAGC 242 641 DEL

102 42.0

GCTGATGAAGGCCTTCGAGTCCCTCAAGTCCTTC---CAGCAGCAGCAGCAGCAGCAGCAGCAGCAGCAGCAGCAGCAGCAGCAGCAGCAGCAGCAGCAGCAGCAGCAGCAGCAGCAGCAGCAGCAGCAGCAGCAGCAGCAGCAGCAGCAGCAGCAGCAGCAG**CAACAGCCG**CCACCGCCGCCGCCGCCGCCGCCGCCTCCTCAGCTTCCTCAGCCGCCGCCGCAGGCACAGCCGCTGCTGCCTCAGC

GCTGATGAAGGCCTTCGAGTCCCT**CAAGTCCTTCC**AGCAGCAGCAGCAGCAGCAGCAGCAGCAGCAGCAGCAGCAGCAGCAGCAGCAGCAGCAGCAGCAGCAGCAGCAGCAGCAGCAGCAGCAGCAGCAGCAGCAGCAGCAGCAGCAGCAGCAGCAGCAGCAG**CAACAGCCG**CCACCGCCGCCGCCGCCGCCGCCGCCTCCTCAGCTTCCTCAGCCGCCGCCGCAGGCACAGCCGCTGCTGCCTCAGC 242 632 INS

1489 0.0

GCTGATGAAGGCCTTCGAGTCCCT**CAAGTCCTTCC**AGCAGCAGCAGCAGCAGCAGCAGCAGCAGCAGCAGCAGCAGCAGCAGCAGCAGCAGCAGCAGCAGCAGCAGCAGCAGCAGCAGCAGCAGCAGCAGCAGCAGCAGCAGCAGCAGCAGCAGCAGCAG**CAACAGCCG**CCACCGCCGCCGCCGCCGCCGCCGCCTCCTCAGCTTCCTCAGCCGCCGCCGCAGGCACAGCCGCTGCTGCCTCAGC

GCTGATGAAGGCCTTCGAGTCCCTCA---AGCTTCCTCAGCCGCCGCCGCAGGCACAGCCGCTGCTGCCTCAGC 242 630 DEL

900 13.0

GCTGATGAAGGCCTTCGAGTCCCT**CAAGTCCTTCC**AGCAGCAGCAGCAGCAGCAGCAGCAGCAGCAGCAGCAGCAGCAGCAGCAGCAGCAGCAGCAGCAGCAGCAGCAGCAGCAGCAGCAGCAGCAGCAGCAGCAGCAGCAGCAGCAGCAGCAGCAGCAG**CAACAGCCG**CCACCGCCGCCGCCGCCGCCGCCGCCTCCTCAGCTTCCTCAGCCGCCGCCGCAGGCACAGCCGCTGCTGCCTCAGC

GCTGATGAAGGCCTTCGAGTCCCTCAAGTC--CAGCAGCAGCAGCAGCAGCAGCAGCAGCAGCAGCAGCAG**CAACAGCCG**CCACCGCCGCCGCCGCCGCCGCCGCCTCCTCAGCTTCCTCAGCCGCCGCCGCAGGCACAGCCGCTGCTGCCTCAGC 242 624 DEL

1152 0.0

GCTGATGAAGGCCTTCGAGTCCCT**CAAGTCCTTCC**AGCAGCAGCAGCAGCAGCAGCAGCAGCAGCAGCAGCAGCAGCAGCAGCAGCAGCAGCAGCAGCAGCAGCAGCAGCAGCAGCAGCAGCAGCAGCAGCAGCAGCAGCAGCAGCAGCAGCAGCAGCAG**CAACAGCCG**CCACCGCCGCCGCCGCCGCCGCCGCCTCCTCAGCTTCCTCAGCCGCCGCCGCAGGCACAGCCGCTGCTGCCTCAGC

GCTGATGAAGGCCTTCGAGTCCCTCAAGTCCTTC--CAGCCGCCACCGCCGCCGCCGCCGCCGCCGCCTCCTCAGCTTCCTCAGCCGCCGCCGCAGGCACAGCCGCTGCTGCCTCAGC 242 622 DEL

222 38.0

GCTGATGAAGGCCTTCGAGTCCCT**CAAGTCCTTCC**AGCAGCAGCAGCAGCAGCAGCAGCAGCAGCAGCAGCAGCAGCAGCAGCAGCAGCAGCAGCAGCAGCAGCAGCAGCAGCAGCAGCAGCAGCAGCAGCAGCAGCAGCAGCAGCAGCAGCAGCAGCAG**CAACAGCCG**CCACCGCCGCCGCCGCCGCCGCCGCCTCCTCAGCTTCCTCAGCCGCCGCCGCAGGCACAGCCGCTGCTGCCTCAGC

GCTGATGAAGGCCTTCGAGTCCCT**CAAGTCCTTCC**A-------CAGCAGCAGCAGCAGCAGCAGCAGCAGCAGCAGCAGCAGCAGCAGCAGCAGCAGCAGCAGCAGCAGCAGCAGCAGCAGCAGCAGCAGCAGCAGCAGCAGCAGCAGCAG**CAACAGCCG**CCACCGCCGCCGCCGCCGCCGCCGCCTCCTCAGCTTCCTCAGCCGCCGCCGCAGGCACAGCCGCTGCTGCCTCAGC 242 621 DEL

1126 2.0

GCTGATGAAGGCCTTCGAGTCCCT**CAAGTCCTTCC**AGCAGCAGCAGCAGCAGCAGCAGCAGCAGCAGCAGCAGCAGCAGCAGCAGCAGCAGCAGCAGCAGCAGCAGCAGCAGCAGCAGCAGCAGCAGCAGCAGCAGCAGCAGCAGCAGCAGCAGCAGCAG**CAACAGCCG**CCACCGCCGCCGCCGCCGCCGCCGCCTCCTCAGCTTCCTCAGCCGCCGCCGCAGGCACAGCCGCTGCTGCCTCAGC

GCTGATGAAGGCCTTCGAGTCCCTCA---AGCAGCAG**CAACAGCCG**CCACCGCCGCCGCCGCCGCCGCCGCCTCCTCAGCTTCCTCAGCCGCCGCCGCAGGCACAGCCGCTGCTGCCTCAGC 242 616 DEL

1080 4.0

GCTGATGAAGGCCTTCGAGTCCCT**CAAGTCCTTCC**AGCAGCAGCAGCAGCAGCAGCAGCAGCAGCAGCAGCAGCAGCAGCAGCAGCAGCAGCAGCAGCAGCAGCAGCAGCAGCAGCAGCAGCAGCAGCAGCAGCAGCAGCAGCAGCAGCAGCAGCAGCAG**CAACAGCCG**CCACCGCCGCCGCCGCCGCCGCCGCCTCCTCAGCTTCCTCAGCCGCCGCCGCAGGCACAGCCGCTGCTGCCTCAGC

GCTGATGAAGGCCTTCGAGTCCCTCAAGT--CAGCAGCAGCAG**CAACAGCCG**CCACCGCCGCCGCCGCCGCCGCCGCCTCCTCAGCTTCCTCAGCCGCCGCCGCAGGCACAGCCGCTGCTGCCTCAGC 242 610 DEL

1162 0.0

GCTGATGAAGGCCTTCGAGTCCCT**CAAGTCCTTCC**AGCAGCAGCAGCAGCAGCAGCAGCAGCAGCAGCAGCAGCAGCAGCAGCAGCAGCAGCAGCAGCAGCAGCAGCAGCAGCAGCAGCAGCAGCAGCAGCAGCAGCAGCAGCAGCAGCAGCAGCAGCAG**CAACAGCCG**CCACCGCCGCCGCCGCCGCCGCCGCCTCCTCAGCTTCCTCAGCCGCCGCCGCAGGCACAGCCGCTGCTGCCTCAGC

GCTGATGAAGGCCTTCGAGTCCCT**CAAGTCCTTCC**---GCCGCCACCGCCGCCGCCGCCGCCGCCGCCTCCTCAGCTTCCTCAGCCGCCGCCGCAGGCACAGCCGCTGCTGCCTCAGC 242 606 DEL

439 33.0
GCTGATGAAGGCCTTCGAGTCCCT**CAAGTCCTTCC**AGCAGCAGCAGCAGCAGCAGCAGCAGCAGCAGCAGCAGCAGCAGCAGCAGCAGCAGCAGCAGCAGCAGCAGCAGCAGCAGCAGCAGCAGCAGCAGCAGCAGCAGCAGCAGCAGCAGCAGCAGCAG**CAACAGCCG**CCACCGCCGCCGCCGCCGCCGCCTCCTCAGCTTCCTCAGCCGCCGCGCAGGCACAGCCGCTGCTGCCTCAGC
GCTGATGAAGGCCTTCGAGTCCCTCAAGT-----------------------------CAGCAGCAGCAGCAGCAGCAGCAGCAGCAGCAGCAGCAGCAGCAGCAGCAGCAGCAGCAGCAGCAGCAGCAGCAGCAGCAGCAGCAGCAGCAGCAGCAGCAG**CAACAGCCG**CCACCGCCGCCGCCGCCGCCGCCTCCTCAGCTTCCTCAGCCGCCGCGCAGGCACAGCCGCTGCTGCCTCAGC 242 590 DEL
1208 0.0
GCTGATGAAGGCCTTCGAGTCCCT**CAAGTCCTTCC**AGCAGCAGCAGCAGCAGCAGCAGCAGCAGCAGCAGCAGCAGCAGCAGCAGCAGCAGCAGCAGCAGCAGCAGCAGCAGCAGCAGCAGCAGCAGCAGCAGCAGCAGCAGCAGCAGCAGCAGCAGCAG**CAACAGCCG**CCACCGCCGCCGCCGCCGCCGCCTCCTCAGCTTCCTCAGCCGCCGCGCAGGCACAGCCGCTGCTGCCTCAGC
GCTGATGAAGGCCTTCGAGTCCCT**CAAGTCCTTCC**A--ACCGCGGCCGCCGCCGCCGCCTCCTCAGCTTCCTCAGCCGCCGCGCAGGCACAGCCGCTGCTGCCTCAGC 242 568 DEL
715 22.0
GCTGATGAAGGCCTTCGAGTCCCT**CAAGTCCTTCC**AGCAGCAGCAGCAGCAGCAGCAGCAGCAGCAGCAGCAGCAGCAGCAGCAGCAGCAGCAGCAGCAGCAGCAGCAGCAGCAGCAGCAGCAGCAGCAGCAGCAGCAGCAGCAGCAGCAGCAGCAGCAG**CAACAGCCG**CCACCGCCGCCGCCGCCGCCGCCTCCTCAGCTTCCTCAGCCGCCGCGCAGGCACAGCCGCTGCTGCCTCAGC
GCTGATGAAGGCCTTCGAGTCCCTCA---AGCAGCAGCAGCAGCAGCAGCAGCAGCAGCAGCAGCAGCAGCAGCAGCAGCAGCAGCAGCAGCAGCAGCAG**CAACAGCCG**CCACCGCCGCCGCCGCCGCCGCCTCCTCAGCTTCCTCAGCCGCCGCGCAGGCACAGCCGCTGCTGCCTCAGC 242 546 DEL
88 42.0
GCTGATGAAGGCCTTCGAGTCCCTCAAGTCCTTC-----CAGCAGCAGCAGCAGCAGCAGCAGCAGCAGCAGCAGCAGCAGCAGCAGCAGCAGCAGCAGCAGCAGCAGCAGCAGCAGCAGCAGCAGCAGCAGCAGCAGCAGCAGCAGCAGCAGCAGCAGCAGCAG**CAACAGCCG**CCACCGCCGCCGCCGCCGCCGCCTCCTCAGCTTCCTCAGCCGCCGCGCAGGCACAGCCGCTGCTGCCTCAGC
GCTGATGAAGGCCTTCGAGTCCCT**CAAGTCCTTCC**ACAGCAGCAGCAGCAGCAGCAGCAGCAGCAGCAGCAGCAGCAGCAGCAGCAGCAGCAGCAGCAGCAGCAGCAGCAGCAGCAGCAGCAGCAGCAGCAGCAGCAGCAGCAGCAGCAGCAGCAGCAGCAGCAG**CAACAGCCG**CCACCGCCGCCGCCGCCGCCGCCTCCTCAGCTTCCTCAGCCGCCGCGCAGGCACAGCCGCTGCTGCCTCAGC 242 523 INS
1433 0.0
GCTGATGAAGGCCTTCGAGTCCCT**CAAGTCCTTCC**AGCAGCAGCAGCAGCAGCAGCAGCAGCAGCAGCAGCAGCAGCAGCAGCAGCAGCAGCAGCAGCAGCAGCAGCAGCAGCAGCAGCAGCAGCAGCAGCAGCAGCAGCAGCAGCAGCAGCAGCAGCAG**CAACAGCCG**CCACCGCCGCCGCCGCCGCCGCCTCCTCAGCTTCCTCAGCCGCCGCGCAGGCACAGCCGCTGCTGCCTCAGC
GCTGATGAAGGCCTTCGAGTCC--CCGCCGCCGCCTCCTCAGCTTCCTCAGCCGCCGCGCAGGCACAGCCGCTGCTGCCTCAGC 242 520 DEL
859 12.0
GCTGATGAAGGCCTTCGAGTCCCT**CAAGTCCTTCC**AGCAGCAGCAGCAGCAGCAGCAGCAGCAGCAGCAGCAGCAGCAGCAGCAGCAGCAGCAGCAGCAGCAGCAGCAGCAGCAGCAGCAGCAGCAGCAGCAGCAGCAGCAGCAGCAGCAGCAGCAGCAG**CAACAGCCG**CCACCGCCGCCGCCGCCGCCGCCTCCTCAGCTTCCTCAGCCGCCGCGCAGGCACAGCCGCTGCTGCCTCAGC
GCTGATGAAGGCCTTCGAGTCCCT**CAAGTCCTTCC**A---AGCAGCAGCAGCAGCAGCAGCAGCAGCAGCAGCAGCAG**CAACAGCCG**CCACCGCCGCCGCCGCCGCCGCCTCCTCAGCTTCCTCAGCCGCCGCGCAGGCACAGCCGCTGCTGCCTCAGC 242 509 DEL
1292 0.0
GCTGATGAAGGCCTTCGAGTCCCT**CAAGTCCTTCC**AGCAGCAGCAGCAGCAGCAGCAGCAGCAGCAGCAGCAGCAGCAGCAGCAGCAGCAGCAGCAGCAGCAGCAGCAGCAGCAGCAGCAGCAGCAGCAGCAGCAGCAGCAGCAGCAGCAGCAGCAGCAG**CAACAGCCG**CCACCGCCGCCGCCGCCGCCGCCTCCTCAGCTTCCTCAGCCGCCGCGCAGGCACAGCCGCTGCTGCCTCAGC
GCTGATGAAGGCCTTCGAGTCCCTCAA---ACCGCCGCCGCCGCCGCCGCCTCCTCAGCTTCCTCAGCCGCCGCGCAGGCACAGCCGCTGCTGCCTCAGC 242 464 DEL
1470 0.0
GCTGATGAAGGCCTTCGAGTCCCT**CAAGTCCTTCC**AGCAGCAGCAGCAGCAGCAGCAGCAGCAGCAGCAGCAGCAGCAGCAGCAGCAGCAGCAGCAGCAGCAGCAGCAGCAGCAGCAGCAGCAGCAGCAGCAGCAGCAGCAGCAGCAGCAGCAGCAGCAG**CAACAGCCG**CCACCGCCGCCGCCGCCGCCGCCTCCTCAGCTTCCTCAGCCGCCGCGCAGGCACAGCCGCTGCTGCCTCAGC
GCTGATGAAGGCCTTCGAGTCC---CCTCCTCAGCTTCCTCAGCCGCCGCGCAGGCACAGCCGCTGCTGCCTCAGC 242 458 DEL
1244 0.0
GCTGATGAAGGCCTTCGAGTCCCT**CAAGTCCTTCC**AGCAGCAGCAGCAGCAGCAGCAGCAGCAGCAGCAGCAGCAGCAGCAGCAGCAGCAGCAGCAGCAGCAGCAGCAGCAGCAGCAGCAGCAGCAGCAGCAGCAGCAGCAGCAGCAGCAGCAGCAGCAG**CAACAGCCG**CCACCGCCGCCGCCGCCGCCGCCTCCTCAGCTTCCTCAGCCGCCGCGCAGGCACAGCCGCTGCTGCCTCAGC
GCTGATGAAGGCCTTCGAGTCC---ACAGCCGCCACCGCCGCCGCCGCCGCCGCCTCCTCAGCTTCCTCAGCCGCCGCGCAGGCACAGCCGCTGCTGCCTCAGC 242 456 DEL
308 35.0
GCTGATGAAGGCCTTCGAGTCCCT**CAAGTCCTTCC**AGCAGCAGCAGCAGCAGCAGCAGCAGCAGCAGCAGCAGCAGCAGCAGCAGCAGCAGCAGCAGCAGCAGCAGCAGCAGCAGCAGCAGCAGCAGCAGCAGCAGCAGCAGCAGCAGCAGCAGCAGCAG**CAACAGCCG**CCACCGCCGCCGCCGCCGCCGCCTCCTCAGCTTCCTCAGCCGCCGCGCAGGCACAGCCGCTGCTGCCTCAGC
GCTGATGAAGGCCTTCGAGTCCCT**CAAGTCCTTCC**A--------------AGCAGCAGCAGCAGCAGCAGCAGCAGCAGCAGCAGCAGCAGCAGCAGCAGCAGCAGCAGCAGCAGCAGCAGCAGCAGCAGCAGCAGCAGCAGCAGCAGCAGCAGCAG**CAACAGCCG**CCACCGCCGCCGCCGCCGCCGCCTCCTCAGCTTCCTCAGCCGCCGCGCAGGCACAGCCGCTGCTGCCTCAGC 242 451 DEL
266 41.0
GCTGATGAAGGCCTTCGAGTCCCT**CAAGTCCTTCC**AGCAGCAGCAGCAGCAGCAGCAGCAGCAGCAGCAGCAGCAGCAGCAGCAGCAGCAGCAGCAGCAGCAGCAGCAGCAGCAGCAGCAGCAGCAGCAGCAGCAGCAGCAGCAGCAGCAGCAGCAGCAG**CAACAGCCG**CCACCGCCGCCGCCGCCGCCGCCTCCTCAGCTTCCTCAGCCGCCGCGCAGGCACAGCCGCTGCTGCCTCAGC
GCTGATGAAGGCCTTCGAGTCCCT----------CAGCAGCAGCAGCAGCAGCAGCAGCAGCAGCAGCAGCAGCAGCAGCAGCAGCAGCAGCAGCAGCAGCAGCAGCAGCAGCAGCAGCAGCAGCAGCAGCAGCAGCAGCAGCAGCAGCAGCAGCAGCAG**CAACAGCCG**CCACCGCCGCCGCCGCCGCCGCCTCCTCAGCTTCCTCAGCCGCCGCGCAGGCACAGCCGCTGCTGCCTCAGC 242 442 DEL
771 16.0
GCTGATGAAGGCCTTCGAGTCCCT**CAAGTCCTTCC**AGCAGCAGCAGCAGCAGCAGCAGCAGCAGCAGCAGCAGCAGCAGCAGCAGCAGCAGCAGCAGCAGCAGCAGCAGCAGCAGCAGCAGCAGCAGCAGCAGCAGCAGCAGCAGCAGCAGCAGCAGCAG**CAACAGCCG**CCACCGCCGCCGCCGCCGCCGCCTCCTCAGCTTCCTCAGCCGCCGCGCAGGCACAGCCGCTGCTGCCTCAGC
GCTGATGAAGGCCTTCGAGTCCCT**CAAGTCCTTCC**A---AGCAGCAGCAGCAGCAGCAGCAGCAGCAGCAGCAGCAGCAGCAGCAGCAG**CAACAGCCG**CCACCGCCGCCGCCGCCGCCGCCTCCTCAGCTTCCTCAGCCGCCGCGCAGGCACAGCCGCTGCTGCCTCAGC 242 428 DEL
301 36.0
GCTGATGAAGGCCTTCGAGTCCCT**CAAGTCCTTCC**AGCAGCAGCAGCAGCAGCAGCAGCAGCAGCAGCAGCAGCAGCAGCAGCAGCAGCAGCAGCAGCAGCAGCAGCAGCAGCAGCAGCAGCAGCAGCAGCAGCAGCAGCAGCAGCAGCAGCAGCAGCAG**CAACAGCCG**CCACCGCCGCCGCCGCCGCCGCCTCCTCAGCTTCCTCAGCCGCCGCGCAGGCACAGCCGCTGCTGCCTCAGC
GCTGATGAAGGCCTTCGAGTCCCT**CAAGTCCTTCC**A-------------CAGCAGCAGCAGCAGCAGCAGCAGCAGCAGCAGCAGCAGCAGCAGCAGCAGCAGCAGCAGCAGCAGCAGCAGCAGCAGCAGCAGCAGCAGCAGCAGCAGCAGCAGCAG**CAACAGCCG**CCACCGCCGCCGCCGCCGCCGCCTCCTCAGCTTCCTCAGCCGCCGCGCAGGCACAGCCGCTGCTGCCTCAGC 242 422 DEL
414 31.0
GCTGATGAAGGCCTTCGAGTCCCT**CAAGTCCTTCC**AGCAGCAGCAGCAGCAGCAGCAGCAGCAGCAGCAGCAGCAGCAGCAGCAGCAGCAGCAGCAGCAGCAGCAGCAGCAGCAGCAGCAGCAGCAGCAGCAGCAGCAGCAGCAGCAGCAGCAGCAGCAG**CAACAGCCG**CCACCGCCGCCGCCGCCGCCGCCTCCTCAGCTTCCTCAGCCGCCGCGCAGGCACAGCCGCTGCTGCCTCAGC
GCTGATGAAGGCCTTCGAGTCCCT**CAAGTCCTTCC**A--------------------------AGCAGCAGCAGCAGCAGCAGCAGCAGCAGCAGCAGCAGCAGCAGCAGCAGCAGCAGCAGCAGCAGCAGCAGCAGCAGCAGCAGCAGCAGCAGCAG**CAACAGCCG**CCACCGCCGCCGCCGCCGCCGCCTCCTCAGCTTCCTCAGCCGCCGCGCAGGCACAGCCGCTGCTGCCTCAGC 242 421 DEL
1115 0.0
GCTGATGAAGGCCTTCGAGTCCCT**CAAGTCCTTCC**AGCAGCAGCAGCAGCAGCAGCAGCAGCAGCAGCAGCAGCAGCAGCAGCAGCAGCAGCAGCAGCAGCAGCAGCAGCAGCAGCAGCAGCAGCAGCAGCAGCAGCAGCAGCAGCAGCAGCAGCAGCAG**CAACAGCCG**CCACCGCCGCCGCCGCCGCCGCCTCCTCAGCTTCCTCAGCCGCCGCGCAGGCACAGCCGCTGCTGCCTCAGC
GCTGATGAAGGCCTTCGAGTCCCTCAAGT--CACCACCTCAAGCCGCCACCGCCGCCGCCGCCGCCGCCTCCTCAGCTTCCTCAGCCGCCGCGCAGGCACAGCCGCTGCTGCCTCAGC 242 418 DEL
337 34.0
GCTGATGAAGGCCTTCGAGTCCCT**CAAGTCCTTCC**AGCAGCAGCAGCAGCAGCAGCAGCAGCAGCAGCAGCAGCAGCAGCAGCAGCAGCAGCAGCAGCAGCAGCAGCAGCAGCAGCAGCAGCAGCAGCAGCAGCAGCAGCAGCAGCAGCAGCAGCAGCAG**CAACAGCCG**CCACCGCCGCCGCCGCCGCCGCCTCCTCAGCTTCCTCAGCCGCCGCGCAGGCACAGCCGCTGCTGCCTCAGC
GCTGATGAAGGCCTTCGAGTCCCT**CAAGTCCTTCC**A-----------------AGCAGCAGCAGCAGCAGCAGCAGCAGCAGCAGCAGCAGCAGCAGCAGCAGCAGCAGCAGCAGCAGCAGCAGCAGCAGCAGCAGCAGCAGCAGCAGCAGCAGCAG**CAACAGCCG**CCACCGCCGCCGCCGCCGCCGCCTCCTCAGCTTCCTCAGCCGCCGCGCAGGCACAGCCGCTGCTGCCTCAGC 242 412 DEL
464 29.0
GCTGATGAAGGCCTTCGAGTCCCT**CAAGTCCTTCC**AGCAGCAGCAGCAGCAGCAGCAGCAGCAGCAGCAGCAGCAGCAGCAGCAGCAGCAGCAGCAGCAGCAGCAGCAGCAGCAGCAGCAGCAGCAGCAGCAGCAGCAGCAGCAGCAGCAGCAGCAGCAG**CAACAGCCG**CCACCGCCGCCGCCGCCGCCGCCTCCTCAGCTTCCTCAGCCGCCGCGCAGGCACAGCCGCTGCTGCCTCAGC
GCTGATGAAGGCCTTCGAGTCCCT**CAAGTCCTTCC**A--------------------------------AGCAGCAGCAGCAGCAGCAGCAGCAGCAGCAGCAGCAGCAGCAGCAGCAGCAGCAGCAGCAGCAGCAGCAGCAGCAGCAGCAGCAGCAG**CAACAGCCG**CCACCGCCGCCGCCGCCGCCGCCTCCTCAGCTTCCTCAGCCGCCGCGCAGGCACAGCCGCTGCTGCCTCAGC 242 404 DEL
1221 0.0
GCTGATGAAGGCCTTCGAGTCCCT**CAAGTCCTTCC**AGCAGCAGCAGCAGCAGCAGCAGCAGCAGCAGCAGCAGCAGCAGCAGCAGCAGCAGCAGCAGCAGCAGCAGCAGCAGCAGCAGCAGCAGCAGCAGCAGCAGCAGCAGCAGCAGCAGCAGCAGCAG**CAACAGCCG**CCACCGCCGCCGCCGCCGCCGCCTCCTCAGCTTCCTCAGCCGCCGCGCAGGCACAGCCGCTGCTGCCTCAGC
GCTGATGAAGGCCTTCGAGT--CAG**CAACAGCCG**CCACCGCCGCCGCCGCCGCCGCCTCCTCAGCTTCCTCAGCCGCCGCGCAGGCACAGCCGCTGCTGCCTCAGC 242 393 DEL
1346 0.0
GCTGATGAAGGCCTTCGAGTCCCT**CAAGTCCTTCC**AGCAGCAGCAGCAGCAGCAGCAGCAGCAGCAGCAGCAGCAGCAGCAGCAGCAGCAGCAGCAGCAGCAGCAGCAGCAGCAGCAGCAGCAGCAGCAGCAGCAGCAGCAGCAGCAGCAGCAGCAGCAG**CAACAGCCG**CCACCGCCGCCGCCGCCGCCGCCTCCTCAGCTTCCTCAGCCGCCGCGCAGGCACAGCCGCTGCTGCCTCAGC
GCTGATGAAGGCCTTCGAGTCCCTCAA---GCCGCCGCCGCCGCCTCCTCAGCTTCCTCAGCCGCCGCGCAGGCACAGCCGCTGCTGCCTCAGC 242 370 DEL
536 26.0
GCTGATGAAGGCCTTCGAGTCCCT**CAAGTCCTTCC**AGCAGCAGCAGCAGCAGCAGCAGCAGCAGCAGCAGCAGCAGCAGCAGCAGCAGCAGCAGCAGCAGCAGCAGCAGCAGCAGCAGCAGCAGCAGCAGCAGCAGCAGCAGCAGCAGCAGCAGCAGCAG**CAACAGCCG**CCACCGCCGCCGCCGCCGCCGCCTCCTCAGCTTCCTCAGCCGCCGCGCAGGCACAGCCGCTGCTGCCTCAGC
GCTGATGAAGGCCTTCGAGTCCCT**CAAGTCCTTCC**A---AGCAGCAGCAGCAGCAGCAGCAGCAGCAGCAGCAGCAGCAGCAGCAGCAGCAGCAGCAGCAGCAGCAGCAGCAGCAGCAGCAG**CAACAGCCG**CCACCGCCGCCGCCGCCGCCGCCTCCTCAGCTTCCTCAGCCGCCGCGCAGGCACAGCCGCTGCTGCCTCAGC 242 368 DEL
362 33.0
GCTGATGAAGGCCTTCGAGTCCCT**CAAGTCCTTCC**AGCAGCAGCAGCAGCAGCAGCAGCAGCAGCAGCAGCAGCAGCAGCAGCAGCAGCAGCAGCAGCAGCAGCAGCAGCAGCAGCAGCAGCAGCAGCAGCAGCAGCAGCAGCAGCAGCAGCAGCAGCAG**CAACAGCCG**CCACCGCCGCCGCCGCCGCCGCCTCCTCAGCTTCCTCAGCCGCCGCGCAGGCACAGCCGCTGCTGCCTCAGC
GCTGATGAAGGCCTTCGAGTCCCT**CAAGTCCTTCC**A--------------------AGCAGCAGCAGCAGCAGCAGCAGCAGCAGCAGCAGCAGCAGCAGCAGCAGCAGCAGCAGCAGCAGCAGCAGCAGCAGCAGCAGCAGCAGCAGCAGCAGCAG**CAACAGCCG**CCACCGCCGCCGCCGCCGCCGCCTCCTCAGCTTCCTCAGCCGCCGCGCAGGCACAGCCGCTGCTGCCTCAGC 242 366 DEL
286 29.0
GCTGATGAAGGCCTTCGAGTCCCT**CAAGTCCTTCC**AGCAGCAGCAGCAGCAGCAGCAGCAGCAGCAGCAGCAGCAGCAGCAGCAGCAGCAGCAGCAGCAGCAGCAGCAGCAGCAGCAGCAGCAGCAGCAGCAGCAGCAGCAGCAGCAGCAGCAGCAGCAG**CAACAGCCG**CCACCGCCGCCGCCGCCGCCGCCTCCTCAGCTTCCTCAGCCGCCGCGCAGGCACAGCCGCTGCTGCCTCAGC
GCTGATGAAGGCCTTCGAGTC------------CCAGCAGCAGCAGCAGCAGCAGCAGCAGCAGCAGTAGCAGCAGCAGCAGCAGCAGCAGCAGCAGCAGCAGCAGCAGCAGCAGCAGCAGCAGCAGCAGCAGCAGCAGCAGCAGCAGCAGCAGCAG**CAACAGCCG**CCACCGCCGCCGCCGCCGCCGCCTCCTCAGCTTCCTCAGCCGCCGCGCAGGCACAGCCGCTGCTGCCTCAGC 242 362 DEL
1257 0.0
GCTGATGAAGGCCTTCGAGTCCCT**CAAGTCCTTCC**AGCAGCAGCAGCAGCAGCAGCAGCAGCAGCAGCAGCAGCAGCAGCAGCAGCAGCAGCAGCAGCAGCAGCAGCAGCAGCAGCAGCAGCAGCAGCAGCAGCAGCAGCAGCAGCAGCAGCAGCAGCAG**CAACAGCCG**CCACCGCCGCCGCCGCCGCCGCCTCCTCAGCTTCCTCAGCCGCCGCGCAGGCACAGCCGCTGCTGCCTCAGC
GCTGATGAAGGCCTTCGAGTCCCTCAAGTCCTTCAGTC--CCGCCGCCGCCGCCGCCTCCTCAGCTTCCTCAGCCGCCGCGCAGGCACAGCCGCTGCTGCCTCAGC 242 357 DEL
956 7.0
GCTGATGAAGGCCTTCGAGTCCCT**CAAGTCCTTCC**AGCAGCAGCAGCAGCAGCAGCAGCAGCAGCAGCAGCAGCAGCAGCAGCAGCAGCAGCAGCAGCAGCAGCAGCAGCAGCAGCAGCAGCAGCAGCAGCAGCAGCAGCAGCAGCAGCAGCAGCAGCAG**CAACAGCCG**CCACCGCCGCCGCCGCCGCCGCCTCCTCAGCTTCCTCAGCCGCCGCGCAGGCACAGCCGCTGCTGCCTCAGC
GCTGATGAAGGCCTTCGAGTCCCT**CAAGTCCTTCC**A--AGCAGCAGCAGCAGCAGCAGCAG**CAACAGCCG**CCACCGCCGCCGCCGCCGCCGCCTCCTCAGCTTCCTCAGCCGCCGCGCAGGCACAGCCGCTGCTGCCTCAGC 242 354 DEL
918 9.0
GCTGATGAAGGCCTTCGAGTCCCT**CAAGTCCTTCC**AGCAGCAGCAGCAGCAGCAGCAGCAGCAGCAGCAGCAGCAGCAGCAGCAGCAGCAGCAGCAGCAGCAGCAGCAGCAGCAGCAGCAGCAGCAGCAGCAGCAGCAGCAGCAGCAGCAGCAGCAGCAG**CAACAGCCG**CCACCGCCGCCGCCGCCGCCGCCTCCTCAGCTTCCTCAGCCGCCGCGCAGGCACAGCCGCTGCTGCCTCAGC
GCTGATGAAGGCCTTCGAGTCCCT**CAAGTCCTTCC**A--AGCAGCAGCAGCAGCAGCAGCAGCAGCAG**CAACAGCCG**CCACCGCCGCCGCCGCCGCCGCCTCCTCAGCTTCCTCAGCCGCCGCGCAGGCACAGCCGCTGCTGCCTCAGC 242 354 DEL
658 21.0
GCTGATGAAGGCCTTCGAGTCCCT**CAAGTCCTTCC**AGCAGCAGCAGCAGCAGCAGCAGCAGCAGCAGCAGCAGCAGCAGCAGCAGCAGCAGCAGCAGCAGCAGCAGCAGCAGCAGCAGCAGCAGCAGCAGCAGCAGCAGCAGCAGCAGCAGCAGCAGCAG**CAACAGCCG**CCACCGCCGCCGCCGCCGCCGCCTCCTCAGCTTCCTCAGCCGCCGCGCAGGCACAGCCGCTGCTGCCTCAGC
GCTGATGAAGGCCTTCGAGTCCCT**CAAGTCCTTCC**A--AGCAGCAGCAGCAGCAGCAGCAGCAGCAGCAGCAGCAGCAGCAGCAGCAGCAGCAGCAGCAGCAG**CAACAGCCG**CCACCGCCGCCGCCGCCGCCGCCTCCTCAGCTTCCTCAGCCGCCGCGCAGGCACAGCCGCTGCTGCCTCAGC 242 351 DEL
465 40.0
GCTGATGAAGGCCTTCGAGTCCCT**CAAGTCCTTCC**-AGCAGCAGCAGCAGCAGCAGCAGCAGCAGCAGCAGCAGCAGCAGCAGCAGCAGCAGCAGCAGCAGCAGCAGCAGCAGCAGCAGCAGCAGCAGCAGCAGCAGCAGCAGCAGCAGCAGCAGCAGCAG**CAACAGCCG**CCACCGCCGCCGCCGCCGCCGCCTCCTCAGCTTCCTCAGC
GCTGATGAAGGCCTTCGAGTCCCT**CAAGTCCTTCC**AAGCAGCAGCAGCAGCAGCAGCAGCAGCAGCAGCAGCAGCAGCAGCAGCAGCAGCAGCAGCAGCAGCAGCAGCAGCAGCAGCAGCAGCAGCAGCAGCAGCAGCAGCAGCAGCAGCAGCAGCAGCAG**CAACAGCCG**CCACCGCCGCCGCCGCCGCCGCCTCCTCAGCTTCCTCAGC 209 344 INS
787 12.0
GCTGATGAAGGCCTTCGAGTCCCT**CAAGTCCTTCC**AGCAGCAGCAGCAGCAGCAGCAGCAGCAGCAGCAGCAGCAGCAGCAGCAGCAGCAGCAGCAGCAGCAGCAGCAGCAGCAGCAGCAGCAGCAGCAGCAGCAGCAGCAGCAGCAGCAGCAGCAGCAG**CAACAGCCG**CCACCGCCGCCGCCGCCGCCGCCTCCTCAGCTTCCTCAGCCGCCGCGCAGGCACAGCCGCTGCTGCCTCAGC
GCTGATGAAGGCCTTCGAGTCCCT**CAAGTCCTTCC**A---CAGCAGCTGCTGCAGCAGCAGCAGCAGCAGCAGCAGCAGCAGCAGCAG**CAACAGCCG**CCACCGCCGCCGCCGCCGCCGCCTCCTCAGCTTCCTCAGCCGCCGCGCAGGCACAGCCGCTGCTGCCTCAGC 242 343 DEL
349 33.0
GCTGATGAAGGCCTTCGAGTCCCT**CAAGTCCTTCC**AGCAGCAGCAGCAGCAGCAGCAGCAGCAGCAGCAGCAGCAGCAGCAGCAGCAGCAGCAGCAGCAGCAGCAGCAGCAGCAGCAGCAGCAGCAGCAGCAGCAGCAGCAGCAGCAGCAGCAGCAGCAG**CAACAGCCG**CCACCGCCGCCGCCGCCGCCGCCTCCTCAGCTTCCTCAGCCGCCGCGCAGGCACAGCCGCTGCTGCCTCAGC
GCTGATGAAGGCCTTCGAGTCCCT**CAAGTCCTTCC**AGC------------------TGCAGCAGCAGCAGCAGCAGCAGCAGCAGCAGCAGCAGCAGCAGCAGCAGCAGCAGCAGCAGCAGCAGCAGCAGCAGCAGCAGCAGCAGCAGCAGCAGCAG**CAACAGCCG**CCACCGCCGCCGCCGCCGCCGCCTCCTCAGCTTCCTCAGCCGCCGCGCAGGCACAGCCGCTGCTGCCTCAGC 242 343 DEL
1308 0.0
GCTGATGAAGGCCTTCGAGTCCCT**CAAGTCCTTCC**AGCAGCAGCAGCAGCAGCAGCAGCAGCAGCAGCAGCAGCAGCAGCAGCAGCAGCAGCAGCAGCAGCAGCAGCAGCAGCAGCAGCAGCAGCAGCAGCAGCAGCAGCAGCAGCAGCAGCAGCAGCAG**CAACAGCCG**CCACCGCCGCCGCCGCCGCCGCCTCCTCAGCTTCCTCAGCCGCCGCGCAGGCACAGCCGCTGCTGCCTCAGC
GCTGATGAAGGCCTTCGAGTCCCT---CACCGCCGCCGCCGCCGCCGCCTCCTCAGCTTCCTCAGCCGCCGCGCAGGCACAGCCGCTGCTGCCTCAGC 242 337 DEL
938 8.0
GCTGATGAAGGCCTTCGAGTCCCT**CAAGTCCTTCC**AGCAGCAGCAGCAGCAGCAGCAGCAGCAGCAGCAGCAGCAGCAGCAGCAGCAGCAGCAGCAGCAGCAGCAGCAGCAGCAGCAGCAGCAGCAGCAGCAGCAGCAGCAGCAGCAGCAGCAGCAGCAG**CAACAGCCG**CCACCGCCGCCGCCGCCGCCGCCTCCTCAGCTTCCTCAGCCGCCGCGCAGGCACAGCCGCTGCTGCCTCAGC
GCTGATGAAGGCCTTCGAGTCCCT**CAAGTCCTTCC**A---AGCAGCAGCAGCAGCAGCAGCAGCAG**CAACAGCCG**CCACCGCCGCCGCCGCCGCCGCCTCCTCAGCTTCCTCAGCCGCCGCGCAGGCACAGCCGCTGCTGCCTCAGC 242 313 DEL
490 28.0
GCTGATGAAGGCCTTCGAGTCCCT**CAAGTCCTTCC**AGCAGCAGCAGCAGCAGCAGCAGCAGCAGCAGCAGCAGCAGCAGCAGCAGCAGCAGCAGCAGCAGCAGCAGCAGCAGCAGCAGCAGCAGCAGCAGCAGCAGCAGCAGCAGCAGCAGCAGCAGCAG**CAACAGCCG**CCACCGCCGCCGCCGCCGCCGCCTCCTCAGCTTCCTCAGCCGCCGCGCAGGCACAGCCGCTGCTGCCTCAGC
GCTGATGAAGGCCTTCGAGTCCCT**CAAGTCCTTCC**A-----------------------------------AGCAGCAGCAGCAGCAGCAGCAGCAGCAGCAGCAGCAGCAGCAGCAGCAGCAGCAGCAGCAGCAGCAGCAGCAGCAGCAGCAGCAGCAGCAG**CAACAGCCG**CCACCGCCGCCGCCGCCGCCGCCTCCTCAGCTTCCTCAGCCGCCGCGCAGGCACAGCCGCTGCTGCCTCAGC 242 311 DEL
391 32.0
GCTGATGAAGGCCTTCGAGTCCCT**CAAGTCCTTCC**AGCAGCAGCAGCAGCAGCAGCAGCAGCAGCAGCAGCAGCAGCAGCAGCAGCAGCAGCAGCAGCAGCAGCAGCAGCAGCAGCAGCAGCAGCAGCAGCAGCAGCAGCAGCAGCAGCAGCAGCAGCAG**CAACAGCCG**CCACCGCCGCCGCCGCCGCCGCCTCCTCAGCTTCCTCAGCCGCCGCGCAGGCACAGCCGCTGCTGCCTCAGC
GCTGATGAAGGCCTTCGAGTCCCT**CAAGTCCTTCC**A-----------------------AGCAGCAGCAGCAGCAGCAGCAGCAGCAGCAGCAGCAGCAGCAGCAGCAGCAGCAGCAGCAGCAGCAGCAGCAGCAGCAGCAGCAGCAGCAGCAGCAGCAG**CAACAGCCG**CCACCGCCGCCGCCGCCGCCGCCTCCTCAGCTTCCTCAGCCGCCGCGCAGGCACAGCCGCTGCTGCCTCAGC 242 311 DEL
1127 0.0
GCTGATGAAGGCCTTCGAGTCCCT**CAAGTCCTTCC**AGCAGCAGCAGCAGCAGCAGCAGCAGCAGCAGCAGCAGCAGCAGCAGCAGCAGCAGCAGCAGCAGCAGCAGCAGCAGCAGCAGCAGCAGCAGCAGCAGCAGCAGCAGCAGCAGCAGCAGCAGCAG**CAACAGCCG**CCACCGCCGCCGCCGCCGCCGCCTCCTCAGCTTCCTCAGCCGCCGCGCAGGCACAGCCGCTGCTGCCTCAGC
GCTGATGAAGGCCTTCGAGTCCCTCAAGTCCTTC---**CAACAGCCG**CCACCGCCGCCGCCGCCGCCGCCTCCTCAGCTTCCTCAGCCGCCGCGCAGGCACAGCCGCTGCTGCCTCAGC 242 309 DEL
537 40.0
GCTGATGAAGGCCTTCGAGTCCCT**CAAGTCCTTCC**-AGCAGCAGCAGCAGCAGCAGCAGCAGCAGCAGCAGCAGCAGCAGCAGCAGCAGCAGCAGCAGCAGCAGCAGCAGCAGCAGCAGCAGCAGCAGCAGCAGCAGCAGCAGCAGCAGCAGCAGCAGCAG**CAACAGCCG**CCACCGCCGCCGCCGCCGCCGCCTCCTCAGC
GCTGATGAAGGCCTTCGAGTCCCT**CAAGTCCTTCC**AAGCAGCAGCAGCAGCAGCAGCAGCAGCAGCAGCAGCAGCAGCAGCAGCAGCAGCAGCAGCAGCAGCAGCAGCAGCAGCAGCAGCAGCAGCAGCAGCAGCAGCAGCAGCAGCAGCAGCAGCAGCAG**CAACAGCCG**CCACCGCCGCCGCCGCCGCCGCCTCCTCAGC 200 304 INS
1493 0.0
GCTGATGAAGGCCTTCGAGTCCCT**CAAGTCCTTCC**AGCAGCAGCAGCAGCAGCAGCAGCAGCAGCAGCAGCAGCAGCAGCAGCAGCAGCAGCAGCAGCAGCAGCAGCAGCAGCAGCAGCAGCAGCAGCAGCAGCAGCAGCAGCAGCAGCAGCAGCAGCAG**CAACAGCCG**CCACCGCCGCCGCCGCCGCCGCCTCCTCAGCTTCCTCAGCCGCCGCGCAGGCACAGCCGCTGCTGCCTCAGC

GCTGATGAAGGCCTTCGAGTCCCT**CAAGTCCTTCC**--GCCGCCGCCGCAGGCACAGCCGCTGCTGCCTCAGC 242 303 DEL
66 43.0
GCTGATGAAGGCCTTCGAGTCCCTCAAGTCCTTC----------CAGCAGCAGCAGCAGCAGCAGCAGCAGCAGCAGCAGCAGCAGCAGCAGCAGCAGCAGCAGCAGCAGCAGCAGCAGCAGCAGCAGCAGCAGCAGCAGCAGCAGCAGCAGCAGCAGCAGCAGCAGCAG**CAACAGCCG**CCACCGCCGCCGCCGCCGCCGCCGCCTCCTCAGCTTCCTCAGCCGCCGCCGCAGGCACAGCCGCTGCTGCCTCAGC
GCTGATGAAGGCCTTCGAGTCCCT**CAAGTCCTTCC**AAGCAGCAGCAGCAGCAGCAGCAGCAGCAGCAGCAGCAGCAGCAGCAGCAGCAGCAGCAGCAGCAGCAGCAGCAGCAGCAGCAGCAGCAGCAGCAGCAGCAGCAGCAGCAGCAGCAGCAGCAGCAGCAGCAGCAG**CAACAGCCG**CCACCGCCGCCGCCGCCGCCGCCGCCTCCTCAGCTTCCTCAGCCGCCGCCGCAGGCACAGCCGCTGCTGCCTCAGC 242 294 INS

1434 0.0
GCTGATGAAGGCCTTCGAGTCCCT**CAAGTCCTTCC**AGCAGCAGCAGCAGCAGCAGCAGCAGCAGCAGCAGCAGCAGCAGCAGCAGCAGCAGCAGCAGCAGCAGCAGCAGCAGCAGCAGCAGCAGCAGCAGCAGCAGCAGCAGCAGCAGCAGCAGCAGCAG**CAACAGCCG**CCACCGCCGCCGCCGCCGCCGCCGCCTCCTCAGCTTCCTCAGCCGCCGCCGCAGGCACAGCCGCTGCTGCCTCAGC
GCTGATGAAGGCCTTCGAGTCCCTCAAGT---CGCCTCCTCAGCTTCCTCAGCCGCCGCCGCAGGCACAGCCGCTGCTGCCTCAGC 242 291 DEL
1427 0.0
GCTGATGAAGGCCTTCGAGTCCCT**CAAGTCCTTCC**AGCAGCAGCAGCAGCAGCAGCAGCAGCAGCAGCAGCAGCAGCAGCAGCAGCAGCAGCAGCAGCAGCAGCAGCAGCAGCAGCAGCAGCAGCAGCAGCAGCAGCAGCAGCAGCAGCAGCAGCAGCAG**CAACAGCCG**CCACCGCCGCCGCCGCCGCCGCCGCCTCCTCAGCTTCCTCAGCCGCCGCCGCAGGCACAGCCGCTGCTGCCTCAGC
GCTGATGAAGGCCTTCGAGTCCCTCAAGT--CCGCCTCCTCAGCTTCCTCAGCCGCCGCCGCAGGCACAGCCGCTGCTGCCTCAGC 242 289 DEL
1163 5.0
GCTGATGAAGGCCTTCGAGTCCCT**CAAGTCCTTCC**AGCAGCAGCAGCAGCAGCAGCAGCAGCAGCAGCAGCAGCAGCAGCAGCAGCAGCAGCAGCAGCAGCAGCAGCAGCAGCAGCAGCAGCAGCAGCAGCAGCAGCAGCAGCAGCAGCAGCAGCAGCAG**CAACAGCCG**CCACCGCCGCCGCCGCCGCCGCCGCCTCCTCAGCTTCCTCAGCCGCCGCCGCAGGCACAGCCGCTGCTGCCTCAGC
GCTGATGAAGGCCTTCGAGTCCCT**CAAGTCCTTCC**A---CAGCAGCAGCA-----------------GCCGCCGCCGCCGCCTCCTCAGCTTCCTCAGCCGCCGCCGCAGGCACAGCCGCTGCTGCCTCAGC 242 287 DEL
644 25.0
GCTGATGAAGGCCTTCGAGTCCCT**CAAGTCCTTCC**AGCAGCAGCAGCAGCAGCAGCAGCAGCAGCAGCAGCAGCAGCAGCAGCAGCAGCAGCAGCAGCAGCAGCAGCAGCAGCAGCAGCAGCAGCAGCAGCAGCAGCAGCAGCAGCAGCAGCAGCAGCAG**CAACAGCCG**CCACCGCCGCCGCCGCCGCCGCCGCCTCCTCAGCTTCCTCAGCCGCCGCCGCAGGCACAGCCGCTGCTGCCTCAGC
GCTGATGAAGGCCTTCGAGTCCCTCA--AGCAGCAGCAGCAGCAGCAGCAGCAGCAGCAGCAGCAGCAGCAGCAGCAGCAGCAGCAGCAGCAGCAGCAGCAGCAGCAG**CAACAGCCG**CCACCGCCGCCGCCGCCGCCGCCGCCTCCTCAGCTTCCTCAGCCGCCGCCGCAGGCACAGCCGCTGCTGCCTCAGC 242 286 DEL
749 17.0
GCTGATGAAGGCCTTCGAGTCCCT**CAAGTCCTTCC**AGCAGCAGCAGCAGCAGCAGCAGCAGCAGCAGCAGCAGCAGCAGCAGCAGCAGCAGCAGCAGCAGCAGCAGCAGCAGCAGCAGCAGCAGCAGCAGCAGCAGCAGCAGCAGCAGCAGCAGCAGCAG**CAACAGCCG**CCACCGCCGCCGCCGCCGCCGCCGCCTCCTCAGCTTCCTCAGCCGCCGCCGCAGGCACAGCCGCTGCTGCCTCAGC
GCTGATGAAGGCCTTCGAGTCCCT**CAAGTCCTTCC**A--AGCAGCAGCAGCAGCAGCAGCAGCAGCAGCAGCAGCAGCAGCAGCAGCAGCAGCAG**CAACAGCCG**CCACCGCCGCCGCCGCCGCCGCCGCCTCCTCAGCTTCCTCAGCCGCCGCCGCAGGCACAGCCGCTGCTGCCTCAGC 242 281 DEL
945 11.0
GCTGATGAAGGCCTTCGAGTCCCT**CAAGTCCTTCC**AGCAGCAGCAGCAGCAGCAGCAGCAGCAGCAGCAGCAGCAGCAGCAGCAGCAGCAGCAGCAGCAGCAGCAGCAGCAGCAGCAGCAGCAGCAGCAGCAGCAGCAGCAGCAGCAGCAGCAGCAGCAG**CAACAGCCG**CCACCGCCGCCGCCGCCGCCGCCGCCTCCTCAGCTTCCTCAGCCGCCGCCGCAGGCACAGCCGCTGCTGCCTCAGC
GCTGATGAAGGCCTTCGAGTCCCTCA--AGCAGCAGCAGCAGCAGCAGCAGCAGCAGCAGCAG**CAACAGCCG**CCACCGCCGCCGCCGCCGCCGCCGCCTCCTCAGCTTCCTCAGCCGCCGCCGCAGGCACAGCCGCTGCTGCCTCAGC 242 278 DEL
491 39.0
GCTGATGAAGGCCTTCGAGTCCCT**CAAGTCCTTCC**AGCAGCAGCAGCAGCAGCAGCAGCAGCAGCAGCAGCAGCAGCAGCAGCAGCAGCAGCAGCAGCAGCAGCAGCAGCAGCAGCAGCAGCAGCAGCAGCAGCAGCAGCAGCAGCAGCAGCAGCAGCAG**CAACAGCCG**CCACCGCCGCCGCCGCCGCCGCCGCCTCCTCAGCTTCCTCAGC
GCTGATGAAGGCCTTCGAGTCCCT**CAAGTCCTTCC**A--AGCAGCAGCAGCAGCAGCAGCAGCAGCAGCAGCAGCAGCAGCAGCAGCAGCAGCAGCAGCAGCAGCAGCAGCAGCAGCAGCAGCAGCAGCAGCAGCAGCAGCAGCAGCAGCAGCAGCAG**CAACAGCCG**CCACCGCCGCCGCCGCCGCCGCCGCCTCCTCAGCAGCCTCAGC 209 275 DEL
1068 3.0
GCTGATGAAGGCCTTCGAGTCCCT**CAAGTCCTTCC**AGCAGCAGCAGCAGCAGCAGCAGCAGCAGCAGCAGCAGCAGCAGCAGCAGCAGCAGCAGCAGCAGCAGCAGCAGCAGCAGCAGCAGCAGCAGCAGCAGCAGCAGCAGCAGCAGCAGCAGCAGCAG**CAACAGCCG**CCACCGCCGCCGCCGCCGCCGCCGCCTCCTCAGCTTCCTCAGCCGCCGCCGCAGGCACAGCCGCTGCTGCCTCAGC
GCTGATGAAGGCCTTCGAGTCCCTCAAGTCCTTC--CAGCAGCAG**CAACAGCCG**CCACCGCCGCCGCCGCCGCCGCCGCCTCCTCAGCTTCCTCAGCCGCCGCCGCAGGCACAGCCGCTGCTGCCTCAGC 242 264 DEL
1509 0.0
GCTGATGAAGGCCTTCGAGTCCCT**CAAGTCCTTCC**AGCAGCAGCAGCAGCAGCAGCAGCAGCAGCAGCAGCAGCAGCAGCAGCAGCAGCAGCAGCAGCAGCAGCAGCAGCAGCAGCAGCAGCAGCAGCAGCAGCAGCAGCAGCAGCAGCAGCAGCAGCAG**CAACAGCCG**CCACCGCCGCCGCCGCCGCCGCCGCCTCCTCAGCTTCCTCAGCCGCCGCCGCAGGCACAGCCGCTGCTGCCTCAGC
GCTGATGAAGGCCTTCGAGTCCCTCAAGTCCTT---CCGCCGCCGCAGGCACAGCCGCTGCTGCCTCAGC 242 256 DEL
510 27.0
GCTGATGAAGGCCTTCGAGTCCCT**CAAGTCCTTCC**AGCAGCAGCAGCAGCAGCAGCAGCAGCAGCAGCAGCAGCAGCAGCAGCAGCAGCAGCAGCAGCAGCAGCAGCAGCAGCAGCAGCAGCAGCAGCAGCAGCAGCAGCAGCAGCAGCAGCAGCAGCAG**CAACAGCCG**CCACCGCCGCCGCCGCCGCCGCCGCCTCCTCAGCTTCCTCAGCCGCCGCCGCAGGCACAGCCGCTGCTGCCTCAGC
GCTGATGAAGGCCTTCGAGTCCCT**CAAGTCCTTCC**A--------------------------------------AGCAGCAGCAGCAGCAGCAGCAGCAGCAGCAGCAGCAGCAGCAGCAGCAGCAGCAGCAGCAGCAGCAGCAGCAGCAGCAGCAGCAGCAG**CAACAGCCG**CCACCGCCGCCGCCGCCGCCGCCGCCTCCTCAGCTTCCTCAGCCGCCGCCGCAGGCACAGCCGCTGCTGCCTCAGC 242 251 DEL
817 17.0
GCTGATGAAGGCCTTCGAGTCCCT**CAAGTCCTTCC**AGCAGCAGCAGCAGCAGCAGCAGCAGCAGCAGCAGCAGCAGCAGCAGCAGCAGCAGCAGCAGCAGCAGCAGCAGCAGCAGCAGCAGCAGCAGCAGCAGCAGCAGCAGCAGCAGCAGCAGCAGCAG**CAACAGCCG**CCACCGCCGCCGCCGCCGCCGCCGCCTCCTCAGCTTCCTCAGCCGCCGCCGCAGGCACAGCCGCTGCTGCCTCAGC
GCTGATGAAGGCCTTCGAGTCCCTCAAGT---CAGCAGCAGCAGCAGCAGCAGCAGCAGCAGCAGCAGCAGCAGCAGCAGCAG**CAACAGCCG**CCACCGCCGCCGCCGCCGCCGCCGCCTCCTCAGCTTCCTCAGCCGCCGCCGCAGGCACAGCCGCTGCTGCCTCAGC 242 249 DEL
1514 0.0
GCTGATGAAGGCCTTCGAGTCCCT**CAAGTCCTTCC**AGCAGCAGCAGCAGCAGCAGCAGCAGCAGCAGCAGCAGCAGCAGCAGCAGCAGCAGCAGCAGCAGCAGCAGCAGCAGCAGCAGCAGCAGCAGCAGCAGCAGCAGCAGCAGCAGCAGCAGCAGCAG**CAACAGCCG**CCACCGCCGCCGCCGCCGCCGCCGCCTCCTCAGCTTCCTCAGCCGCCGCCGCAGGCACAGCCGCTGCTGCCTCAGC
GCTGATGAAGGCCTTCGAGTCCCTCAAGTCC--GCCGCCGCCGCAGGCACAGCCGCTGCTGCCTCAGC 242 241 DEL
834 13.0
GCTGATGAAGGCCTTCGAGTCCCT**CAAGTCCTTCC**AGCAGCAGCAGCAGCAGCAGCAGCAGCAGCAGCAGCAGCAGCAGCAGCAGCAGCAGCAGCAGCAGCAGCAGCAGCAGCAGCAGCAGCAGCAGCAGCAGCAGCAGCAGCAGCAGCAGCAGCAGCAG**CAACAGCCG**CCACCGCCGCCGCCGCCGCCGCCGCCTCCTCAGCTTCCTCAGCCGCCGCCGCAGGCACAGCCGCTGCTGCCTCAGC
GCTGATGAAGGCCTTCGAGTCCCT**CAAGTCCTTCC**A--AGCAGCAGCAGCAGCAGCAGCAGCAGCAGCAGCAGCAGCAG**CAACAGCCG**CCACCGCCGCCGCCGCCGCCGCCGCCTCCTCAGCTTCCTCAGCCGCCGCCGCAGGCACAGCCGCTGCTGCCTCAGC 242 240 DEL
1132 0.0
GCTGATGAAGGCCTTCGAGTCCCT**CAAGTCCTTCC**AGCAGCAGCAGCAGCAGCAGCAGCAGCAGCAGCAGCAGCAGCAGCAGCAGCAGCAGCAGCAGCAGCAGCAGCAGCAGCAGCAGCAGCAGCAGCAGCAGCAGCAGCAGCAGCAGCAGCAGCAGCAG**CAACAGCCG**CCACCGCCGCCGCCGCCGCCGCCGCCTCCTCAGCTTCCTCAGCCGCCGCCGCAGGCACAGCCGCTGCTGCCTCAGC
GCTGATGAAGGCCTTCGAGTCCCT**CAAGTCCTTCC**--ACAGCCGCCACCGCCGCCGCCGCCGCCGCCGCCTCCTCAGCTTCCTCAGCCGCCGCCGCAGGCACAGCCGCTGCTGCCTCAGC 242 240 DEL
934 13.0
GCTGATGAAGGCCTTCGAGTCCCT**CAAGTCCTTCC**AGCAGCAGCAGCAGCAGCAGCAGCAGCAGCAGCAGCAGCAGCAGCAGCAGCAGCAGCAGCAGCAGCAGCAGCAGCAGCAGCAGCAGCAGCAGCAGCAGCAGCAGCAGCAGCAGCAGCAGCAGCAG**CAACAGCCG**CCACCGCCGCCGCCGCCGCCGCCGCCTCCTCAGCTTCCTCAGCCGCCGCCGCAGGCACAGCCGCTGCTGCCTCAGC
GCTGATGAAGGCCTTCGAGTCCCT--CAGCAGCAGCAGCAGCAGCAGCAGCAGCAGCAGCAGCAG**CAACAGCCG**CCACCGCCGCCGCCGCCGCCGCCGCCTCCTCAGCTTCCTCAGCCGCCGCCGCAGGCACAGCCGCTGCTGCCTCAGC 242 232 DEL
561 25.0
GCTGATGAAGGCCTTCGAGTCCCT**CAAGTCCTTCC**AGCAGCAGCAGCAGCAGCAGCAGCAGCAGCAGCAGCAGCAGCAGCAGCAGCAGCAGCAGCAGCAGCAGCAGCAGCAGCAGCAGCAGCAGCAGCAGCAGCAGCAGCAGCAGCAGCAGCAGCAGCAG**CAACAGCCG**CCACCGCCGCCGCCGCCGCCGCCGCCTCCTCAGCTTCCTCAGCCGCCGCCGCAGGCACAGCCGCTGCTGCCTCAGC
GCTGATGAAGGCCTTCGAGTCCCT**CAAGTCCTTCC**A--AGCAGCAGCAGCAGCAGCAGCAGCAGCAGCAGCAGCAGCAGCAGCAGCAGCAGCAGCAGCAGCAGCAGCAGCAGCAGCAG**CAACAGCCG**CCACCGCCGCCGCCGCCGCCGCCGCCTCCTCAGCTTCCTCAGCCGCCGCCGCAGGCACAGCCGCTGCTGCCTCAGC 242 219 DEL
503 32.0
GCTGATGAAGGCCTTCGAGTCCCT**CAAGTCCTTCC**AGCAGCAGCAGCAGCAGCAGCAGCAGCAGCAGCAGCAGCAGCAGCAGCAGCAGCAGCAGCAGCAGCAGCAGCAGCAGCAGCAGCAGCAGCAGCAGCAGCAGCAGCAGCAGCAGCAGCAGCAGCAG**CAACAGCCG**CCACCGCCGCCGCCGCCGCCGCCGCCTCCTCAGCTTCCTCAGCCGCCGCCGCAGGCACAGCCGCTGCTGCCTCAGC
GCTGATGAAGGCCTTCGAGTCCCT-------------------------------------CAGCAGCAGCAGCAGCAGCAGCAGCAGCAGCAGCAGCAGCAGCAGCAGCAGCAGCAGCAGCAGCAGCAGCAGCAGCAGCAGCAGCAGCAGCAGCAG**CAACAGCCG**CCACCGCCGCCGCCGCCGCCGCCGCCTCCTCAGCTTCCTCAGCCGCCGCCGCAGGCACAGCCGCTGCTGCCTCAGC 242 215 DEL
708 19.0
GCTGATGAAGGCCTTCGAGTCCCT**CAAGTCCTTCC**AGCAGCAGCAGCAGCAGCAGCAGCAGCAGCAGCAGCAGCAGCAGCAGCAGCAGCAGCAGCAGCAGCAGCAGCAGCAGCAGCAGCAGCAGCAGCAGCAGCAGCAGCAGCAGCAGCAGCAGCAGCAG**CAACAGCCG**CCACCGCCGCCGCCGCCGCCGCCGCCTCCTCAGCTTCCTCAGCCGCCGCCGCAGGCACAGCCGCTGCTGCCTCAGC
GCTGATGAAGGCCTTCGAGTCCCT**CAAGTCCTTCC**A--AGCAGCAGCAGCAGCAGCAGCAGCAGCAGCAGCAGCAGCAGCAGCAGCAGCAGCAGCAGCAG**CAACAGCCG**CCACCGCCGCCGCCGCCGCCGCCGCCTCCTCAGCTTCCTCAGCCGCCGCCGCAGGCACAGCCGCTGCTGCCTCAGC 242 209 DEL
330 35.0
GCTGATGAAGGCCTTCGAGTCCCT**CAAGTCCTTCC**AGCAGCAGCAGCAGCAGCAGCAGCAGCAGCAGCAGCAGCAGCAGCAGCAGCAGCAGCAGCAGCAGCAGCAGCAGCAGCAGCAGCAGCAGCAGCAGCAGCAGCAGCAGCAGCAGCAGCAGCAGCAG**CAACAGCCG**CCACCGCCGCCGCCGCCGCCGCCGCCTCCTCAGCTTCCTCAGCCGCCGCCGCAGGCACAGCCGCTGCTGCCTCAGC
GCTGATGAAGGCCTTCGAGTCCCT**CAAGTCCTTCC**A----------------CAGCAGCAGCAGCAGCAGCAGCAGCAGCAGCAGCAGCAGCAGCAGCAGCAGCAGCAGCAGCAGCAGCAGCAGCAGCAGCAGCAGCAGCAGCAGCAGCAGCAGCAGCAG**CAACAGCCG**CCACCGCCGCCGCCGCCGCCGCCGCCTCCTCAGCTTCCTCAGCCGCCGCCGCAGGCACAGCCGCTGCTGCCTCAGC 242 209 DEL
1478 0.0
GCTGATGAAGGCCTTCGAGTCCCT**CAAGTCCTTCC**AGCAGCAGCAGCAGCAGCAGCAGCAGCAGCAGCAGCAGCAGCAGCAGCAGCAGCAGCAGCAGCAGCAGCAGCAGCAGCAGCAGCAGCAGCAGCAGCAGCAGCAGCAGCAGCAGCAGCAGCAGCAG**CAACAGCCG**CCACCGCCGCCGCCGCCGCCGCCGCCTCCTCAGCTTCCTCAGCCGCCGCCGCAGGCACAGCCGCTGCTGCCTCAGC
GCTGATGAAGGCCTTCGAGTC---CCTCCTCAGCTTCCTCAGCCGCCGCCGCAGGCACAGCCGCTGCTGCCTCAGC 242 207 DEL
384 32.0
GCTGATGAAGGCCTTCGAGTCCCT**CAAGTCCTTCC**AGCAGCAGCAGCAGCAGCAGCAGCAGCAGCAGCAGCAGCAGCAGCAGCAGCAGCAGCAGCAGCAGCAGCAGCAGCAGCAGCAGCAGCAGCAGCAGCAGCAGCAGCAGCAGCAGCAGCAGCAGCAG**CAACAGCCG**CCACCGCCGCCGCCGCCGCCGCCGCCTCCTCAGCTTCCTCAGCCGCCGCCGCAGGCACAGCCGCTGCTGCCTCAGC
GCTGATGAAGGCCTTCGAGTCCCT**CAAGTCCTTCC**AG----------------------AGCAGCAGCAGCAGCAGCAGCAGCAGCAGCAGCAGCAGCAGCAGCAGCAGCAGCAGCAGCAGCAGCAGCAGCAGCAGCAGCAGCAGCAGCAGCAGCAGCAG**CAACAGCCG**CCACCGCCGCCGCCGCCGCCGCCGCCTCCTCAGCTTCCTCAGCCGCCGCCGCAGGCACAGCCGCTGCTGCCTCAGC 242 200 DEL
1479 0.0
GCTGATGAAGGCCTTCGAGTCCCT**CAAGTCCTTCC**AGCAGCAGCAGCAGCAGCAGCAGCAGCAGCAGCAGCAGCAGCAGCAGCAGCAGCAGCAGCAGCAGCAGCAGCAGCAGCAGCAGCAGCAGCAGCAGCAGCAGCAGCAGCAGCAGCAGCAGCAGCAG**CAACAGCCG**CCACCGCCGCCGCCGCCGCCGCCGCCTCCTCAGCTTCCTCAGCCGCCGCCGCAGGCACAGCCGCTGCTGCCTCAGC
GCTGATGAAGGCCTTCGAGTCCCT**CAAGTCCTTCC**GCC---GCCGCCGCCGCAGGCACAGCCGCTGCTGCCTCAGC 242 195 DEL
683 20.0
GCTGATGAAGGCCTTCGAGTCCCT**CAAGTCCTTCC**AGCAGCAGCAGCAGCAGCAGCAGCAGCAGCAGCAGCAGCAGCAGCAGCAGCAGCAGCAGCAGCAGCAGCAGCAGCAGCAGCAGCAGCAGCAGCAGCAGCAGCAGCAGCAGCAGCAGCAGCAGCAG**CAACAGCCG**CCACCGCCGCCGCCGCCGCCGCCGCCTCCTCAGCTTCCTCAGCCGCCGCCGCAGGCACAGCCGCTGCTGCCTCAGC
GCTGATGAAGGCCTTCGAGTCCCT**CAAGTCCTTCC**A---AGCAGCAGCAGCAGCAGCAGCAGCAGCAGCAGCAGCAGCAGCAGCAGCAGCAGCAGCAGCAGCAG**CAACAGCCG**CCACCGCCGCCGCCGCCGCCGCCGCCTCCTCAGCTTCCTCAGCCGCCGCCGCAGGCACAGCCGCTGCTGCCTCAGC 242 194 DEL
913 12.0
GCTGATGAAGGCCTTCGAGTCCCT**CAAGTCCTTCC**AGCAGCAGCAGCAGCAGCAGCAGCAGCAGCAGCAGCAGCAGCAGCAGCAGCAGCAGCAGCAGCAGCAGCAGCAGCAGCAGCAGCAGCAGCAGCAGCAGCAGCAGCAGCAGCAGCAGCAGCAGCAG**CAACAGCCG**CCACCGCCGCCGCCGCCGCCGCCGCCTCCTCAGCTTCCTCAGCCGCCGCCGCAGGCACAGCCGCTGCTGCCTCAGC
GCTGATGAAGGCCTTCGAGTCCCTCAAGTC---CAGCAGCAGCAGCAGCAGCAGCAGCAGCAGCAGCAG**CAACAGCCG**CCACCGCCGCCGCCGCCGCCGCCGCCTCCTCAGCTTCCTCAGCCGCCGCCGCAGGCACAGCCGCTGCTGCCTCAGC 242 193 DEL
482 31.0
GCTGATGAAGGCCTTCGAGTCCCT**CAAGTCCTTCC**AGCAGCAGCAGCAGCAGCAGCAGCAGCAGCAGCAGCAGCAGCAGCAGCAGCAGCAGCAGCAGCAGCAGCAGCAGCAGCAGCAGCAGCAGCAGCAGCAGCAGCAGCAGCAGCAGCAGCAGCAGCAG**CAACAGCCG**CCACCGCCGCCGCCGCCGCCGCCGCCTCCTCAGCTTCCTCAGCCGCCGCCGCAGGCACAGCCGCTGCTGCCTCAGC
GCTGATGAAGGCCTTCGAGTCCCTCAAGTC----------------------------------CAGCAGCAGCAGCAGCAGCAGCAGCAGCAGCAGCAGCAGCAGCAGCAGCAGCAGCAGCAGCAGCAGCAGCAGCAGCAGCAGCAGCAGCAGCAG**CAACAGCCG**CCACCGCCGCCGCCGCCGCCGCCGCCTCCTCAGCTTCCTCAGCCGCCGCCGCAGGCACAGCCGCTGCTGCCTCAGC 242 192 DEL
374 32.0
GCTGATGAAGGCCTTCGAGTCCCT**CAAGTCCTTCC**AGCAGCAGCAGCAGCAGCAGCAGCAGCAGCAGCAGCAGCAGCAGCAGCAGCAGCAGCAGCAGCAGCAGCAGCAGCAGCAGCAGCAGCAGCAGCAGCAGCAGCAGCAGCAGCAGCAGCAGCAGCAG**CAACAGCCG**CCACCGCCGCCGCCGCCGCCGCCGCCTCCTCAGCTTCCTCAGCCGCCGCCGCAGGCACAGCCGCTGCTGCCTCAGC
GCTGATGAAGGCCTTCGAGTCCCT**CAAGTCCTTCC**AGC---------------------TGCAGCAGCAGCAGCAGCAGCAGCAGCAGCAGCAGCAGCAGCAGCAGCAGCAGCAGCAGCAGCAGCAGCAGCAGCAGCAGCAGCAGCAGCAGCAGCAG**CAACAGCCG**CCACCGCCGCCGCCGCCGCCGCCGCCTCCTCAGCTTCCTCAGCCGCCGCCGCAGGCACAGCCGCTGCTGCCTCAGC 242 189 DEL
584 24.0
GCTGATGAAGGCCTTCGAGTCCCT**CAAGTCCTTCC**AGCAGCAGCAGCAGCAGCAGCAGCAGCAGCAGCAGCAGCAGCAGCAGCAGCAGCAGCAGCAGCAGCAGCAGCAGCAGCAGCAGCAGCAGCAGCAGCAGCAGCAGCAGCAGCAGCAGCAGCAGCAG**CAACAGCCG**CCACCGCCGCCGCCGCCGCCGCCGCCTCCTCAGCTTCCTCAGCCGCCGCCGCAGGCACAGCCGCTGCTGCCTCAGC
GCTGATGAAGGCCTTCGAGTCCCT**CAAGTCCTTCC**A---AGCAGCAGCAGCAGCAGCAGCAGCAGCAGCAGCAGCAGCAGCAGCAGCAGCAGCAGCAGCAGCAGCAGCAGCAGCAG**CAACAGCCG**CCACCGCCGCCGCCGCCGCCGCCGCCTCCTCAGCTTCCTCAGCCGCCGCCGCAGGCACAGCCGCTGCTGCCTCAGC 242 181 DEL
353 38.0
GCTGATGAAGGCCTTCGAGTCCCT**CAAGTCCTTCC**AGCAGCAGCAGCAGCAGCAGCAGCAGCAGCAGCAGCAGCAGCAGCAGCAGCAGCAGCAGCAGCAGCAGCAGCAGCAGCAGCAGCAGCAGCAGCAGCAGCAGCAGCAGCAGCAGCAGCAGCAGCAG**CAACAGCCG**CCACCGCCGCCGCCGCCGCCGCCGCCTCCTCAGCTTCCTCAGCCGCCGCCGCAGGCACAGCCGCTGCTGCCTCAGC
GCTGATGAAGGCCTTCGAGTCCCT-------------------CAGCAGCAGCAGCAGCAGCAGCAGCAGCAGCAGCAGCAGCAGCAGCAGCAGCAGCAGCAGCAGCAGCAGCAGCAGCAGCAGCAGCAGCAGCAGCAGCAGCAGCAGCAGCAGCAGCAG**CAACAGCCG**CCACCGCCGCCGCCGCCGCCGCCGCCTCCTCAGCTTCCTCAGCCGCCGCCGCAGGCACAGCCGCTGCTGCCTCAGC 242 177 DEL
729 18.0
GCTGATGAAGGCCTTCGAGTCCCT**CAAGTCCTTCC**AGCAGCAGCAGCAGCAGCAGCAGCAGCAGCAGCAGCAGCAGCAGCAGCAGCAGCAGCAGCAGCAGCAGCAGCAGCAGCAGCAGCAGCAGCAGCAGCAGCAGCAGCAGCAGCAGCAGCAGCAGCAG**CAACAGCCG**CCACCGCCGCCGCCGCCGCCGCCGCCTCCTCAGCTTCCTCAGCCGCCGCCGCAGGCACAGCCGCTGCTGCCTCAGC
GCTGATGAAGGCCTTCGAGTCCCT**CAAGTCCTTCC**A---AGCAGCAGCAGCAGCAGCAGCAGCAGCAGCAGCAGCAGCAGCAGCAGCAGCAGCAGCAG**CAACAGCCG**CCACCGCCGCCGCCGCCGCCGCCGCCTCCTCAGCTTCCTCAGCCGCCGCCGCAGGCACAGCCGCTGCTGCCTCAGC 242 175 DEL
457 32.0
GCTGATGAAGGCCTTCGAGTCCCT**CAAGTCCTTCC**AGCAGCAGCAGCAGCAGCAGCAGCAGCAGCAGCAGCAGCAGCAGCAGCAGCAGCAGCAGCAGCAGCAGCAGCAGCAGCAGCAGCAGCAGCAGCAGCAGCAGCAGCAGCAGCAGCAGCAGCAGCAG**CAACAGCCG**CCACCGCCGCCGCCGCCGCCGCCGCCTCCTCAGCTTCCTCAGCCGCCGCCGCAGGCACAGCCGCTGCTGCCTCAGC
GCTGATGAAGGCCTTCGAGTCCCTCAAGTC-------------------------------CAGCAGCAGCAGCAGCAGCAGCAGCAGCAGCAGCAGCAGCAGCAGCAGCAGCAGCAGCAGCAGCAGCAGCAGCAGCAGCAGCAGCAGCAGCAGCAG**CAACAGCCG**CCACCGCCGCCGCCGCCGCCGCCGCCTCCTCAGCTTCCTCAGCCGCCGCCGCAGGCACAGCCGCTGCTGCCTCAGC 242 175 DEL
426 34.0
GCTGATGAAGGCCTTCGAGTCCCT**CAAGTCCTTCC**AGCAGCAGCAGCAGCAGCAGCAGCAGCAGCAGCAGCAGCAGCAGCAGCAGCAGCAGCAGCAGCAGCAGCAGCAGCAGCAGCAGCAGCAGCAGCAGCAGCAGCAGCAGCAGCAGCAGCAGCAGCAG**CAACAGCCG**CCACCGCCGCCGCCGCCGCCGCCGCCTCCTCAGCTTCCTCAGCCGCCGCCGCAGGCACAGCCGCTGCTGCCTCAGC
GCTGATGAAGGCCTTCGAGTCCCTCA---------------------------AGCAGCAGCAGCAGCAGCAGCAGCAGCAGCAGCAGCAGCAGCAGCAGCAGCAGCAGCAGCAGCAGCAGCAGCAGCAGCAGCAGCAGCAGCAGCAGCAGCAGCAGCAGCAGCAG**CAACAGCCG**CCACCGCCGCCGCCGCCGCCGCCGCCTCCTCAGCTTCCTCAGCCGCCGCCGCAGGCACAGCCGCTGCTGCCTCAGC 242 173 DEL
73 43.0
GCTGATGAAGGCCTTCGAGTCCCTCAAGTCCTTC--------CAGCAGCAGCAGCAGCAGCAGCAGCAGCAGCAGCAGCAGCAGCAGCAGCAGCAGCAGCAGCAGCAGCAGCAGCAGCAGCAGCAGCAGCAGCAGCAGCAGCAGCAGCAGCAGCAGCAGCAGCAGCAG**CAACAGCCG**CCACCGCCGCCGCCGCCGCCGCCGCCTCCTCAGCTTCCTCAGCCGCCGCCGCAGGCACAGCCGCTGCTGCCTCAGC
GCTGATGAAGGCCTTCGAGTCCCT**CAAGTCCTTCC**ACAGCAGCAGCAGCAGCAGCAGCAGCAGCAGCAGCAGCAGCAGCAGCAGCAGCAGCAGCAGCAGCAGCAGCAGCAGCAGCAGCAGCAGCAGCAGCAGCAGCAGCAGCAGCAGCAGCAGCAGCAGCAGCAGCAGCAG**CAACAGCCG**CCACCGCCGCCGCCGCCGCCGCCGCCTCCTCAGCTTCCTCAGCCGCCGCCGCAGGCACAGCCGCTGCTGCCTCAGC 242 170 INS
543 29.0
GCTGATGAAGGCCTTCGAGTCCCT**CAAGTCCTTCC**AGCAGCAGCAGCAGCAGCAGCAGCAGCAGCAGCAGCAGCAGCAGCAGCAGCAGCAGCAGCAGCAGCAGCAGCAGCAGCAGCAGCAGCAGCAGCAGCAGCAGCAGCAGCAGCAGCAGCAGCAGCAG**CAACAGCCG**CCACCGCCGCCGCCGCCGCCGCCGCCTCCTCAGCTTCCTCAGCCGCCGCCGCAGGCACAGCCGCTGCTGCCTCAGC
GCTGATGAAGGCCTTCGAGTCCCTCA--AGCAGCAGCAGCAGCAGCAGCAGCAGCAGCAGCAGCAGCAGCAGCAGCAGCAGCAGCAGCAGCAGCAGCAGCAGCAGCAGCAGCAGCAGCAGCAG**CAACAGCCG**CCACCGCCGCCGCCGCCGCCGCCGCCTCCTCAGCTTCCTCAGCCGCCGCCGCAGGCACAGCCGCTGCTGCCTCAGC 242 168 DEL
322 40.0
GCTGATGAAGGCCTTCGAGTCCCT**CAAGTCCTTCC**AGCAGCAGCAGCAGCAGCAGCAGCAGCAGCAGCAGCAGCAGCAGCAGCAGCAGCAGCAGCAGCAGCAGCAGCAGCAGCAGCAGCAGCAGCAGCAGCAGCAGCAGCAGCAGCAGCAGCAGCAGCAG**CAACAGCCG**CCACCGCCGCCGCCGCCGCCGCCGCCTCCTCAGCTTCCTCAGCCGCCGCCGCAGGCACAGCCGCTGCTGCCTCAGC

```
GCTGATGAAGGCCTTCGAGT---------------AGCAGCAGCAGCAGCAGCAGCAGCAGCAGCAGCAGCAGCAGCAGCAGCAGCAGCAGCAGCAGCAGCAGCAGCAGCAGCAGCAGCAGCAGCAGCAGCAGCAGCAGCAGCAGCAGCAGCAGCAGCAGCAGCAACAGCCGCCACCGCCGCCGCCGCCGCCGCCGCCTCCTCAGCTTCCTCAGCCGCCGCCGCAGGCACAGCCGCTGCTGCCTCAGC    242    162    DEL
562    40.0
GCTGATGAAGGCCTTCGAGTCCCTCAAGTCCTTCC-AGCAGCAGCAGCAGCAGCAGCAGCAGCAGCAGCAGCAGCAGCAGCAGCAGCAGCAGCAGCAGCAGCAGCAGCAGCAGCAGCAGCAGCAGCAGCAGCAGCAGCAGCAGCAGCAGCAGCAGCAGCAGCAACAGCCGCCACCGCCGCCGCCGCCGCCGCCGCCTCCTCAGC
GCTGATGAAGGCCTTCGAGTCCCTCAAGTCCTTCCAAGCAGCAGCAGCAGCAGCAGCAGCAGCAGCAGCAGCAGCAGCAGCAGCAGCAGCAGCAGCAGCAGCAGCAGCAGCAGCAGCAGCAGCAGCAGCAGCAGCAGCAGCAGCAGCAGCAGCAGCAGCAGCAACAGCCGCCACCGCCGCCGCCGCCGCCGCCG---CCTCAGC    200    157    INS+DEL


1532    0.0
GCTGATGAAGGCCTTCGAGTCCCTCAAGTCCTTCCAGCAGCAGCAGCAGCAGCAGCAGCAGCAGCAGCAGCAGCAGCAGCAGCAGCAGCAGCAGCAGCAGCAGCAGCAGCAGCAGCAGCAGCAGCAGCAGCAGCAGCAGCAGCAGCAGCAGCAGCAGCAGCAACAGCCGCCACCGCCGCCGCCGCCGCCGCCGCCTCCTCAGCTTCCTCAGCCGCCGCCGCAGGCACAGCCGCTGCTGCCTCAGC
GCTGATGAAGGCCTTCGAGTCCCTCAAGT-------------------------------------------------------------------------------------------------------------------------------------------------------------------CCGCCGCCGCAGGCACAGCCGCTGCTGCCTCAGC    242    157    DEL
1439    0.0
GCTGATGAAGGCCTTCGAGTCCCTCAAGTCCTTCCAGCAGCAGCAGCAGCAGCAGCAGCAGCAGCAGCAGCAGCAGCAGCAGCAGCAGCAGCAGCAGCAGCAGCAGCAGCAGCAGCAGCAGCAGCAGCAGCAGCAGCAGCAGCAGCAGCAGCAGCAGCAGCAACAGCCGCCACCGCCGCCGCCGCCGCCGCCGCCTCCTCAGCTTCCTCAGCCGCCGCCGCAGGCACAGCCGCTGCTGCCTCAGC
GCTGATGAAGGCCTTCGAGTCCCTCAAGTC----------------------------------------------------------------------------------------------------------------------------------------------------------------CCTCCTCAGCTTCCTCAGCCGCCGCCGCAGGCACAGCCGCTGCTGCCTCAGC    242    156    DEL
637    22.0
GCTGATGAAGGCCTTCGAGTCCCTCAAGTCCTTCCAGCAGCAGCAGCAGCAGCAGCAGCAGCAGCAGCAGCAGCAGCAGCAGCAGCAGCAGCAGCAGCAGCAGCAGCAGCAGCAGCAGCAGCAGCAGCAGCAGCAGCAGCAGCAGCAGCAGCAGCAGCAGCAACAGCCGCCACCGCCGCCGCCGCCGCCGCCGCCTCCTCAGCTTCCTCAGCCGCCGCCGCAGGCACAGCCGCTGCTGCCTCAGC
GCTGATGAAGGCCTTCGAGTCCCTCAAGTCCTTCCA-----------------------------------------------------AGCAGCAGCAGCAGCAGCAGCAGCAGCAGCAGCAGCAGCAGCAGCAGCAGCAGCAGCAGCAGCAGCAGCAGCAACAGCCGCCACCGCCGCCGCCGCCGCCGCCGCCTCCTCAGCTTCCTCAGCCGCCGCCGCAGGCACAGCCGCTGCTGCCTCAGC    242    155    DEL
745    22.0
GCTGATGAAGGCCTTCGAGTCCCTCAAGTCCTTCCAGCAGCAGCAGCAGCAGCAGCAGCAGCAGCAGCAGCAGCAGCAGCAGCAGCAGCAGCAGCAGCAGCAGCAGCAGCAGCAGCAGCAGCAGCAGCAGCAGCAGCAGCAGCAGCAGCAGCAGCAGCAGCAACAGCCGCCACCGCCGCCGCCGCCGCCGCCGCCTCCTCAGCTTCCTCAGCCGCCGCCGCAGGCACAGCCGCTGCTGCCTCAGC
GCTGATGAAGGCCTTCGAGTCCCT-------------------------------------------------------------------CAGCAGCAGCAGCAGCAGCAGCAGCAGCAGCAGCAGCAGCAGCAGCAGCAGCAGCAGCAGCAGCAGCAACAGCCGCCACCGCCGCCGCCGCCGCCGCCGCCTCCTCAGCTTCCTCAGCCGCCGCCGCAGGCACAGCCGCTGCTGCCTCAGC    242    148    DEL
949    1.0
GCTGATGAAGGCCTTCGAGTCCCTCAAGTCCTTCCAGCAGCAGCAGCAGCAGCAGCAGCAGCAGCAGCAGCAGCAGCAGCAGCAGCAGCAGCAGCAGCAGCAGCAGCAGCAGCAGCAGCAGCAGCAGCAGCAGCAGCAGCAGCAGCAGCAGCAGCAGCAGCAACAGCCGCCACCGCCGCCGCCGCCGCCGCCGCCTCCTCAGCTTCCTCAGCCGCCGCCGCAGGCACAGCCGCTGCTGCCTCAGC
GCTGATGAAGGCCTTCGAGTCCCTCAAGTCCTTCCGCCCTGAGCAGCAACAGCTG-------------------------------------------------------------------------------------------------AGCAGCAACAGCCGCCACCGCCGCCGCCGCCGCCGCCGCCTCCTCAGCTTCCTCAGCCGCCGCCGCAGGCACAGCCGCTGCTGCCTCAGC    242    147    DEL
611    23.0
GCTGATGAAGGCCTTCGAGTCCCTCAAGTCCTTCCAGCAGCAGCAGCAGCAGCAGCAGCAGCAGCAGCAGCAGCAGCAGCAGCAGCAGCAGCAGCAGCAGCAGCAGCAGCAGCAGCAGCAGCAGCAGCAGCAGCAGCAGCAGCAGCAGCAGCAGCAGCAGCAACAGCCGCCACCGCCGCCGCCGCCGCCGCCGCCTCCTCAGCTTCCTCAGCCGCCGCCGCAGGCACAGCCGCTGCTGCCTCAGC
GCTGATGAAGGCCTTCGAGTCCCTCAAGTCCTTCCA--------------------------------------------------AGCAGCAGCAGCAGCAGCAGCAGCAGCAGCAGCAGCAGCAGCAGCAGCAGCAGCAGCAGCAGCAGCAGCAGCAGCAACAGCCGCCACCGCCGCCGCCGCCGCCGCCGCCTCCTCAGCTTCCTCAGCCGCCGCCGCAGGCACAGCCGCTGCTGCCTCAGC    242    139    DEL
511    40.0
GCTGATGAAGGCCTTCGAGTCCCTCAAGTCCTTCC-AGCAGCAGCAGCAGCAGCAGCAGCAGCAGCAGCAGCAGCAGCAGCAGCAGCAGCAGCAGCAGCAGCAGCAGCAGCAGCAGCAGCAGCAGCAGCAGCAGCAGCAGCAGCAGCAGCAGCAGCAGCAGCAACAGCCGCCACCGCCGCCGCCGCCGCCGCCGCCTCCTCAGCTTCCTCAGCCGCCGCCGCAGGCACAGCCGCTGCTGCCTCAGC
GCTGATGAAGGCCTTCGAGTCCCTCAAGTCCTTCCAAGCAGCAGCAGCAGCAGCAGCAGCAGCAGCAGCAGCAGCAGCAGCAGCAGCAGCAGCAGCAGCAGCAGCAGCAGCAGCAGCAGCAGCAGCAGCAGCAGCAGCAGCAGCAGCAGCAGCAGCAGCAGCAACAGCCGCCA---------------------------------------CCGCCGCCGCAGGCACAGCCGCTGCTGCCTCAGC    242    133    INS+DEL
408    31.0
GCTGATGAAGGCCTTCGAGTCCCTCAAGTCCTTCCAGCAGCAGCAGCAGCAGCAGCAGCAGCAGCAGCAGCAGCAGCAGCAGCAGCAGCAGCAGCAGCAGCAGCAGCAGCAGCAGCAGCAGCAGCAGCAGCAGCAGCAGCAGCAGCAGCAGCAGCAGCAGCAACAGCCGCCACCGCCGCCGCCGCCGCCGCCGCCTCCTCAGCTTCCTCAGCCGCCGCCGCAGGCACAGCCGCTGCTGCCTCAGC
GCTGATGAAGGCCTTCGAGTCCCTCAAGTCCTTCCAG-------------------------AGCAGCAGCAGCAGCAGCAGCAGCAGCAGCAGCAGCAGCAGCAGCAGCAGCAGCAGCAGCAGCAGCAGCAGCAGCAGCAGCAGCAGCAGCAGCAGCAGCAACAGCCGCCACCGCCGCCGCCGCCGCCGCCGCCTCCTCAGCTTCCTCAGCCGCCGCCGCAGGCACAGCCGCTGCTGCCTCAGC    242    130    DEL
59    44.0
GCTGATGAAGGCCTTCGAGTCCCTCAAGTCCTTC-------------CAGCAGCAGCAGCAGCAGCAGCAGCAGCAGCAGCAGCAGCAGCAGCAGCAGCAGCAGCAGCAGCAGCAGCAGCAGCAGCAGCAGCAGCAGCAGCAGCAGCAGCAGCAGCAGCAGCAGCAGCAGCAGCAACAGCCGCCACCGCCGCCGCCGCCGCCGCCGCCTCCTCAGCTTCCTCAGCCGCCGCCGCAGGCACAGCCGCTGCTGCCTCAGC
GCTGATGAAGGCCTTCGAGTCCCTCAAGTCCTTCCAAGCAGCAGCAGCAGCAGCAGCAGCAGCAGCAGCAGCAGCAGCAGCAGCAGCAGCAGCAGCAGCAGCAGCAGCAGCAGCAGCAGCAGCAGCAGCAGCAGCAGCAGCAGCAGCAGCAGCAGCAGCAGCAGCAGCAGCAGCAACAGCCGCCACCGCCGCCGCCGCCGCCGCCGCCTCCTCAGCTTCCTCAGCCGCCGCCGCAGGCACAGCCGCTGCTGCCTCAGC    242    129    INS
652    24.0
GCTGATGAAGGCCTTCGAGTCCCTCAAGTCCTTCCAGCAGCAGCAGCAGCAGCAGCAGCAGCAGCAGCAGCAGCAGCAGCAGCAGCAGCAGCAGCAGCAGCAGCAGCAGCAGCAGCAGCAGCAGCAGCAGCAGCAGCAGCAGCAGCAGCAGCAGCAGCAGCAACAGCCGCCACCGCCGCCGCCGCCGCCGCCGCCTCCTCAGCTTCCTCAGCCGCCGCCGCAGGCACAGCCGCTGCTGCCTCAGC
GCTGATGAAGGCCTTCGAGTCCCTCAAGTC-------------------------------------------------------CAGCAGCAGCAGCAGCAGCAGCAGCAGCAGCAGCAGCAGCAGCAGCAGCAGCAGCAGCAGCAGCAGCAGCAGCAACAGCCGCCACCGCCGCCGCCGCCGCCGCCGCCTCCTCAGCTTCCTCAGCCGCCGCCGCAGGCACAGCCGCTGCTGCCTCAGC    242    126    DEL
1216    0.0
GCTGATGAAGGCCTTCGAGTCCCTCAAGTCCTTCCAGCAGCAGCAGCAGCAGCAGCAGCAGCAGCAGCAGCAGCAGCAGCAGCAGCAGCAGCAGCAGCAGCAGCAGCAGCAGCAGCAGCAGCAGCAGCAGCAGCAGCAGCAGCAGCAGCAGCAGCAGCAGCAACAGCCGCCACCGCCGCCGCCGCCGCCGCCGCCTCCTCAGCTTCCTCAGCCGCCGCCGCAGGCACAGCCGCTGCTGCCTCAGC
GCTGATGAAGGCCTTCGAGTCCCT-------------------------------------------------------------------------------------------------------------------------------------CAACAGCCGCCACCACCGCCGCCGCCGCCGCCGCCTCCTCAGCTTCCTCAGCCGCCGCCGCAGGCACAGCCGCTGCTGCCTCAGC    242    126    DEL
628    25.0
GCTGATGAAGGCCTTCGAGTCCCTCAAGTCCTTCCAGCAGCAGCAGCAGCAGCAGCAGCAGCAGCAGCAGCAGCAGCAGCAGCAGCAGCAGCAGCAGCAGCAGCAGCAGCAGCAGCAGCAGCAGCAGCAGCAGCAGCAGCAGCAGCAGCAGCAGCAGCAGCAACAGCCGCCACCGCCGCCGCCGCCGCCGCCGCCTCCTCAGCTTCCTCAGCCGCCGCCGCAGGCACAGCCGCTGCTGCCTCAGC
GCTGATGAAGGCCTTCGAGTCCCTCAAGTC----------------------------------------------------CAGCAGCAGCAGCAGCAGCAGCAGCAGCAGCAGCAGCAGCAGCAGCAGCAGCAGCAGCAGCAGCAGCAGCAGCAGCAACAGCCGCCACCGCCGCCGCCGCCGCCGCCGCCTCCTCAGCTTCCTCAGCCGCCGCCGCAGGCACAGCCGCTGCTGCCTCAGC    242    125    DEL
399    33.0
GCTGATGAAGGCCTTCGAGTCCCTCAAGTCCTTCCAGCAGCAGCAGCAGCAGCAGCAGCAGCAGCAGCAGCAGCAGCAGCAGCAGCAGCAGCAGCAGCAGCAGCAGCAGCAGCAGCAGCAGCAGCAGCAGCAGCAGCAGCAGCAGCAGCAGCAGCAGCAGCAACAGCCGCCACCGCCGCCGCCGCCGCCGCCGCCTCCTCAGCTTCCTCAGCCGCCGCCGCAGGCACAGCCGCTGCTGCCTCAGC
GCTGATGAAGGCCTTCGAGTCCCTCAAGTCCTTC------------------------CAGCAGCAGCAGCAGCAGCAGCAGCAGCAGCAGCAGCAGCAGCAGCAGCAGCAGCAGCAGCAGCAGCAGCAGCAGCAGCAGCAGCAGCAGCAGCAGCAGCAGCAACAGCCGCCACCGCCGCCGCCGCCGCCGCCGCCTCCTCAGCTTCCTCAGCCGCCGCCGCAGGCACAGCCGCTGCTGCCTCAGC    242    123    DEL
585    39.0
GCTGATGAAGGCCTTCGAGTCCCTCAAGTCCTTCCAGCAGCAGCAGCAGCAGCAGCAGCAGCAGCAGCAGCAGCAGCAGCAGCAGCAGCAGCAGCAGCAGCAGCAGCAGCAGCAGCAGCAGCAGCAGCAGCAGCAGCAGCAGCAGCAGCAGCAGCAGCAGCAACAGCCGCCACCGCCGCCGCCGCCGCCGCCGCCTCCTCAGCTTCCTCAGCCGCCGCCGCAGGCACAGCCGCTGCTGCCTCAGC
GCTGATGAAGGCCTTCGAGTCCCTCAAGTCCTTCCA--AGCAGCAGCAGCAGCAGCAGCAGCAGCAGCAGCAGCAGCAGCAGCAGCAGCAGCAGCAGCAGCAGCAGCAGCAGCAGCAGCAGCAGCAGCAGCAGCAGCAGCAGCAGCAGCAGCAGCAGCAACAGCCGCCACCGCCGCCGCCGCCGCCGCCG---CCTCAGCTTCCTCAGCCGCCGCCGCAGNCACAGCCGCTGCTGCCTCAGC    242    121    DEL
883    11.0
GCTGATGAAGGCCTTCGAGTCCCTCAAGTCCTTCCAGCAGCAGCAGCAGCAGCAGCAGCAGCAGCAGCAGCAGCAGCAGCAGCAGCAGCAGCAGCAGCAGCAGCAGCAGCAGCAGCAGCAGCAGCAGCAGCAGCAGCAGCAGCAGCAGCAGCAGCAGCAGCAACAGCCGCCACCGCCGCCGCCGCCGCCGCCGCCTCCTCAGCTTCCTCAGCCGCCGCCGCAGGCACAGCCGCTGCTGCCTCAGC
GCTGATGAAGGCCTTCGAGTCCCTCAAGTCCTTCCA--------------------------------------------------------------------------------------AGCAGCAGCAGCAGCAGCAGCAGCAGCAGCAGCAGCAACAGCCGCCACCGCCGCCGCCGCCGCCGCCGCCTCCTCAGCTTCCTCAGCCGCCGCCGCAGGCACAGCCGCTGCTGCCTCAGC    242    115    DEL
738    21.0
GCTGATGAAGGCCTTCGAGTCCCTCAAGTCCTTCCAGCAGCAGCAGCAGCAGCAGCAGCAGCAGCAGCAGCAGCAGCAGCAGCAGCAGCAGCAGCAGCAGCAGCAGCAGCAGCAGCAGCAGCAGCAGCAGCAGCAGCAGCAGCAGCAGCAGCAGCAGCAGCAACAGCCGCCACCGCCGCCGCCGCCGCCGCCGCCTCCTCAGCTTCCTCAGCCGCCGCCGCAGGCACAGCCGCTGCTGCCTCAGC
GCTGATGAAGGCCTTCGAGTCCCTCA------------------------------------------------------------------AGCAGCAGCAGCAGCAGCAGCAGCAGCAGCAGCAGCAGCAGCAGCAGCAGCAGCAGCAGCAGCAGCAGCAACAGCCGCCACCGCCGCCGCCGCCGCCGCCGCCTCCTCAGCTTCCTCAGCCGCCGCCGCAGGCACAGCCGCTGCTGCCTCAGC    242    114    DEL
1373    0.0
GCTGATGAAGGCCTTCGAGTCCCTCAAGTCCTTCCAGCAGCAGCAGCAGCAGCAGCAGCAGCAGCAGCAGCAGCAGCAGCAGCAGCAGCAGCAGCAGCAGCAGCAGCAGCAGCAGCAGCAGCAGCAGCAGCAGCAGCAGCAGCAGCAGCAGCAGCAGCAGCAACAGCCGCCACCGCCGCCGCCGCCGCCGCCGCCTCCTCAGCTTCCTCAGCCGCCGCCGCAGGCACAGCCGCTGCTGCCTCAGC
GCTGATGAAGGCCTTCGAGTCCCTCAAGTCCTTCC-------------------------------------------------------------------------------------------------------------------------------------------------------TCCGCCTCCTCAGCTTCCTCAGCCGCCGCCGCAGGCACAGCCGCTGCTGCCTCAGC    242    111    DEL
452    31.0
GCTGATGAAGGCCTTCGAGTCCCTCAAGTCCTTCCAGCAGCAGCAGCAGCAGCAGCAGCAGCAGCAGCAGCAGCAGCAGCAGCAGCAGCAGCAGCAGCAGCAGCAGCAGCAGCAGCAGCAGCAGCAGCAGCAGCAGCAGCAGCAGCAGCAGCAGCAGCAGCAACAGCCGCCACCGCCGCCGCCGCCGCCGCCGCCTCCTCAGCTTCCTCAGCCGCCGCCGCAGGCACAGCCGCTGCTGCCTCAGC
GCTGATGAAGGCCTTCGAGTCCCTCAAGTCCTTC------------------------------CAGCAGCAGCAGCAGCAGCAGCAGCAGCAGCAGCAGCAGCAGCAGCAGCAGCAGCAGCAGCAGCAGCAGCAGCAGCAGCAGCAGCAGCAGCAGCAACAGCCGCCACCGCCGCCGCCGCCGCCGCCGCCTCCTCAGCTTCCTCAGCCGCCGCCGCAGGCACAGCCGCTGCTGCCTCAGC    242    104    DEL
1326    0.0
GCTGATGAAGGCCTTCGAGTCCCTCAAGTCCTTCCAGCAGCAGCAGCAGCAGCAGCAGCAGCAGCAGCAGCAGCAGCAGCAGCAGCAGCAGCAGCAGCAGCAGCAGCAGCAGCAGCAGCAGCAGCAGCAGCAGCAGCAGCAGCAGCAGCAGCAGCAGCAGCAACAGCCGCCACCGCCGCCGCCGCCGCCGCCGCCTCCTCAGCTTCCTCAGCCGCCGCCGCAGGCACAGCCGCTGCTGCCTCAGC
GCTGATGAAGGCCTTCGAGTC-------------------------------------------------------------------------------------------------------------------------------------------------CCACCGCCGCCGCCGCCGCCGCCGCCTCCTCAGCTTCCTCAGCCGCCGCCGCAGGCACAGCCGCTGCTGCCTCAGC    242    104    DEL
40    47.0
GCTGATGAAGGCCTTCGAGTCCCTCAAGTCCTTC----------------------CAGCAGCAGCAGCAGCAGCAGCAGCAGCAGCAGCAGCAGCAGCAGCAGCAGCAGCAGCAGCAGCAGCAGCAGCAGCAGCAGCAGCAGCAGCAGCAGCAGCAGCAGCAGCAGCAGCAGCAGCAGCAGCAGCAACAGCCGCCACCGCCGCCGCCGCCGCCGCCGCCTCCTCAGCTTCCTCAGCCGCCGCCGCAGGCACAGCCGCTGCTGCCTCAGC
GCTGATGAAGGCCTTCGAGTCCCTCAAGTCCTTCCAAGCAGCAGCAGCAGCAGCAGCAGCAGCAGCAGCAGCAGCAGCAGCAGCAGCAGCAGCAGCAGCAGCAGCAGCAGCAGCAGCAGCAGCAGCAGCAGCAGCAGCAGCAGCAGCAGCAGCAGCAGCAGCAGCAGCAGCAGCAGCAGCAGCAGCAGCAACAGCCGCCACCGCCGCCGCCGCCGCCGCCGCCTCCTCAGCTTCCTCAGCCGCCGCCGCAGGCACAGCCGCTGCTGCCTCAGC  242    102    INS
1539    0.0
GCTGATGAAGGCCTTCGAGTCCCTCAAGTCCTTCCAGCAGCAGCAGCAGCAGCAGCAGCAGCAGCAGCAGCAGCAGCAGCAGCAGCAGCAGCAGCAGCAGCAGCAGCAGCAGCAGCAGCAGCAGCAGCAGCAGCAGCAGCAGCAGCAGCAGCAGCAGCAGCAACAGCCGCCACCGCCGCCGCCGCCGCCGCCGCCTCCTCAGCTTCCTCAGCCGCCGCCGCAGGCACAGCCGCTGCTGCCTCAGC
GCTGATGAAGGCCTTCGAGTCCCTCA------------------------------------------------------------------------------------------------------------------------------------------------------------------------------------AGCCGCCGCCGCAGGCACAGCCGCTGCTGCCTCAGC    242    102    DEL
```
