# Supplementary material for: CRISPR/Cas9-induced double-strand breaks in the huntingtin locus lead to CAG repeat contraction through DNA end resection and homology-mediated repair
Source: BMC Biol. 2024 Dec 3;22:282. doi: 10.1186/s12915-024-02079-6 (PMC11616332; doi:10.1186/s12915-024-02079-6)
Supplement: Supplementary file 4 — Additional file 4. Uncropped images of gels and western blots. [file 12915_2024_2079_MOESM4_ESM.pdf]

Additional File 4

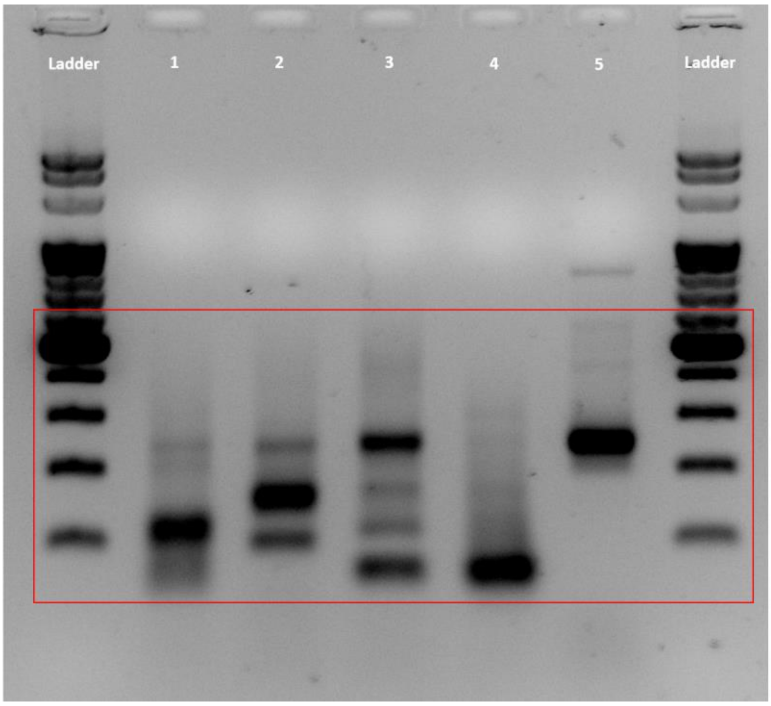

1. HTT_gRNA1
2. HTT_gRNA2
3. HTT_gRNA4
4. HTT_gRNA1 + HTT_gRNA4
5. control (unedited)

Figure 1 uncropped gel

ctrl HTT_ gRNA2

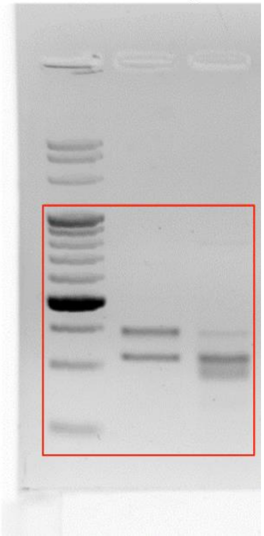

Figure 2 uncropped gel

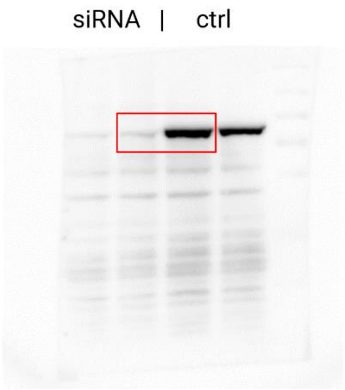

Supplementary Figure S2 uncropped blot- MRE11

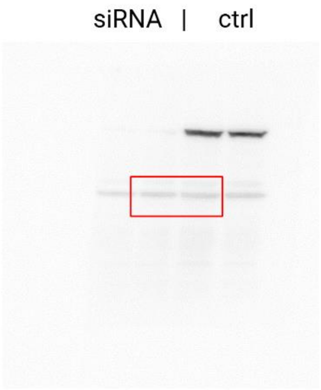

Supplementary Figure S2 uncropped blot- β-actin control for MRE11

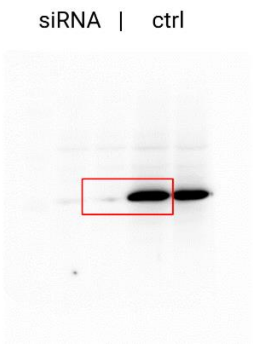

Supplementary Figure S2 uncropped blot- RAD51

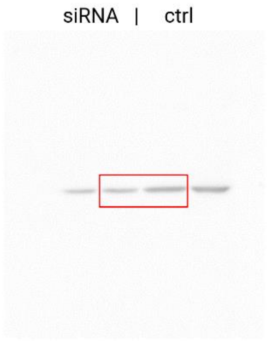

Supplementary Figure S2 uncropped blot- β-actin control for RAD51

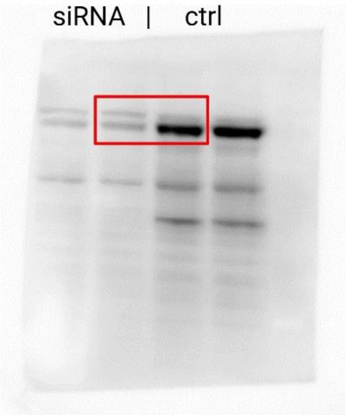

Supplementary Figure S2 uncropped blot- Artemis

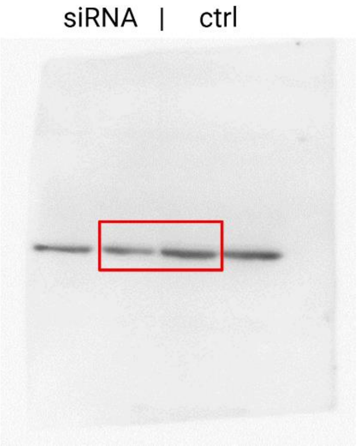

Supplementary Figure S2 uncropped blot- β-actin control for Artemis

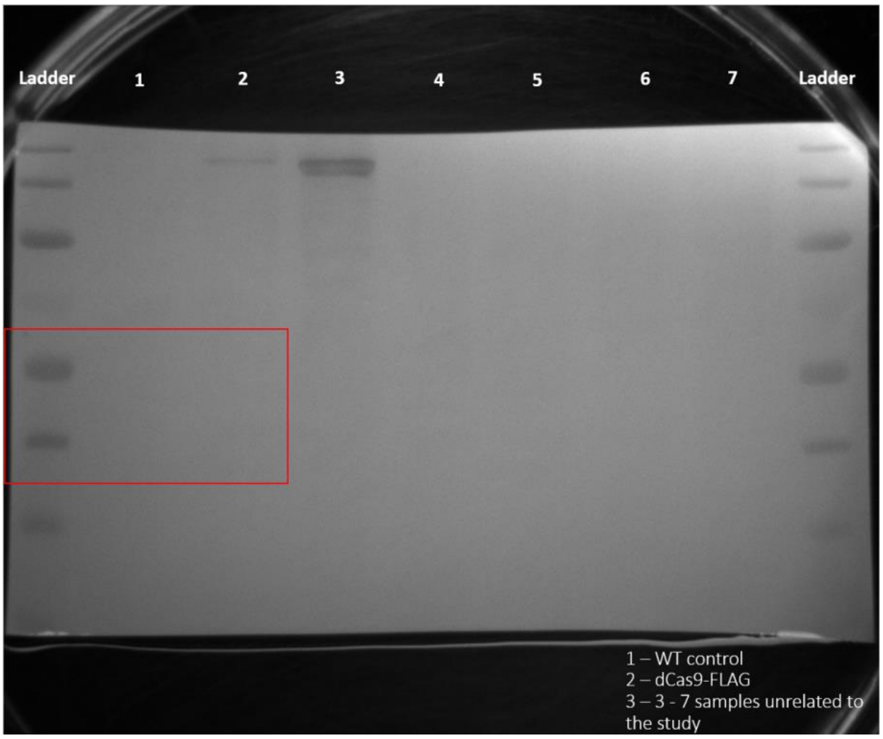

Supplementary Figure S3 uncropped blot – b-actin1

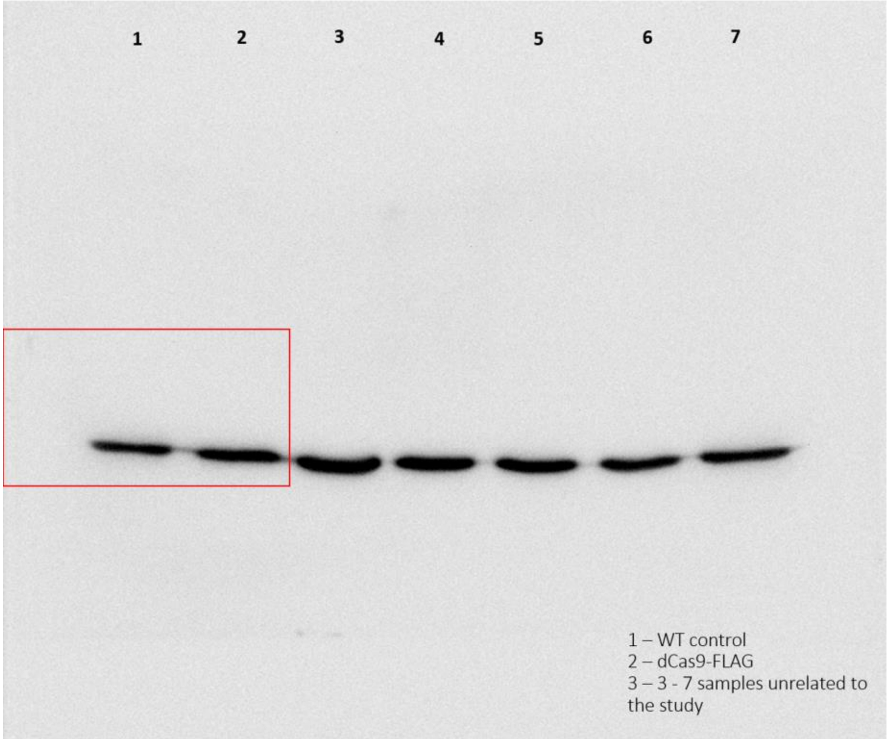

Supplementary Figure S3 uncropped blot – b-actin2

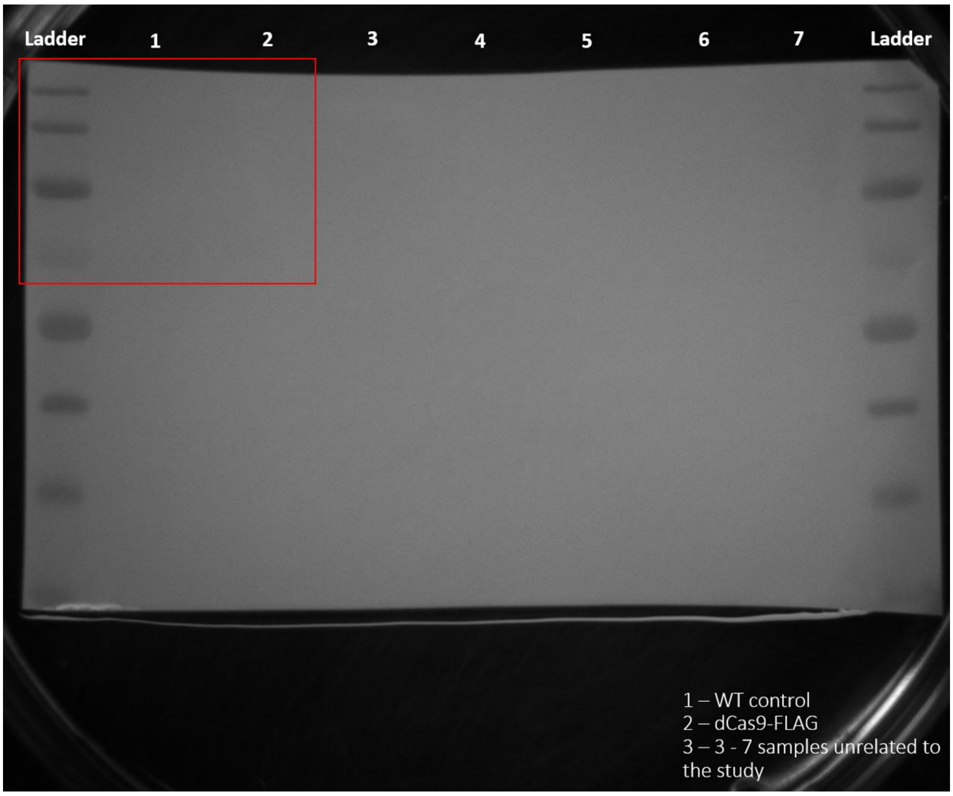

Supplementary Figure S3 uncropped blot – dCas9

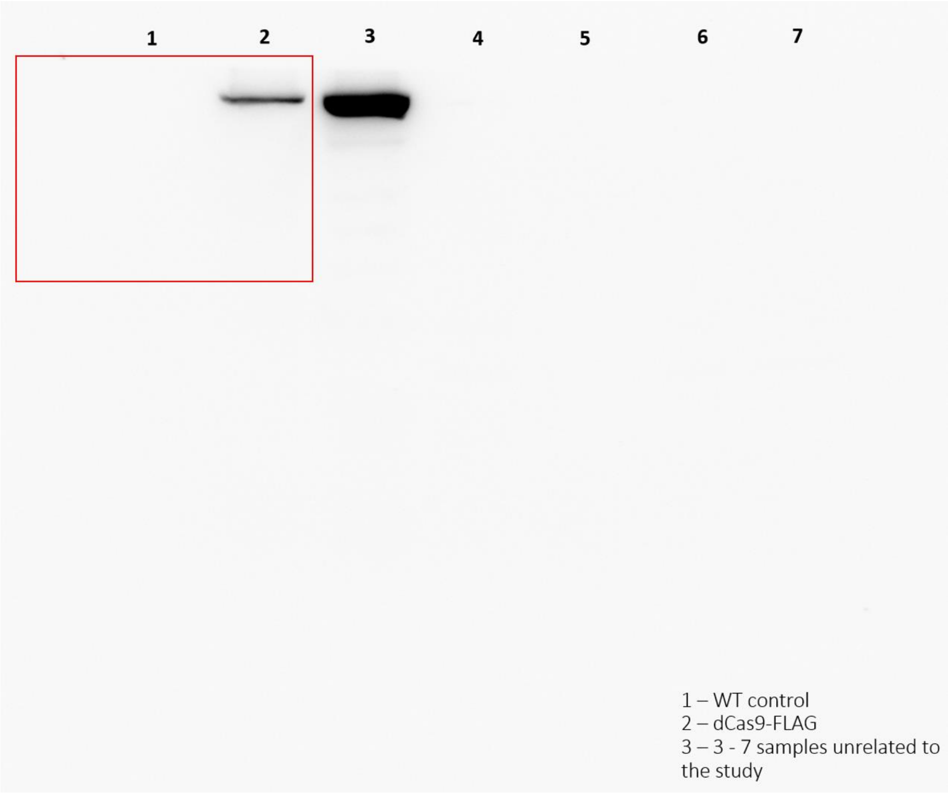

Supplementary Figure S3 uncropped blot - dCas9
